# Supplementary material for: Association of social isolation, loneliness and genetic risk with incidence of dementia: UK Biobank Cohort Study
Source: BMJ Open. 2022 Feb 23;12(2):e053936. doi: 10.1136/bmjopen-2021-053936 (PMC8867309; doi:10.1136/bmjopen-2021-053936)
Supplement: Supplementary data [file bmjopen-2021-053936supp001.pdf]

Supplement: Elovainio, M, Lahti, J., Pirinen, M., Pulkki-Råback, L., Lipsanen, J., Virtanen, M., Kivimäki, M., & Hakulinen, C. The association between social isolation, loneliness, and genetic risk with incidence of dementia: UK Biobank cohort study

Table of content

1) Additional information of dementia assessment
2) Additional information of genetic risk score
3) The associations between genetic risk score and incident dementia using 10 various genetic risk score cut-off points
4) Interaction effects
5) The associations of social isolation, loneliness and genetic risk score with specific Alzheimer's disease
6) The associations of social isolation, loneliness and genetic risk score with incident dementia using imputed data

1) Additional information of dementia assessment
Incident all-cause dementia was defined using the following ICD-9 and ICD-10 codes:

ICD-9: 290.2, 290.3, 290.4, 291.2, 294.1, 331.0, 331.1, 331.2. 331.5

ICD-10: A81.0, F00, F00.0, F00.1, F00.2, F00.9, F01, F01.0, F01.1, F01.2, F01.3, F01.8, F01.9, F02, F02.0, F02.1, F02.2, F02.3, F02.4, F02.8, F03, F05.1, F10.6, G30, G30.0, G30.1, G30.8, G30.9, G31.0, G31.1, G31.8, I67.3

Incident Alzheimer's disease was defined using the following ICD-9 and ICD-10 codes:

ICD-9: 331.0

ICD-10: F00, F00.0, F00.1, F00.2, F00.9, G30, G30.0, G30.1, G30.8, G30.9

For more information of the dementia assessment see:
http://biobank.ndph.ox.ac.uk/showcase/showcase/docs/alg_outcome_dementia.pdf

2) Additional information of genetic risk score

International Genomics of Alzheimer's Project (IGAP) is a large three-stage study based upon genome-wide association studies (GWAS) on individuals of European ancestry. In stage 1, IGAP used genotyped and imputed data on 11,480,632 single nucleotide polymorphisms (SNPs) to meta-analyse GWAS datasets consisting of 21,982 Alzheimer's disease cases and 41,944 cognitively normal controls from four consortia: The Alzheimer Disease Genetics Consortium (ADGC); The European Alzheimer's disease Initiative (EADI); The Cohorts for Heart and Aging Research in Genomic Epidemiology Consortium (CHARGE); and The Genetic and Environmental Risk in AD Consortium Genetic and Environmental Risk in AD/Defining Genetic, Polygenic and Environmental Risk for Alzheimer's Disease Consortium (GERAD/PERADES). In stage 2, 11,632 SNPs were genotyped and tested for association in an independent set of 8,362 Alzheimer's disease cases and 10,483 controls. Meta-analysis of variants selected for analysis in stage 3A (n = 11,666) or stage 3B (n = 30,511) samples brought the final sample to 35,274 clinical and autopsy-documented Alzheimer's disease cases and 59,163 controls.

3) The associations between genetic risk score and incident dementia using 10 various geneic risk score cut-off points

The associations between continuous PRS and incident dementia with various cut-off points is reported in SFigure 1 below. The bars are negative log10 -transformed p-values of the PRS-dementia association.

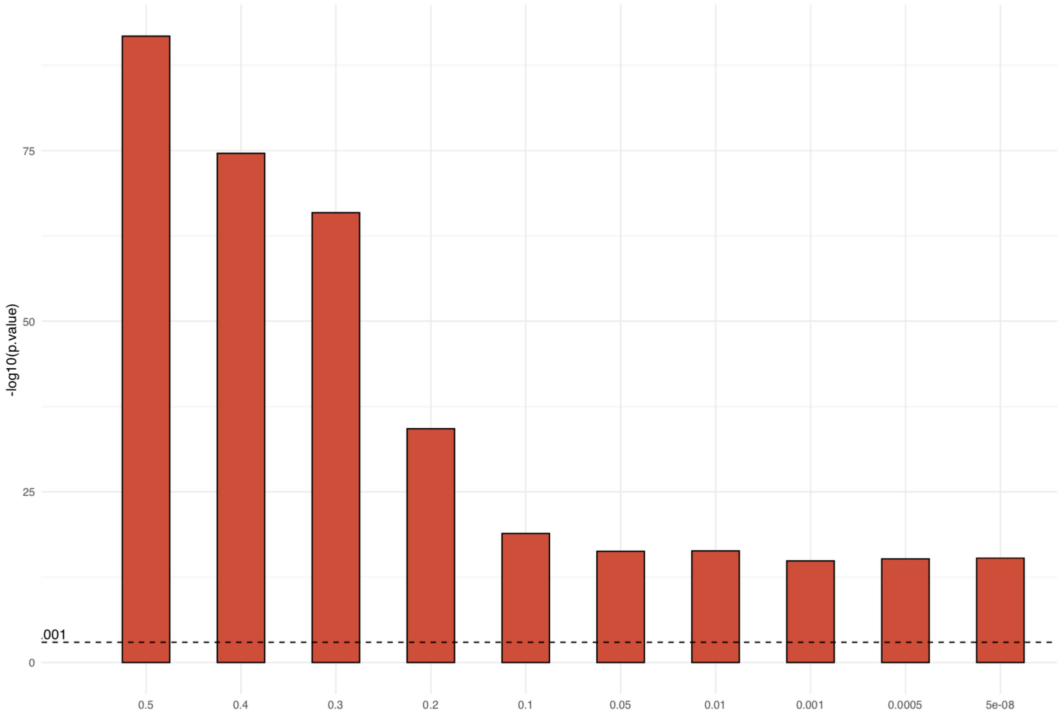

4) Interaction effects

Stable1a : Sex -genetic risk -interactions (adjusted for main effects). Figures and Hazar Ratios (HR) and 95 % confidence intervals (95% CI)

	All dementia		Alzheimer's disease	
Predictor	*HR (95% CI)*	*P-Value*	*HR (95% CI)*	*P-Value*
Sex (male) * intermediate genetic risk	0.82 (0.62 – 1.08)	0.151	0.76 (0.48 – 1.20)	0.238
Sex (male) * high genetic risk	0.84 (0.64 – 1.10)	0.197	0.74 (0.48 – 1.14)	0.168
Observations	155070		155070	

Stable 1b : Sex - loneliness and sex -isolation -interactions (adjusted for main effects). Figures and Hazar Ratios (HR) and 95 % confidence intervals (95% CI)

	All dementia		Alzheimer's disease	
Predictor	*HR (95% CI)*	*P-Value*	*HR (95% CI)*	*P-Value*
Sex (male) * lonelyd	1.36 (0.90 – 2.04)	0.143	2.63 (1.18 – 5.90)	0.019
Sex (male) * isolated	0.90 (0.66 – 1.24)	0.533	1.13 (0.68 – 1.90)	0.640
Observations	147610 /152719		147610 / 152719	

STable 1c: Genetic risk - loneliness and genetic risk -isolation -interactions (adjusted for age, sex and main effects). Figures and Hazar Ratios (HR) and 95 % confidence intervals (95% CI)

	All dementia		Alzheimer's disease	
Predictor	*HR (95%CI)*	*P-Value*	*HR (95%CI)*	*P-Value*
Intermediate genetic risk *lonelyd	0.86 (0.51 – 1.47)	0.586	1.71 (0.45 – 6.62)	0.431
High genetic risk * lonelyd	0.98 (0.58 – 1.65)	0.945	3.25 (0.94 – 11.28)	0.063
Observations	147610		147610	

	All dementia		Alzheimer's disease	
Predictor	*HR (95%CI)*	*P-Value*	*HR (95%CI)*	*P-Value*

Intermediate genetic risk * isolated	1.18<br>(0.77 – 1.81)	0.449	1.22<br>(0.59 – 2.52)	0.587
High genetic risk * isolated	1.11<br>(0.73 – 1.69)	0.624	1.15<br>(0.58 – 2.31)	0.682
Observations	152719		152719	

5) The associations of social isolation, loneliness and genetic risk score with specific Alzheimer's disease

We repeated all the analyses using specific Alzheimer's disease as the outcome instead of incident dementia and the results were materially the same, although there were, of course, much less Alzheimer's disease cases.

STable 2. Risk of Incident Alzheimers' Disease According to Genetic Risk

	**Model 1**		**Model 2**	
Genetic risk	*HR 95 % CI*	*P-Value*	*HR 95 % CI*	*P-Value*
Intermediate	1.51 (1.20 – 1.90)	**<0.001**	1.42 (1.11 – 1.82)	**<0.001**
High	1.98 (1.59 – 2.45)	**<0.001**	1.91 (1.51 – 2.41)	**<0.001**
Observations	155063		139345	

Model 1. Adjusted for age, sex, and 10 principal components
Model 2. Adjusted for age, sex, 10 principal components, education, social deprivation, depressive symptoms, health behaviors, loneliness and social isolation

STable 3. Associations of loneliness and isolation with Alzheimer' disease. The figures are Hazard ratios (HR) and 95% confidence intervals (95% CI)

	**Model 1**		**Model 2**		**Model 3**	
Separate analyses						
Predictor	*HR (95% CI)*	*P-Value*	*HR (95% CI)*	*P-Value*	*HR (95% CI)*	*P-Value*
Lonely vs not lonely	1.05 (0.72 – 1.52)	0.809	0.85 (0.55 – 1.31)	0.450	0.86 (0.55 – 1.33)	0.503
Isolated vs no isolated	1.56 (1.21 – 2.02)	**<0.001**	1.40 (1.05 – 1.88)	**0.024**	1.41 (1.05 – 1.89)	**0.021**
Combined analyses						
	HR (95% CI)	*P-Value*	*HR (95% CI)*	*P-Value*	*HR (95% CI)*	*P-Value*
Lonely vs not lonely	0.95 (0.64 – 1.40)	0.774	0.84 (0.51 – 1.26)	0.346	0.81 (0.52 – 1.27)	0.716
Isolated vs no isolated	1.54 (1.18 – 2.02)	**0.002**	1.41 (1.04 – 1.91)	**0.025**	1.42 (1.05 – 1.93)	**0.022**
Observations	147604 /152712		133885 /137894		133885 /137894	

Model 1. Adjusted for age and sex
Model 2. Adjusted for age, sex, education, social deprivation, health behaviours, long-term illness, depressive symptoms, genetic risk and 10 principal components
Model 3. Adjusted for age, sex, education, social deprivation, health behaviours, long-term illness, depressive symptoms, and apolipoprotein E genotype.

6) The associations of social isolation, loneliness and genetic risk score with incident dementia using imputed data

The number of missing values was relatively small (only less the 5% had missing values), but we repeated the final models using five imputed data sets and, not surprisingly, the results were materially not changed (sTable 4).

STable 4. Associations of loneliness and isolation with incident dementia with imputed data. The figures are Hazard ratios (HR) and 95% confidence intervals (95% CI)

	**Model 1**		**Model 2**		**Model 3**	
Separate analyses						
Predictor	*HR (95% CI)*	*P-Value*	*HR (95% CI)*	*P-Value*	*HR (95% CI)*	*P-Value*
Lonely vs not lonely	1.44 (1.17 – 1.77)	**<0.001**	1.01 (0.81 – 1.27)	0.901	1.02 (0.82 – 1.28)	0.832
Isolated vs no isolated	1.63 (1.39 – 1.92)	**<0.001**	1.34 (1.14 – 1.58)	**0.001**	1.34 (1.14 – 1.58)	**0.001**
Combined analyses						
	HR (95% CI)	*P-Value*	*HR (95% CI)*	*P-Value*	*HR (95% CI)*	*P-Value*
Lonely vs not lonely	1.32 (1.08 – 1.63)	**0.013**	0.97 (0.78 – 1.21)	0.804	0.98 (0.78 – 1.22)	0.844
Isolated vs no isolated	1.58 (1.35 – 1.86)	**<0.001**	1.35 (1.14– 1.59)	**0.002**	1.35 (1.15 – 1.60)	**0.003**
Observations	155063		155063		155063	

Model 1. Adjusted for age and sex
Model 2. Adjusted for age, sex, education, social deprivation, health behaviours, long-term illness, depressive symptoms, genetic risk and 10 principal components
Model 3. Adjusted for age, sex, education, social deprivation, health behaviours, long-term illness, depressive symptoms, and apolipoprotein E genotype.
