# Supplementary material for: Association of social isolation, loneliness and genetic risk with incidence of dementia: UK Biobank Cohort Study
Source: BMJ Open. 2022 Feb 23;12(2):e053936. doi: 10.1136/bmjopen-2021-053936 (PMC8867309; doi:10.1136/bmjopen-2021-053936)
Supplement: Author's manuscript [file bmjopen-2021-053936.draft_revisions.pdf]

BMJ Open is committed to open peer review. As part of this commitment we make the peer review history of every article we publish publicly available.

When an article is published we post the peer reviewers' comments and the authors' responses online. We also post the versions of the paper that were used during peer review. These are the versions that the peer review comments apply to.

The versions of the paper that follow are the versions that were submitted during the peer review process. They are not the versions of record or the final published versions. They should not be cited or distributed as the published version of this manuscript.

BMJ Open is an open access journal and the full, final, typeset and author-corrected version of record of the manuscript is available on our site with no access controls, subscription charges or pay-per-view fees (http://bmjopen.bmj.com).

If you have any questions on BMJ Open's open peer review process please email info.bmjopen@bmj.com

BMJ Open

Association of social isolation, loneliness, and genetic risk with incidence of dementia: UK Biobank cohort study

Journal:	*BMJ Open*
Manuscript ID	bmjopen-2021-053936
Article Type:	Original research
Date Submitted by the Author:	28-May-2021
Complete List of Authors:	Elovainio, Marko; University of Helsinki; Finnish Institute for Health and Welfare<br>Lahti, Jari; University of Helsinki<br>Pirinen, Matti; University of Helsinki; University of Helsinki, Department of Public Health<br>Pulkki-Raback, Laura; University of Helsinki, Psychology and logopedics<br>Malmberg, Anni; University of Helsinki<br>Lipsanen, Jari; University of Helsinki, Psychology and logopedics<br>Virtanen, Marianna; Itä-Suomen yliopisto, School of Educational Sciences and Psychology<br>Kivimaki, Mika; University College London, Department of Epidemiology & Public Health<br>Hakulinen, Christian; University of Helsinki, Psychology and logopedics
Keywords:	Dementia < NEUROLOGY, Public health < INFECTIOUS DISEASES, GENETICS, GERIATRIC MEDICINE

SCHOLARONE™
Manuscripts

I, the Submitting Author has the right to grant and does grant on behalf of all authors of the Work (as defined in the below author licence), an exclusive licence and/or a non-exclusive licence for contributions from authors who are: i) UK Crown employees; ii) where BMJ has agreed a CC-BY licence shall apply, and/or iii) in accordance with the terms applicable for US Federal Government officers or employees acting as part of their official duties; on a worldwide, perpetual, irrevocable, royalty-free basis to BMJ Publishing Group Ltd ("BMJ") its licensees and where the relevant Journal is co-owned by BMJ to the co-owners of the Journal, to publish the Work in this journal and any other BMJ products and to exploit all rights, as set out in our licence.

The Submitting Author accepts and understands that any supply made under these terms is made by BMJ to the Submitting Author unless you are acting as an employee on behalf of your employer or a postgraduate student of an affiliated institution which is paying any applicable article publishing charge ("APC") for Open Access articles. Where the Submitting Author wishes to make the Work available on an Open Access basis (and intends to pay the relevant APC), the terms of reuse of such Open Access shall be governed by a Creative Commons licence – details of these licences and which Creative Commons licence will apply to this Work are set out in our licence referred to above.

Other than as permitted in any relevant BMJ Author's Self Archiving Policies, I confirm this Work has not been accepted for publication elsewhere, is not being considered for publication elsewhere and does not duplicate material already published. I confirm all authors consent to publication of this Work and authorise the granting of this licence.

For peer review only

28 May 2021

Association of social isolation, loneliness, and genetic risk with incidence of dementia: UK Biobank cohort study

Marko Elovainio, PhD[1,2]
Jari Lahti, PhD[1]
Matti Pirinen, PhD[3,4,5]
Laura Pulkki-Råback, PhD [1,6]
Anni Malmberg, BA[1]
Jari Lipsanen, MSci [1]
Marianna Virtanen, PhD [7]
Mika Kivimäki, FMedSci [4,8]
Christian Hakulinen, PhD [1]

[1]Medical Research Program Unit, Faculty of Medicine, University of Helsinki, Finland;
[2]Finnish Institute for Health and Welfare, Finland;
[3]Institute for Molecular Medicine Finland, Helsinki Institute of Life Sciences, University of Helsinki;
[4]Department of Public Health, University of Helsinki, Finland;
[5]Helsinki Institute for Information Technology and Department of Mathematics and Statistics, University of Helsinki, Finland;
[6]Research Centre of Child Psychiatry, Faculty of Medicine, University of Turku, Finland
[7]School of Educational Sciences and Psychology, University of Eastern Finland, Joensuu, Finland;
[8]Department of Epidemiology and Public Health, University College London, London, UK;

Correspondence: Marko Elovainio, University of Helsinki, P.O. Box 9, 00014, Helsinki, Finland, Phone: +358 50 3020621, email: marko.elovainio@helsinki.fi
Word count for abstract / main text: 200 / 3259; Number of tables and figures: 4 +2

Keywords: Social network, social support, dementia, cognitive decline, public health

For peer review only

Background: Social isolation and loneliness have been associated with increased risk of dementia, but it is not known whether this risk is modified or confounded by genetic risk of dementia.

Methods: We used the prospective UK Biobank study with 155 070 participants (mean age 64.1 years), including self-reported social isolation and loneliness. Genetic risk was indicated using the polygenic risk score for Alzheimer's disease and the incident dementia ascertained using electronic health records.

Results: Overall, 8.6% of participants reported that they were socially isolated and 5.5% were lonely. During a mean follow-up of 8.8 years (1.36 million person-years), 1444 (0.9% of the total sample) were diagnosed with dementia. Social isolation, but not loneliness, was associated with increased risk of dementia (hazard ratio 1.62, 95% confidence interval 1.38 to 1.90). Of the participants who were socially isolated and had high genetic risk, 4.2% (2.9% to 5.5%) were estimated to develop dementia compared with 3.1% (2.7% to 3.5%) in participants who were not socially isolated but had high genetic risk. The corresponding estimated incidence in the socially isolated and not isolated were 3.9% (3.1% to 4.6%) and 2.5% (2.2% to 2.6%) in participants with intermediate genetic risk.

Conclusions: Socially isolated individuals are at increased risk of dementia at all levels of genetic risk.

For peer review only

What is already known on this topic

- Social isolation and loneliness have been associated with increased risk of dementia
- It is not known whether this risk is modified or confounded by genetic risk of dementia

What this study adds

- This is the first study to show that social isolation is associated with increased risk of dementia across the spectrum of genetic risk.
- Loneliness, in contrast to social isolation, seems to be associated with dementia only when combined with high genetic risk.

Article summary

- We showed that socially isolated individuals have higher risk for dementia across the spectrum of genetic risk.
- This study suggests that social isolation is a risk factor of its own, over and above genetic risk.

For peer review only

Strengths and limitations of the study

- The strengths of the study were its large sample size and a genome-wide study using a well-established polygenic risk score for dementia.
- This is the first study to show the combined effect of social isolation, loneliness, and genetic risk with dementia.
- Despite the large sample size, the sample was not representative of the UK population.
- As dementia was derived from hospital records, people with non-diagnosed dementia may have been missed.
- Reverse causation may have affected the findings by making people with pre-clinical dementia more socially isolated.
- Future research should examine the mechanistic pathways whereby social isolation is associated with dementia.

For peer review only

The rapidly rising numbers of people with dementia [1] is a significant health policy and health service concern in many high-income countries. Although considerable share of the dementia risk is due to genetic factors [2, 3], major efforts have been directed towards the identification of potentially modifiable risk factors that could prevent or delay the onset of dementia [4]. Higher levels of social support have been suggested to protect from dementia [5], with both social isolation and feelings of loneliness being associated with increased risk of dementia [6-8], although mixed findings have been reported between loneliness and dementia risk [9, 10]. However, it remains unclear whether there is an interplay between genetic factors and social isolation and loneliness (*i.e.* whether the association of social isolation and loneliness with dementia is evident only at high or low levels of genetic risk) or whether the associations of genetic factors and social support with dementia are independent and additive.

The polygenic risk score (PRS) for Alzheimer's disease, describing the polygenic burden captured by the most recent genome-wide studies [11], allows to estimate the size of the genetic risk and the extent to which the associations of social isolation and loneliness with dementia are modified by genetic risk. In the present study, we used data from UK Biobank study to examine whether genetic risk may intensify and attenuate the associations of social isolation and loneliness with the risk of dementia. In addition to estimating relative risk, we will provide estimates of absolute risk [12], as they are important information for risk communication and clinical risk prediction [13].

METHODS

Study design and participants

In this analysis of the UK Biobank study, we used baseline data and obtained information of incident dementia at follow-up via linked electronic health records [14]. UK National Health Service (NHS) registers maintain records of all individuals legally registered as residents in the

For peer review only

United Kingdom. In the UK Biobank study, these records were used to invite around 9.2 million individuals aged 40–69 years living within a sensible travelling distance of the 22 assessment centres across Great Britain 2007–2010 [14]. At the study baseline, participants completed multiple touchscreen computer-based questionnaires followed by a face-to-face interview with trained research staff. Physical measures were also taken. Details of these assessments and variables are publicly available from the UK Biobank website: http://biobank.ctsu.ox.ac.uk/crystal/.

In total, 502,656 individuals were recruited (5.4% of the eligible population). Of those, individuals that were 60 year or older and had complete data on social isolation, loneliness, dementia and genetic data were included in the present analysis (N = 147 614 – 152 070). We also repeated the analyses using imputed data in those with missing information on social isolation, loneliness or other explanatory variables but had information on genetic risk score (N = 155 070). This study was conducted under generic approval from the NHS National Research Ethics Service (17th June 2011, Ref 11/NW/0382). Participants provided electronic consent for the baseline assessments and register linkage.

Ascertainment of incident dementia

Dementia was ascertained using hospital inpatient records which contains data on admissions and diagnoses from the Hospital Episode Statistics for England, Scottish Morbidity Record data for Scotland, and the Patient Episode Database for Wales. Additional cases were detected through linkage to death register data provided by the National Health Service Digital for England and Wales and the Information and Statistics Division for Scotland. Diagnoses were recorded using the International Classification of Diseases (ICD) coding system. Participants with dementia were identified as having a primary/secondary diagnosis (hospital records) or underlying/contributory cause of death (death register) using ICD-9 and ICD-10 codes for Alzheimer disease and other dementia classifications (see supplement for details).

Measurement of social isolation and loneliness

For peer review only

Social isolation and loneliness were measured using the same scale as in our two previous UK Biobank studies [15, 16]. *Social isolation* scale was defined using the following three questions: (a) “Including yourself, how many people are living together in your household? Include those who usually live in the house such as students living away from home during term, partners in the armed forces or professions such as pilots” (1 point for living alone) (b) “How often do you visit friends or family or have them visit you?” (1 point for friends and family visits less than once a month), and (c) “Which of the following [leisure/social activities] do you attend once a week or more often? You can select more than one", (1 point for no participation in social activities at least weekly). This resulted in scale with a range from 0 to 3, where an individual was defined as socially isolated if he/she had two or more of those points and those who scored 0 or 1 were classified as not isolated. Other studies in the UK have used similar measures [16].

Loneliness scale was constructed from two questions: "Do you often feel lonely? " (no = 0, yes=1) and “"How often are you able to confide in someone close to you?"(0 = almost daily-once every few months 1= once every few months to never or almost never). An individual was defined as lonely if he/she responded positively to both questions (score 2) and not lonely if he or she responded negatively to one or both of the questions (score 0 -1). Similar questions have been used in longer loneliness scales, such as the Revised UCLA Loneliness Scale [17].

Polygenic risk score of dementia

From the genotyped UK Biobank samples, we included 155,070 unrelated white British participants after removal of participants based on heterozygosity and missingness of outliers, sex chromosome aneuploidies and mismatches, withdrawals, and those that UK Biobank had excluded from the relatedness calculations. The genotypes were imputed against Haplotype Reference Consortium and

For peer review only

UK10K haplotype resources containing ~96M variants [11]. We calculated polygenic risk scores (PRS) for Alzheimer's disease (AD) based on a genome-wide association study by Kunkle and others (2019) with 35,274 AD cases and 59,163 controls that do not overlap with UK Biobank samples (for details see the online supplement). We used Plink 1.9 [18] for the genotype QC and clumping. The following parameters were used for the clumping of the genotype data: p-value threshold 0.5, LD threshold (r^2) 0.5, and clumping window width of 250 kilobases. Prior to clumping we excluded all SNPs with MAF < 0.001, genotyping rate < 0.1, Hardy-Weinberg equilibrium p-value < 1e-6 and missingness per person >0.1. We used PRSice 2.2.8 [19] for calculating the PRS with the genotype QC settings that have been recommended by the software developers [20]. In the main analyses, we applied a p-value threshold of 0.5, which resulted in including 626,623 SNPs in the PRS. This threshold was chosen as previous work has reported that it provided an optimal set of variants for predicting dementia and AD [21, 22]. While this set is likely to include a number of variants which are not associated with AD, it also includes a number of variants that at present do not have sufficient statistical evidence to meet the criteria for being genome-wide significant (i.e. P-value < 5x10-8) but are expected to be associated in future larger studies. The univariate associations between genetic risks score with 10 different cut-off points and incident dementia is reported in the supplement (SFigure 1).

The polygenic risk scores were then z-standardized to have mean 0 and variance 1, divided into quintiles and categorized as low (lowest quintile), intermediate (quintiles 2 to 4) and high (highest quintile).

Assessment of potential explanatory factors

Following information was used in the current study: sex, age in years, socioeconomic factors (educational attainment and Townsend deprivation index, which is an area-level composite measure of deprivation based on unemployment, non-home ownership, non-car ownership, and household

For peer review only

overcrowding), chronic diseases (diabetes, cardiovascular disease, cancer, and other long-standing illness, disability or infirmity), cigarette smoking (smoker [yes/no]; ex-smoker[yes/no]), physical activity (moderate and vigorous physical activity), alcohol intake frequency (Three or four times a week or more vs. once or twice a week or less), and the frequency of depressed mood in the past 2 weeks (Patient Health Questionnaire; [23]).

Statistical analyses

Study participants were followed from the study baseline (2006-2010) for incident dementia until the date of first dementia diagnosis, death, or to the end of the follow-up, whichever came first. The associations of social isolation, loneliness and polygenic risk score with incident dementia were examined using Cox proportional hazard regression models where age was used as a time scale. Results from these analyses were reported as hazard ratios (relative risk) and their 95% confidence intervals and the models were adjusted for age, sex, and 10 first principal components of genetic structure from UK Biobank to control for possible population stratification, and additionally for education, social deprivation index, having long term illness, physical activity, smoking status, alcohol consumption, and depressive symptoms. In these analyses, PRS was used both as a categorical and as a continuous variable. Cumulative incidence (absolute risk) of dementia associated with categories of social isolation, loneliness and genetic risk was estimated using competing-risks regression [24, 25], with death being treated as competing event.

Missing data on social isolation, loneliness and all explanatory factors were imputed using multiple imputation by chained equations to generate five imputed datasets. Imputation model included age, sex, social isolation, loneliness, all covariates, the Nelson-Aalen estimate of cumulative hazard, and survival status [26]. Cox proportional hazards models were fitted within each imputed dataset and combined using Rubin's rules. Frequencies of complete and imputed

For peer review only

variables are reported in supplement table 3. P-values were 2-sided with statistical significance set at less than .05. All analyses were performed using Stata (15.1) and R (3.6.2).

Role of the funding source

The sponsors of the study had no role in study design, data collection, data analysis, data interpretation, or writing of the report. Elovainio and Hakulinen had full access to the data. Elovainio and Hakulinen take final responsibility for the decision to submit for publication.

Patient involvement

These results are based on existing data. We were not involved in the recruitment of the participants. As far as we know, no patients were engaged in designing the present research question or the outcome measures. They were also not involved in developing plans for recruitment, design, or implementation of the study, and were not asked to advise on interpretation or writing up of results. Results from UK Biobank are disseminated to study participants via the study website and social media outlets.

RESULTS

Descriptive statistics of the study participants are shown in **Table 1**. Genetic risk score data were available for 155 070 participants (51.9% women; mean age 64.1 years). Overall, 8.6% of participants (N = 13103) were classified as socially isolated and 5.5% were lonely (N = 8102). During a total of 1.36 million person-years (mean follow-up time 8.8 years), 1444 participants (0.9% of the total sample) were diagnosed with all-cause dementia.

As expected, a higher PRS for AD was associated with an increased risk of dementia. Using continuous PRS, the hazard ratio per 1SD increase in the score was 1.27 (95% CI 1.21 to 1.34) in an analysis adjusted for age, sex and 10 principal components. The associations between genetic risk categories (low, intermediate, and high) with incidence of dementia shown in **Table 2**. In comparison to the participants in the low genetic risk category, the hazard ratio of incident dementia was 1.56 (95% CI 1.31 – 1.87) in participants with intermediate risk and 1.89 (95% CI

For peer review only

1.55 – 2.31) in those with high genetic risk in the fully adjusted model.

Social isolation was associated with increased risk of dementia (HR adjusted for age and sex = 1.62, 95% CI 1.38 – 1.90). The associations attenuated but remained statistically significant after adjusting for additional covariates including socio-demographics, health-related factors and genetic risk score and principal components (HR = 1.33, 95% CI 1.12 – 1.60). Loneliness was also associated with higher risk of dementia (HR = 1.47, 95% CI 1.20 – 1.80), but this association was lost when adjusted for socio-demographics, health-related factors, PRS and principal components (HR = 1.03, 95% CI 0.81 – 1.30). Both social isolation (HR = 1.58, 95% CI 1.34 – 1.86) and loneliness (HR = 1.28, 95% CI 1.03 – 1.59) were associated with incident dementia when added simultaneously into the model but only the association between social isolation and dementia was robust to adjusting for additional covariates (HR = 1.33, 95 % CI 1.10 – 1.60). (**Table 2**)

When the interplay between genetic risk and social isolation was assessed using combined categories, there was a monotonic association of increasing genetic risk and social isolation with increasing dementia risk. At low genetic risk for dementia, the socially isolated participants had a higher risk for dementia than the non-isolated participants (hazard ratio = 1.42, 95% CI, 0.88-2.27. . The hazard ratios for dementia were 1.57, (95% CI 1.30 - 1.89) and 1.99, 95% CI 1.61 - 2.45) for those with intermediate or high genetic risk and no social isolation, and 2.16, (95% CI 1.65 – 2.83) and 2.37, 95% CI 1.62 – 3.46) for those who were socially isolated and had intermediate or high genetic risk (**Figure 1**). The results for loneliness were less consistent, although the risk of dementia was greater in lonely participants at low or at high levels of genetic risk, when compared with those who reported no loneliness. In the high genetic risk group, for example, the hazard ratios were 1.93 (95% CI 1.56 - 2.37) in low and 2.20 (95% CI 1.39 - 3.47) in high loneliness group (**Figure 2**).

For peer review only

In terms of absolute risk (cumulative incidence), of those who were socially isolated and had high genetic risk, 4.2% (2.9% to 5.5%) were estimated to developed dementia compared with 3.1% (2.7% to 3.5%) of those who were not socially isolated but had high genetic risk (**Figure 3**). The corresponding absolute risk estimates in the socially isolated and not isolated were 3.9% (3.1% to 4.6%) and 2.4% (2.2% to 2.6%) in participants with intermediate genetic risk and 2.2% (1.2% to 3.1%) and 1.5% (1.2% to 1.7%) in those with low genetic risk.

As sensitivity analyses, we repeated all the main analyses with Alzheimer's disease as the outcome and using imputed data sets (Supplement SFigures 2 – 3). The results did not materially change.

DISCUSSION

In this UK Biobank study of 155 074 men and women, social isolation was associated with increased risk of all-cause dementia and Alzheimer's disease at all levels of genetic risk of Alzheimer's disease. The incidence of dementia was estimated to reach over 4% in isolated high-genetic risk individuals compared to approximately 3% in non-isolated individuals with similar genetic risk. The difference between these groups was over 1% also among those with intermediate and low genetic risk. This means that among individuals with similar genetic risk for dementia, those who are socially isolated are more likely to have incidence of the disease, suggesting an effect by social isolation over and above that of genetic risk. The association between loneliness and dementia was attributable to other dementia risk factors, such as health behaviours and depressive symptoms.

To the best of our knowledge, this is the first study examining the joint associations of aspects of social support and genetic risk with the incidence of dementia. The relative risk of dementia across the genetic risk categories was at the same magnitude as in a previous UK Biobank study [27] that used data from an older GWAS [28]. Our findings also support other studies - most

For peer review only

of which with follow-ups from 5 to 11 years – showing an association of social isolation with increased risk of dementia [6, 8, 10]. A 28-year follow-up of 10,000 Whitehall II study participants found that less frequent social contacts at ages 50, 60 and 70 were associated with approximately 10% higher dementia risk, independent of socio-economic and other lifestyle factors [29]. While previous studies have produced mixed findings on whether loneliness is associated with increased risk of dementia or not [9, 10], our findings show that the association between loneliness and dementia is mostly likely explained by other factors and present only at high levels of genetic risk.

Our results should be interpreted in a context of disease aetiology. Dementia is characterised by a 10-20-year preclinical or prodromal stage during which changes in biomarkers and cognitive abilities increasingly occur [30]. With a follow-up less than 10 years, it is likely that we assessed social isolation for dementia cases during this preclinical period. This could result to reverse causality, i.e., increased prevalence of social isolation during the 8-year period could have resulted from preclinical changes in social activity leading to a spurious association between social isolation and dementia.

Several mechanisms through which social isolation may causally affect dementia risk have been proposed. Social isolation and loneliness have been suggested to increase stress reactivity which is associated with prolonged activation of the hypothalamic-pituitary-adrenal axis (HPA) and the sympatho-adrenal system [31]. This process may further lead to sleep deprivation, dysregulation of the immune system, and even increased levels of oxidative stress [32], all potentially harmful for cognitive health. It has also been shown that socially isolated and lonely individuals more often engage in health-damaging behaviors [16], which may affect cognition either directly via biophysiological mechanisms or increased incidence of cardiometabolic diseases which accelerate neurodegeneration [33]. Socially isolated or lonely individuals are also at an increased risk of depression [34], a potential risk factor for cognitive decline and dementia [35]. Participation in social activities and social interaction stimulates neural plasticity by building and maintaining

For peer review only

cognitive reserve [36]. Poor cognitive reserve is a further pathway through which social isolation and loneliness could increase dementia risk [37]. Fewer social contacts with reduced exercising of memory and language adversely affect cognitive reserve, thereby accelerating dementia onset [37]. Cognitive ability was not assessed in the present study and a small share of the found association between social isolation and subsequent dementia risk may be attributable to lower initial cognitive reserve.

Strengths and limitations

The major strengths of the current study include the large sample size of UK Biobank participants, which enabled us to study the combination of genetic risk, social isolation, and loneliness in detail. In addition, we used the largest genome-wide association study of dementia to date to derive the genetic risk for AD [2].

There are also some important limitations. Although our analyses were adjusted for multiple potential sources of bias, the possibility of unmeasured confounding and reverse causation cannot be ruled out. Both frequency of social contacts and loneliness were self-reported and measured by relatively short and crude measures. As we were able to cover the genetic risk for AD – not all-cause dementias – based on the Kunkle et al [2], we may have missed some of the genetic variance related to non-AD dementias. Dementia cases were derived from medical records or death registers, and thus some cases might have been missed. However, good agreement of dementia case determination with primary care record data has been shown [38]. This sample was restricted to volunteers of European ancestry aged 60 to 73 years at baseline and, therefore, further research is needed to ensure generalizability of our findings. As the mean age of participants was only 72 years

For peer review only

at the end of the follow-up period, the incidence of dementia remained low. As noted previously the response rate of the UK Biobank study survey was very low, 5.5%, and UK Biobank is not representative of the sampling population [39]. However, many etiological findings from UK Biobank appear to be generalisable to England and Scotland [40].

Conclusions

The present findings suggest an association between social isolation and increased risk of dementia across the spectrum of genetic risk. Further research is needed to determine the extent social isolation is a modifiable risk factor rather than a part of the dementia prodrome

For peer review only

Acknowledgments: We thank the International Genomics of Alzheimer's Project (IGAP) for providing summary results data for these analyses. The investigators within IGAP contributed to the design and implementation of IGAP and/or provided data but did not participate in analysis or writing of this report. IGAP was made possible by the generous participation of the control subjects, the patients, and their families. The i–Select chips was funded by the French National Foundation on Alzheimer's disease and related disorders. EADI was supported by the LABEX (laboratory of excellence program investment for the future) DISTALZ grant, Inserm, Institut Pasteur de Lille, Université de Lille 2 and the Lille University Hospital. GERAD/PERADES was supported by the Medical Research Council (Grant n° 503480), Alzheimer's Research UK (Grant n° 503176), the Wellcome Trust (Grant n° 082604/2/07/Z) and German Federal Ministry of Education and Research (BMBF): Competence Network Dementia (CND) grant n° 01GI0102, 01GI0711, 01GI0420. CHARGE was partly supported by the NIH/NIA grant R01 AG033193 and the NIA AG081220 and AGES contract N01–AG–12100, the NHLBI grant R01 HL105756, the Icelandic Heart Association, and the Erasmus Medical Center and Erasmus University. ADGC was supported by the NIH/NIA grants: U01 AG032984, U24 AG021886, U01 AG016976, and the Alzheimer's Association grant ADGC–10–196728. Laura Pulkki-Råback was supported by the Jenny and Antti Wihuri Foundation.

For peer review only

Contributors: ME and CH designed the study and conducted the statistical analyses. ME wrote the first draft of the manuscript. JL and AM calculated the polygenetic risk score with the help of MP. All authors contributed to the interpretation of the results and critical revision of the manuscript for important intellectual content and approved the final version of the manuscript. The corresponding author attests that all listed authors meet authorship criteria and that no others meeting the criteria have been omitted.

Funding: ME and CH were supported by the Academy of Finland (339390 (ME) / 310591(CH)).

MK was supported by NordForsk (70521), the UK Medical Research Council (MRC S011676), the Academy of Finland (311492), and the US National Institutes on Ageing (NIA R01AG056477). The funding sources did not participate in the design or conduct of the study; collection, management, analysis or interpretation of the data; or preparation, review, or approval of the manuscript.

Competing interests: All authors have completed the ICMJE uniform disclosure form at www.icmje.org/coi_disclosure.pdf (available on request from the corresponding author) and declare: no support from any organisation for the submitted work, no other relationships or activities that could appear to have influenced the submitted work.

Ethical approval: Ethical approval for data collection was given by the North-West Multi-centre Research Ethics Committee. Study was carried out in accordance with the Declaration of Helsinki of the World Medical Association. The ethical board of the Finnish Institute for Health and Welfare gave ethical permission to use the genetic data.

Data sharing: The genetic and phenotypic UK Biobank data are available on application to the UK Biobank (www.ukbiobank.ac.uk/). Present study was conducted using the UK Biobank Resource under Application 14801. Summary statistics from the meta-analysis of genome wide association studies in dementia are available from https://www.niagads.org/datasets/ng00075

For peer review only

Transparency statement: The lead authors (ME and CH) affirms that the manuscript is an honest, accurate, and transparent account of the study being reported; that no important aspects of the study have been omitted; and that any discrepancies from the study as planned (and, if relevant, registered) have been explained.

For peer review only

References

1 Ahmadi-Abhari S, Guzman-Castillo M, Bandosz P*, et al.* Temporal trend in dementia incidence since 2002 and projections for prevalence in England and Wales to 2040: modelling study. *BMJ* 2017;**358**:j2856.

2 Kunkle BW, Grenier-Boley B, Sims R*, et al.* Genetic meta-analysis of diagnosed Alzheimer's disease identifies new risk loci and implicates Abeta, tau, immunity and lipid processing. *Nat Genet* 2019;**51**:414-30.

3 Andrews SJ, Fulton-Howard B, Goate A. Interpretation of risk loci from genome-wide association studies of Alzheimer's disease. *Lancet Neurol* 2020.

4 Soto-Gordoa M, Arrospide A, Moreno-Izco F*, et al.* Projecting Burden of Dementia in Spain, 2010-2050: Impact of Modifying Risk Factors. *J Alzheimers Dis* 2015;**48**:721-30.

5 Livingston G, Sommerlad A, Orgeta V*, et al.* Dementia prevention, intervention, and care. *Lancet* 2017;**390**:2673-734.

6 Kuiper JS, Zuidersma M, Oude Voshaar RC*, et al.* Social relationships and risk of dementia: A systematic review and meta-analysis of longitudinal cohort studies. *Ageing Res Rev* 2015;**22**:39-57.

7 Lara E, Caballero FF, Rico-Uribe LA*, et al.* Are loneliness and social isolation associated with cognitive decline? *Int J Geriatr Psychiatry* 2019.

8 Lara E, Martin-Maria N, De la Torre-Luque A*, et al.* Does loneliness contribute to mild cognitive impairment and dementia? A systematic review and meta-analysis of longitudinal studies. *Ageing Res Rev* 2019;**52**:7-16.

9 Penninkilampi R, Casey AN, Singh MF*, et al.* The Association between Social Engagement, Loneliness, and Risk of Dementia: A Systematic Review and Meta-Analysis. *J Alzheimers Dis* 2018;**66**:1619-33.

10 Sundstrom A, Nordin Adolfsson A, Nordin M*, et al.* Loneliness increases the risk of all-cause dementia and Alzheimer's disease. *The journals of gerontology Series B, Psychological sciences and social sciences* 2019.

11 Bycroft C, Freeman C, Petkova D*, et al.* The UK Biobank resource with deep phenotyping and genomic data. *Nature* 2018;**562**:203-9.

12 Torkamani A, Wineinger NE, Topol EJ. The personal and clinical utility of polygenic risk scores. *Nat Rev Genet* 2018;**19**:581-90.

13 Sugrue LP, Desikan RS. What Are Polygenic Scores and Why Are They Important? *JAMA* 2019;**321**:1820-1.

14 Palmer LJ. UK Biobank: bank on it. *Lancet* 2007;**369**:1980-2.

15 Elovainio M, Hakulinen C, Pulkki-Raback L*, et al.* Contribution of risk factors to excess mortality in isolated and lonely individuals: an analysis of data from the UK Biobank cohort study. *Lancet Public Health* 2017;**2**:E260-E6.

16 Hakulinen C, Pulkki-Raback L, Virtanen M*, et al.* Social isolation and loneliness as risk factors for myocardial infarction, stroke and mortality: UK Biobank cohort study of 479 054 men and women. *Heart* 2018.

17 Hughes ME, Waite LJ, Hawkley LC*, et al.* A Short Scale for Measuring Loneliness in Large Surveys: Results From Two Population-Based Studies. *Res Aging* 2004;**26**:655-72.

18 Purcell S, Neale B, Todd-Brown K*, et al.* PLINK: a tool set for whole-genome association and population-based linkage analyses. *Am J Hum Genet* 2007;**81**:559-75.

For peer review only

19 Choi SW, O'Reilly PF. PRSice-2: Polygenic Risk Score Software for Biobank-Scale Data. *GigaScience* 2019;**8**:July1, 2019.

20 Euesden J, Lewis CM, O'Reilly PF. PRSice: Polygenic Risk Score software. *Bioinformatics* 2015;**31**:1466-8.

21 Escott-Price V, Sims R, Bannister C*, et al.* Common polygenic variation enhances risk prediction for Alzheimer's disease. *Brain* 2015;**138**:3673-84.

22 Escott-Price V, Myers A, Huentelman M*, et al.* Polygenic Risk Score Analysis of Alzheimer's Disease in Cases without APOE4 or APOE2 Alleles. *J Prev Alzheimers Dis* 2019;**6**:16-9.

23 Spitzer RL, Kroenke K, Williams JB. Validation and utility of a self-report version of PRIME-MD: the PHQ primary care study. Primary Care Evaluation of Mental Disorders. Patient Health Questionnaire. *JAMA* 1999;**282**:1737-44.

24 Andersen PK, Geskus RB, de Witte T*, et al.* Competing risks in epidemiology: possibilities and pitfalls. *Int J Epidemiol* 2012;**41**:861-70.

25 Andersen PK, Keiding N. Interpretability and importance of functionals in competing risks and multistate models. *Stat Med* 2012;**31**:1074-88.

26 White I, Royston P. Imputing missing covariate values for the Cox model. *Statistical Medicine* 2009;**28**:1982-98.

27 Lourida I, Hannon E, Littlejohns TJ*, et al.* Association of Lifestyle and Genetic Risk With Incidence of Dementia. *JAMA* 2019.

28 Lambert JC, Ibrahim-Verbaas CA, Harold D*, et al.* Meta-analysis of 74,046 individuals identifies 11 new susceptibility loci for Alzheimer's disease. *Nature Genetics* 2013;**45**:1452-U206.

29 Sommerlad A, Sabia S, Singh-Manoux A*, et al.* Association of social contact with dementia and cognition: 28-year follow-up of the Whitehall II cohort study. *PLoS Med* 2019;**16**:e1002862.

30 Jack CR, Jr., Knopman DS, Jagust WJ*, et al.* Tracking pathophysiological processes in Alzheimer's disease: an updated hypothetical model of dynamic biomarkers. *Lancet Neurol* 2013;**12**:207-16.

31 McEwen BS. Allostasis, allostatic load, and the aging nervous system: role of excitatory amino acids and excitotoxicity. *Neurochem Res* 2000;**25**:1219-31.

32 Cacioppo S, Grippo AJ, London S*, et al.* Loneliness: clinical import and interventions. *Perspect Psychol Sci* 2015;**10**:238-49.

33 Kivimaki M, Singh-Manoux A, Pentti J*, et al.* Physical inactivity, cardiometabolic disease, and risk of dementia: an individual-participant meta-analysis. *BMJ* 2019;**365**:l1495.

34 Cacioppo JT, Hawkley LC, Thisted RA. Perceived social isolation makes me sad: 5-year cross-lagged analyses of loneliness and depressive symptomatology in the Chicago Health, Aging, and Social Relations Study. *Psychol Aging* 2010;**25**:453-63.

35 Diniz BS, Butters MA, Albert SM*, et al.* Late-life depression and risk of vascular dementia and Alzheimer's disease: systematic review and meta-analysis of community-based cohort studies. *Br J Psychiatry* 2013;**202**:329-35.

36 Scarmeas N, Stern Y. Cognitive reserve and lifestyle. *J Clin Exp Neuropsychol* 2003;**25**:625-33.

37 Berkman LF. Social support, social networks, social cohesion and health. *Soc Work Health Care* 2000;**31**:3-14.

38 Wilkinson T, Schnier C, Bush K*, et al.* Identifying dementia outcomes in UK Biobank: a validation study of primary care, hospital admissions and mortality data. *Eur J Epidemiol* 2019;**34**:557-65.

For peer review only

39 Fry A, Littlejohns TJ, Sudlow C, *et al.* Comparison of Sociodemographic and Health-Related Characteristics of UK Biobank Participants With Those of the General Population. *Am J Epidemiol* 2017;**186**:1026-34.
40 Batty GD, Gale CR, Kivimaki M, *et al.* Comparison of risk factor associations in UK Biobank against representative, general population based studies with conventional response rates: prospective cohort study and individual participant meta-analysis. *BMJ* 2020;**368**:m131.

For peer review only

2

Table 1. Baseline Characteristics of Participants according to diagnosed Dementia at follow-up

		Dementia		
Variables		No	Yes	p-value
Age at baseline	Mean (SD)	64.1 (2.8)	65.8 (2.7)	<0.001
Sex	Female	79821 (52.0)	631 (43.7)	<0.001
	Male	73805 (48.0)	813 (56.3)	
Education	Lower	40578 (26.7)	536 (38.2)	<0.001
	Intermediate	71839 (47.3)	606 (43.2)	
	Higher	39307 (25.9)	261 (18.6)	
Long term illness	No	57738 (38.7)	319 (23.3)	<0.001
	Yes	91266 (61.3)	1053 (76.7)	
Physical activity	Low	45963 (30.7)	479 (34.9)	0.001
	High	103938 (69.3)	893 (65.1)	
Current smoker	No	140646 (92.0)	1281 (89.4)	<0.001
	Yes	12265 (8.0)	152 (10.6)	
Alcohol consumption	Lower	81242 (52.9)	866 (60.1)	<0.001
	Higher	72283 (47.1)	575 (39.9)	
Depressive symptoms	Low	121508 (82.5)	1014 (75.8)	<0.001
	Low-medium	21350 (14.5)	245 (18.3)	
	High_medium	2788 (1.9)	42 (3.1)	
	High	1639 (1.1)	37 (2.8)	
Townsend deprivation index	Mean (SD)	-1.7 (2.8)	-1.1 (3.3)	<0.001
Socially isolated (no / yes)	No	138408 (91.5)	1208 (87.3)	<0.001
	Yes	12928 (8.5)	175 (12.7)	
Feelling lonely (no / yes)	No	138255 (94.5)	1253 (92.5)	0.001
	Yes	8000 (5.5)	102 (7.5)	
Genetic dementia risk	Low	30834 (20.1)	180 (12.5)	<0.001
	Intermediate	92148 (60.0)	895 (62.0)	
	High	30644 (19.9)	369 (25.5)	

2

Table 2. Risk of Incident Dementia According to Genetic Risk, Social Isolation and Loneliness Categories

	Model 1		Model 2	
Genetic risk	*Hazard Ratio 95 % CI*	*P-Value*	*Hazard Ratio 95 % CI*	*P-Value*
Intermediate vs. low	1.66 (1.41 – 1.94)	**<0.001**	1.56 (1.31 – 1.87)	**<0.001**
High vs. low	2.06 (1.72 – 2.46)	**<0.001**	1.89 (1.55 – 2.31)	**<0.001**
Observations	155074		132628	
R^2 Nagelkerke	0.345		0.461	

	Model 1		Model 2	
Separate analyses	*Hazard ratio 95 % CI*	*P-Value*	*Hazard ratio 95 % CI*	*P-Value*
Isolated vs no isolated	1.62 (1.38 – 1.90)	**<0.001**	1.33 (1.12 – 1.60)	**0.002**
Observations	152723		137903	
R^2 Nagelkerke	0.320		0.455	
Lonely vs not lonely	1.47 (1.20 – 1.80)	**<0.001**	1.03 (0.81 – 1.30)	0.820
Observations	147614		133893	
R^2 Nagelkerke	0.316		0.463	
Combined analysis				
Lonely	1.28 (1.03 – 1.59)	**0.025**	0.95 (0.74 – 1.22)	0.686

For peer review only

Isolated	1.58<br>(1.34 – 1.86)	**<0.001**	1.33<br>(1.10 – 1.60)	**0.003**
Observations	145663		132628	
R^2 Nagelkerke	0.322		0.461	

Model 1. Adjusted for age and sex
Model 2. Adjusted for age, sex, education, social deprivation, depressive symptoms, health behaviors, genetic risk score, and 10 principal components

For peer review only

Figure 1. Associations of combined genetic risk and social isolation with incident dementia risk.

Figure 2. Associations of combined genetic risk and loneliness with incident dementia risk.

Figure3. Estimated cumulative incidence of dementia in combined genetic risk and social isolation groups.

For peer review only

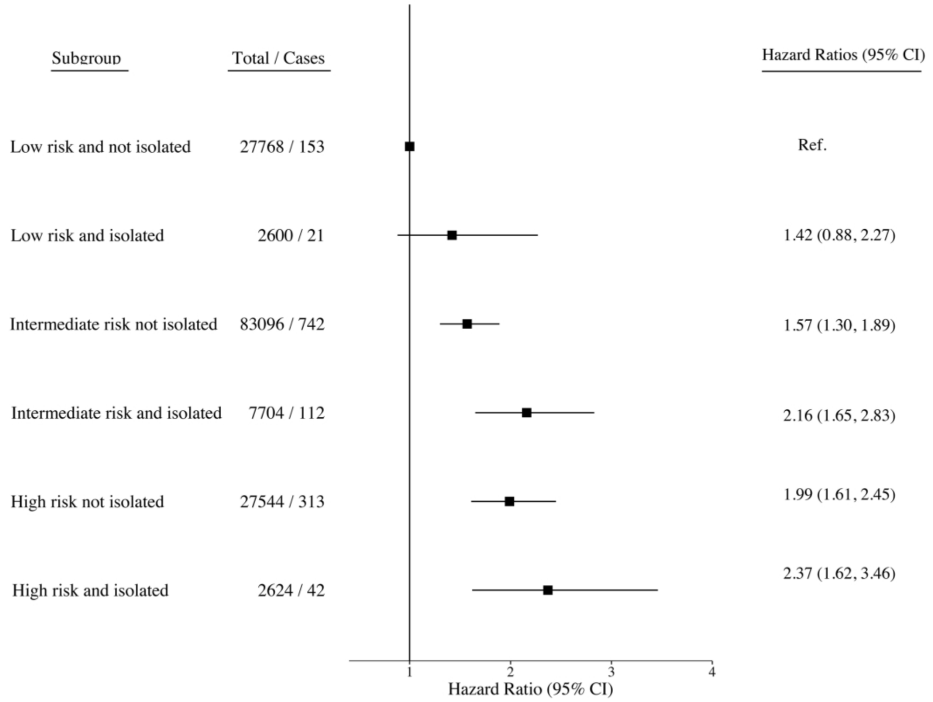

215x152mm (600 x 600 DPI)

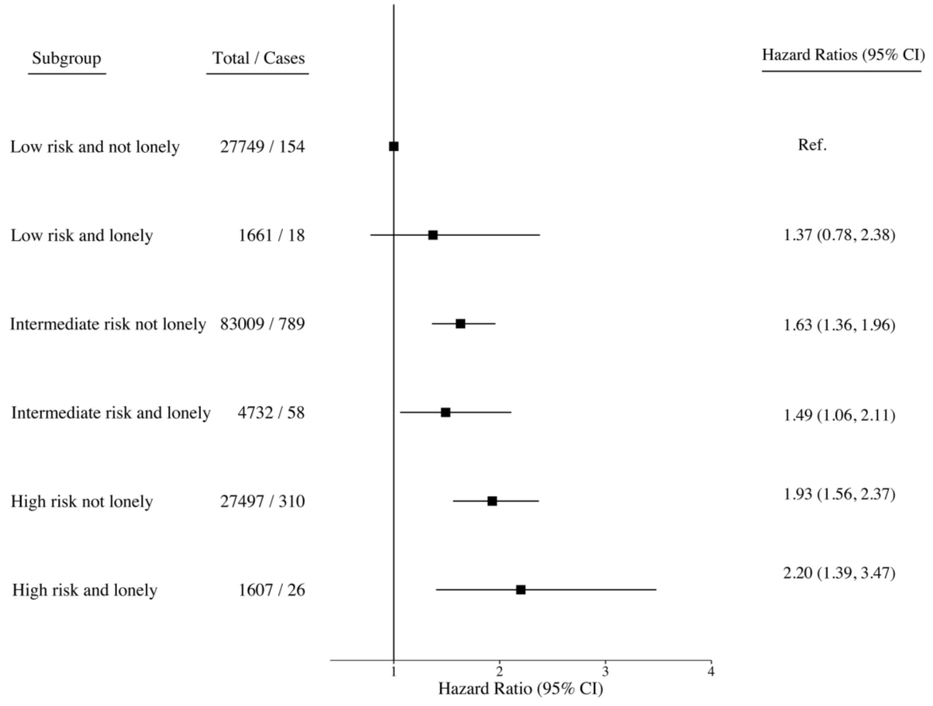

215x152mm (600 x 600 DPI)

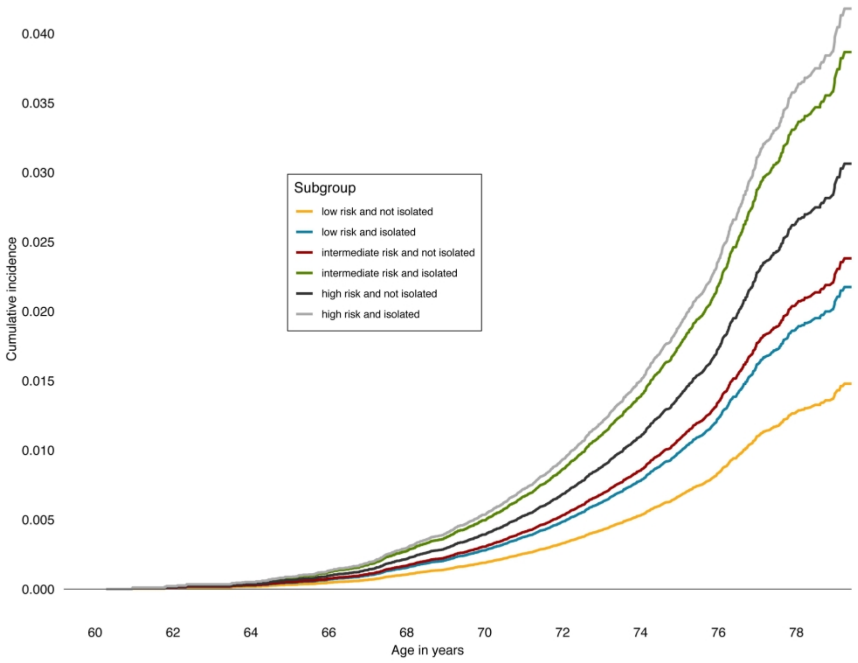

215x152mm (600 x 600 DPI)

Supplement: Elovainio, M, Lahti, J., Pirinen, M., Pulkki-Råback, L., Lipsanen, J., Virtanen, M., Kivimäki, M., & Hakulinen, C. The association between social isolation, loneliness, and genetic risk with incidence of dementia: UK Biobank cohort study

Table of content

1) Additional information of dementia assessment
2) Additional information of genetic risk score
3) The associations between genetic risk score and incident dementia using 10 various genetic risk score cut-off points
4) The associations of social isolation, loneliness and genetic risk score with specific Alzheimer's disease
5) The associations of combined social isolation/ loneliness and genetic risk score categories with incident dementia using imputed data
6) The sex stratified analyses of the final models

For peer review only

1) Additional information of dementia assessment

Incident all-cause dementia was defined using the following ICD-9 and ICD-10 codes:

ICD-9: 290.2, 290.3, 290.4, 291.2, 294.1, 331.0, 331.1, 331.2. 331.5

ICD-10: A81.0, F00, F00.0, F00.1, F00.2, F00.9, F01, F01.0, F01.1, F01.2, F01.3, F01.8, F01.9, F02, F02.0, F02.1, F02.2, F02.3, F02.4, F02.8, F03, F05.1, F10.6, G30, G30.0, G30.1, G30.8, G30.9, G31.0, G31.1, G31.8, I67.3

Incident Alzheimer's disease was defined using the following ICD-9 and ICD-10 codes:

ICD-9: 331.0

ICD-10: F00, F00.0, F00.1, F00.2, F00.9, G30, G30.0, G30.1, G30.8, G30.9

For more information of the dementia assessment see:
http://biobank.ndph.ox.ac.uk/showcase/showcase/docs/alg_outcome_dementia.pdf

For peer review only

2) Additional information of genetic risk score

International Genomics of Alzheimer's Project (IGAP) is a large three-stage study based upon genome-wide association studies (GWAS) on individuals of European ancestry. In stage 1, IGAP used genotyped and imputed data on 11,480,632 single nucleotide polymorphisms (SNPs) to meta-analyse GWAS datasets consisting of 21,982 Alzheimer's disease cases and 41,944 cognitively normal controls from four consortia: The Alzheimer Disease Genetics Consortium (ADGC); The European Alzheimer's disease Initiative (EADI); The Cohorts for Heart and Aging Research in Genomic Epidemiology Consortium (CHARGE); and The Genetic and Environmental Risk in AD Consortium Genetic and Environmental Risk in AD/Defining Genetic, Polygenic and Environmental Risk for Alzheimer's Disease Consortium (GERAD/PERADES). In stage 2, 11,632 SNPs were genotyped and tested for association in an independent set of 8,362 Alzheimer's disease cases and 10,483 controls. Meta-analysis of variants selected for analysis in stage 3A (n = 11,666) or stage 3B (n = 30,511) samples brought the final sample to 35,274 clinical and autopsy-documented Alzheimer's disease cases and 59,163 controls.

3) The associations between genetic risk score and incident dementia using 10 various geneic risk score cut-off points

The associations between continuous PRS and incident dementia with various cut-off points is reported in eFigure 1 below. The bars are negative log10 -transformed p-values of the PRS-dementia association.

For peer review only

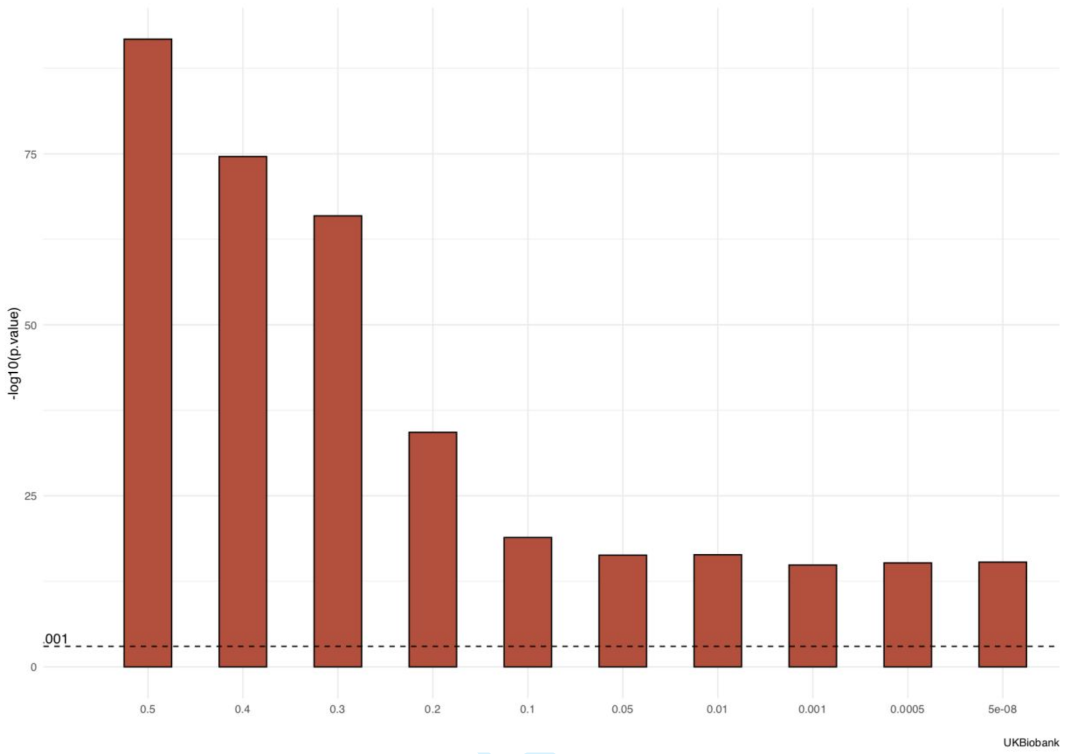

For peer review only

4) The associations of social isolation, loneliness and genetic risk score with specific Alzheimer's disease

We repeated all the analyses using specific Alzheimer's disease as the outcome instead of incident dementia and the results were materially the same, although there were, of course, much less Alzheimer's disease cases.

eTable 1. Risk of Incident Alzheimers' Disease According to Genetic Risk

	**Model 1**		**Model 2**	
Genetic risk	*HR 95 % CI*	*P-Value*	*HR 95 % CI*	*P-Value*
Intermediate	1.91 (1.45 – 2.51)	**<0.001**	1.84 (1.35 – 2.50)	**<0.001**
High	2.51 (1.86 – 3.38)	**<0.001**	2.43 (1.74 – 3.40)	**<0.001**
Observations	155074		132628	
R^2 Nagelkerke	0.345		0.392	

Model 1. Adjusted for age, sex, and 10 principal components
Model 2. Adjusted for age, sex, 10 principal components, education, social deprivation, depressive symptoms, health behaviors, loneliness and social isolation

For peer review only

eTable 2. Risk of Incident Azheimers' Disease According to Social Isolation and Loneliness Categories

	Model 1		Model 2	
Predictor	*Estimates*	*P-Value*	*Estimates*	*P-Value*
Isolated	1.56 (1.20 – 2.02)	**<0.001**	1.40 (1.05 – 1.88)	**0.024**
Observations	152723		137903	
R^2 Nagelkerke	0.288		0.395	
	**Model 1**		**Model 2**	
Lonely	1.16 (0.72 – 1.52)	0.811	0.81 (0.51 – 1.24)	0.344
Observations	147614		132628	
R^2 Nagelkerke	0.273		0.392	
	**Model 1**		**Model 2**	
Lonely	0.94 (0.64 – 1.40)	0.774	0.81 (0.51– 1.26)	0.345
Isolated	1.54 (1.18 – 2.02)	**0.002**	1.41 (1.04 – 1.91)	**0.025**
Observations	145663		132628	
R^2 Nagelkerke	0.322		0.392	

Model 1. Adjusted for age and sex
Model 2. Adjusted for age, sex, education, social deprivation, depressive symptoms, health behaviors, genetic risk score and 10 principal components

For peer review only

eFugure 2a. The associations (Hazard ratios and 95% Cis) of combined genetic risk and isolation categories with incident Alzheimer's disease.

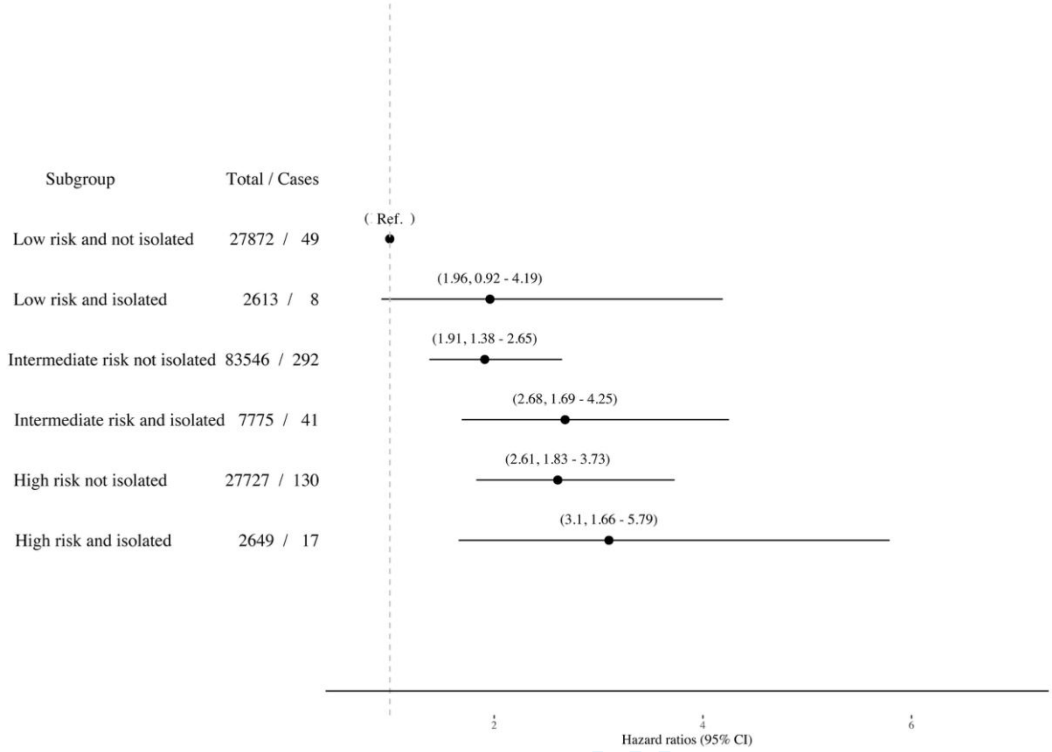

eFugure 2b. The associations (Hazard ratios and 95% Cis) of combined genetic risk and loneliness categories wih incident Alzheimer's disease.

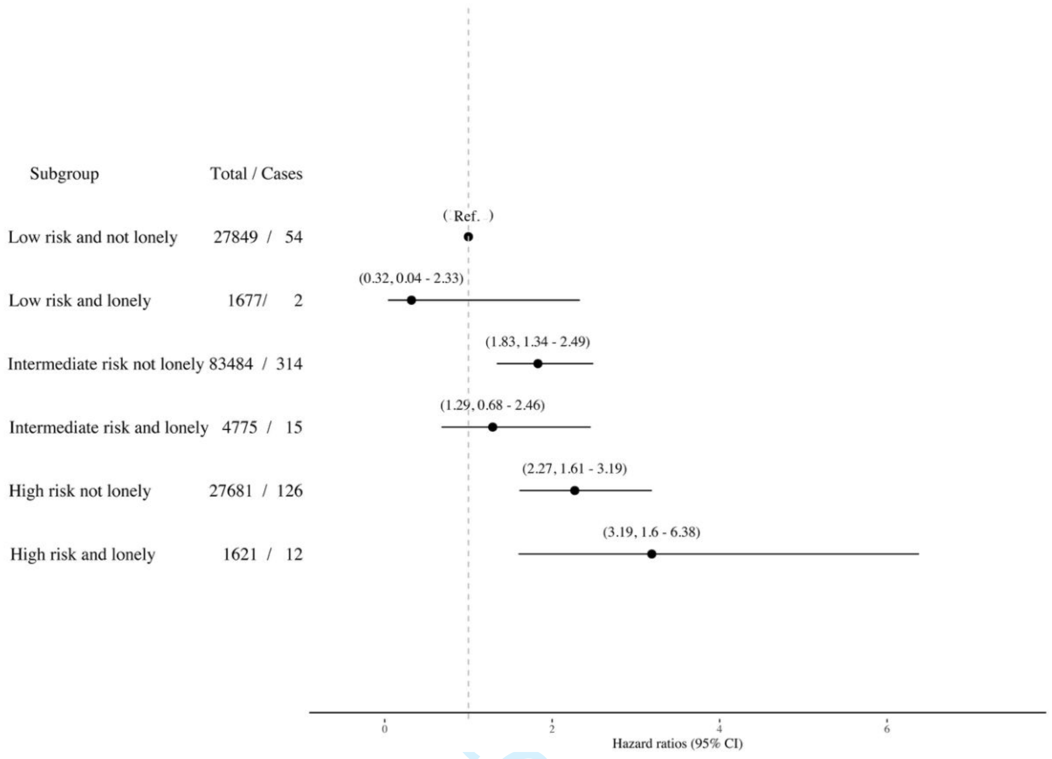

For peer review only

5) The associations of combined social isolation/ loneliness and genetic risk score categories with incident dementia using imputed data

The number of missing values was relatively small (only less the 5% had missing values), but we repeated the final models using five imputed data sets and, not surprisingly, the results were materially not changed (eFigure 3).

eFigure 3. The association (Hazard ratios and 95% Cis) of combined genetic risk and loneliness categories with incident dementia using imputed data (N = 155 070)

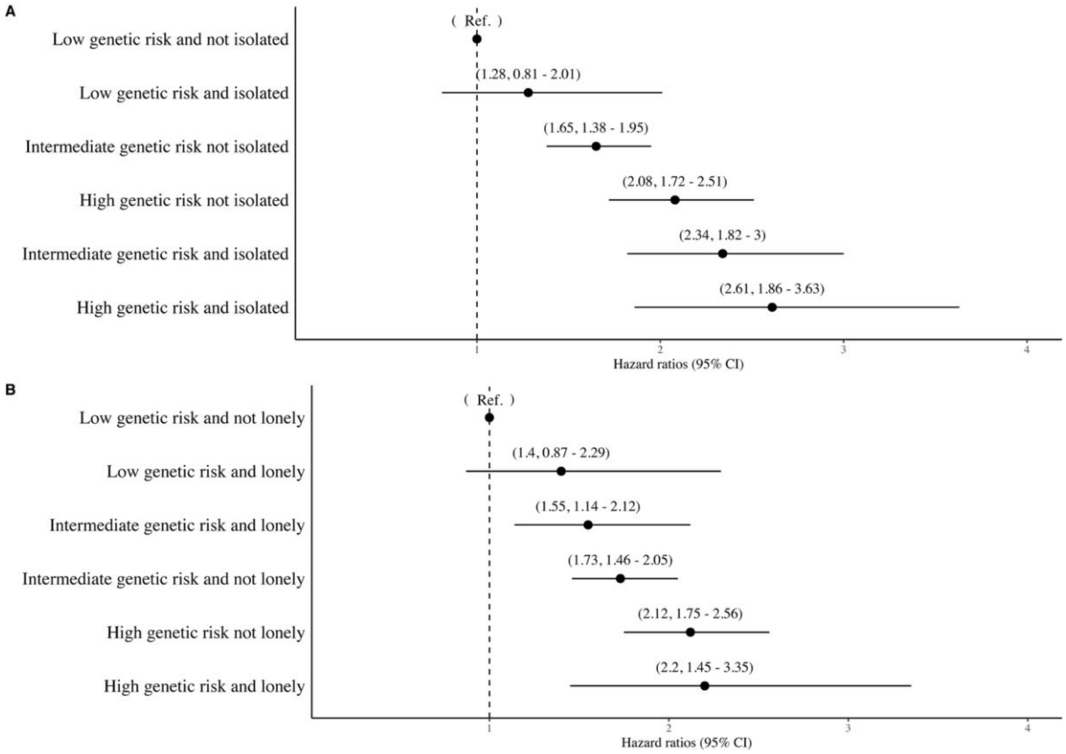

6) The sex stratified analyses of the final models

We stratified the data according sex and repeated the final analyses using these two data sets. There were only small

differences between men and women in any of the associations (eTable 3).

eTable 3. Sex stratified associations of combined genetic risk/ isolation and genetic risk / loneliness with incident dementia

**Women (N= 80 452)**					
**Group**	**No. of participants**	**Cases**	**HR**	**95% CI**	
Low risk and not isolated	14550	63	1.00	1.00	1.00
Low risk and isolated	1375	8	1.14	0.52	2.50
Intermediate risk not isolated	43284	332	1.66	1.24	2.22
Intermediate risk and isolated	3966	57	2.42	1.62	3.62
High risk not isolated	14108	127	1.96	1.41	2.72
High risk and isolated	1357	19	2.35	1.30	4.24
	**No. of participants**	**Cases**	**HR**	**95% CI**	
Low risk and not lonely	14548	64	1.00	1.00	1.00
Low risk and lonely	890	7	1.27	0.51	3.18
Intermediate risk not lonely	43040	356	1.72	1.30	2.29
Intermediate risk and lonely	2608	26	1.58	0.95	2.64
High risk not lonely	14033	133	1.92	1.39	2.66
High risk and lonely	866	10	2.14	1.05	4.36
**Men (N = 74618)**					
**Group**	**No. of participants**	**Cases**	**HR**	**95% CI**	
Low risk and not isolated	13218	90	1.00	1.00	1.00
Low risk and isolated	1225	13	1.64	0.91	2.96
Intermediate risk not isolated	39813	410	1.51	1.18	1.93
Intermediate risk and isolated	3738	55	1.95	1.35	2.82
High risk not isolated	13439	186	2.00	1.52	2.63
High risk and isolated	1267	23	2.42	1.47	3.98
**Group**	**No. of participants**	**Cases**	**HR**	**95% CI**	
Low risk and not lonely	13201	90	1.00	1.00	1.00
Low risk and lonely	771	11	1.43	0.71	2.87
Intermediate risk not lonely	39970	433	1.57	1.23	2.00
Intermediate risk and lonely	2124	32	1.42	0.89	2.27

	Women (N= 80 452)				
**Group**	**No. of participants**	**Cases**	**HR**	**95% CI**	
High risk not lonely	13466	177	1.92	1.46	2.52
High risk and lonely	742	16	2.23	1.23	4.04

For peer review only

STROBE (Strengthening The Reporting of OBservational Studies in Epidemiology) Checklist

A checklist of items that should be included in reports of observational studies. You must report the page number in your manuscript where you consider each of the items listed in this checklist. If you have not included this information, either revise your manuscript accordingly before submitting or note N/A.

Note: An Explanation and Elaboration article discusses each checklist item and gives methodological background and published examples of transparent reporting. The STROBE checklist is best used in conjunction with this article (freely available on the Web sites of PLoS Medicine at http://www.plosmedicine.org/, Annals of Internal Medicine at http://www.annals.org/, and Epidemiology at http://www.epidem.com/). Information on the STROBE Initiative is available at www.strobe-statement.org.

Section and Item	Item No.	Recommendation	Reported on Page No.
**Title and Abstract**	1	(*a*) Indicate the study's design with a commonly used term in the title or the abstract	2-3
		(*b*) Provide in the abstract an informative and balanced summary of what was done and what was found	2-3
**Introduction**			
Background/Rationale	2	Explain the scientific background and rationale for the investigation being reported	5
Objectives	3	State specific objectives, including any prespecified hypotheses	5
**Methods**			
Study Design	4	Present key elements of study design early in the paper	5-6
Setting	5	Describe the setting, locations, and relevant dates, including periods of recruitment, exposure, follow-up, and data collection	5-6
Participants	6	(*a*) *Cohort study*—Give the eligibility criteria, and the sources and methods of selection of participants. Describe methods of follow-up	6 -7
		Case-control study—Give the eligibility criteria, and the sources and methods of case ascertainment and control selection. Give the rationale for the choice of cases and controls	
		Cross-sectional study—Give the eligibility criteria, and the sources and methods of selection of participants	
		(*b*) *Cohort study*—For matched studies, give matching criteria and number of exposed and unexposed	
		Case-control study—For matched studies, give matching criteria and the number of controls per case	
Variables	7	Clearly define all outcomes, exposures, predictors, potential confounders, and effect modifiers. Give diagnostic criteria, if applicable	6-9

For peer review only

Section and Item	Item No.	Recommendation	Reported on Page No.
Data Sources/ Measurement	8*	For each variable of interest, give sources of data and details of methods of assessment (measurement). Describe comparability of assessment methods if there is more than one group	6-9
Bias	9	Describe any efforts to address potential sources of bias	9-10
Study Size	10	Explain how the study size was arrived at	6
Quantitative Variables	11	Explain how quantitative variables were handled in the analyses. If applicable, describe which groupings were chosen and why	6-9
Statistical Methods	12	(*a*) Describe all statistical methods, including those used to control for confounding	9
		(*b*) Describe any methods used to examine subgroups and interactions	9
		(*c*) Explain how missing data were addressed	9-10
		(*d*) *Cohort study*—If applicable, explain how loss to follow-up was addressed	9-10
		Case-control study—If applicable, explain how matching of cases and controls was addressed	
		Cross-sectional study—If applicable, describe analytical methods taking account of sampling strategy	
		(*e*) Describe any sensitivity analyses	9-10
**Results**			
Participants	13*	(a) Report numbers of individuals at each stage of study—eg numbers potentially eligible, examined for eligibility, confirmed eligible, included in the study, completing follow-up, and analysed	6
		(b) Give reasons for non-participation at each stage	6
		(c) Consider use of a flow diagram	
Descriptive Data	14*	(a) Give characteristics of study participants (eg demographic, clinical, social) and information on exposures and potential confounders	10
		(b) Indicate number of participants with missing data for each variable of interest	10
		(c) *Cohort study*—Summarise follow-up time (eg, average and total amount)	10
Outcome Data	15*	*Cohort study*—Report numbers of outcome events or summary measures over time	10 -11
		Case-control study—Report numbers in each exposure category, or summary measures of exposure	
		Cross-sectional study—Report numbers of outcome events or summary measures	

For peer review only

Section and Item	Item No.	Recommendation	Reported on Page No.
Main Results	16	(*a*) Give unadjusted estimates and, if applicable, confounder-adjusted estimates and their precision (eg, 95% confidence interval). Make clear which confounders were adjusted for and why they were included	10-11
		(*b*) Report category boundaries when continuous variables were categorized	6-9
		(*c*) If relevant, consider translating estimates of relative risk into absolute risk for a meaningful time period	11
Other Analyses	17	Report other analyses done—eg analyses of subgroups and interactions, and sensitivity analyses	12
**Discussion**			
Key Results	18	Summarise key results with reference to study objectives	12
Limitations	19	Discuss limitations of the study, taking into account sources of potential bias or imprecision. Discuss both direction and magnitude of any potential bias	14
Interpretation	20	Give a cautious overall interpretation of results considering objectives, limitations, multiplicity of analyses, results from similar studies, and other relevant evidence	12 -15
Generalisability	21	Discuss the generalisability (external validity) of the study results	14
**Other Information**			
Funding	22	Give the source of funding and the role of the funders for the present study and, if applicable, for the original study on which the present article is based	16-17

*Give information separately for cases and controls in case-control studies and, if applicable, for exposed and unexposed groups in cohort and cross-sectional studies.

Once you have completed this checklist, please save a copy and upload it as part of your submission. DO NOT include this checklist as part of the main manuscript document. It must be uploaded as a separate file.

For peer review only

BMJ Open

Association of social isolation, loneliness, and genetic risk with incidence of dementia: UK Biobank cohort study

Journal:	*BMJ Open*
Manuscript ID	bmjopen-2021-053936.R1
Article Type:	Original research
Date Submitted by the Author:	30-Dec-2021

Complete List of Authors:	Elovainio, Marko; University of Helsinki; Finnish Institute for Health and Welfare<br>Lahti, Jari; University of Helsinki<br>Pirinen, Matti; University of Helsinki; University of Helsinki, Department of Public Health<br>Pulkki-Raback, Laura; University of Helsinki, Psychology and logopedics<br>Malmberg, Anni; University of Helsinki<br>Lipsanen, Jari; University of Helsinki, Psychology and logopedics<br>Virtanen, Marianna; Itä-Suomen yliopisto, School of Educational Sciences and Psychology<br>Kivimaki, Mika; University College London, Department of Epidemiology & Public Health<br>Hakulinen, Christian; University of Helsinki, Psychology and logopedics

**Primary Subject Heading**:	Epidemiology
Secondary Subject Heading:	Neurology, Public health
Keywords:	Dementia < NEUROLOGY, Public health < INFECTIOUS DISEASES, GENETICS, GERIATRIC MEDICINE

SCHOLARONE™
Manuscripts

I, the Submitting Author has the right to grant and does grant on behalf of all authors of the Work (as defined in the below author licence), an exclusive licence and/or a non-exclusive licence for contributions from authors who are: i) UK Crown employees; ii) where BMJ has agreed a CC-BY licence shall apply, and/or iii) in accordance with the terms applicable for US Federal Government officers or employees acting as part of their official duties; on a worldwide, perpetual, irrevocable, royalty-free basis to BMJ Publishing Group Ltd ("BMJ") its licensees and where the relevant Journal is co-owned by BMJ to the co-owners of the Journal, to publish the Work in this journal and any other BMJ products and to exploit all rights, as set out in our licence.

The Submitting Author accepts and understands that any supply made under these terms is made by BMJ to the Submitting Author unless you are acting as an employee on behalf of your employer or a postgraduate student of an affiliated institution which is paying any applicable article publishing charge ("APC") for Open Access articles. Where the Submitting Author wishes to make the Work available on an Open Access basis (and intends to pay the relevant APC), the terms of reuse of such Open Access shall be governed by a Creative Commons licence – details of these licences and which Creative Commons licence will apply to this Work are set out in our licence referred to above.

Other than as permitted in any relevant BMJ Author's Self Archiving Policies, I confirm this Work has not been accepted for publication elsewhere, is not being considered for publication elsewhere and does not duplicate material already published. I confirm all authors consent to publication of this Work and authorise the granting of this licence.

For peer review only

21 December 2021

Association of social isolation, loneliness, and genetic risk with incidence of dementia: UK Biobank cohort study

Marko Elovainio, PhD[1,2]
Jari Lahti, PhD[1]
Matti Pirinen, PhD[3,4,5]
Laura Pulkki-Råback, PhD [1,6]
Anni Malmberg, BA[1]
Jari Lipsanen, MSci [1]
Marianna Virtanen, PhD [7]
Mika Kivimäki, FMedSci [4,8]
Christian Hakulinen, PhD [1]

[1]Medical Research Program Unit, Faculty of Medicine, University of Helsinki, Finland;
[2]Finnish Institute for Health and Welfare, Finland;
[3]Institute for Molecular Medicine Finland, Helsinki Institute of Life Sciences, University of Helsinki;
[4]Department of Public Health, University of Helsinki, Finland;
[5]Helsinki Institute for Information Technology and Department of Mathematics and Statistics, University of Helsinki, Finland;
[6]Research Centre of Child Psychiatry, Faculty of Medicine, University of Turku, Finland
[7]School of Educational Sciences and Psychology, University of Eastern Finland, Joensuu, Finland;
[8]Department of Epidemiology and Public Health, University College London, London, UK;

Correspondence: Marko Elovainio, University of Helsinki, P.O. Box 9, 00014, Helsinki, Finland, Phone: +358 50 3020621, email: marko.elovainio@helsinki.fi
Word count for abstract / main text: 200 / 3259; Number of tables and figures: 4 +2

Keywords: Social network, social support, dementia, cognitive decline, public health

For peer review only

Background: Social isolation and loneliness have been associated with increased risk of dementia, but it is not known whether this risk is modified or confounded by genetic risk of dementia.

Methods: We used the prospective UK Biobank study with 155 070 participants (mean age 64.1 years), including self-reported social isolation and loneliness. Genetic risk was indicated using the polygenic risk score for Alzheimer's disease and the incident dementia ascertained using electronic health records.

Results: Overall, 8.6% of participants reported that they were socially isolated and 5.5% were lonely. During a mean follow-up of 8.8 years (1.36 million person-years), 1444 (0.9% of the total sample) were diagnosed with dementia. Social isolation, but not loneliness, was associated with increased risk of dementia (hazard ratio 1.62, 95% confidence interval 1.38 to 1.90). There were no interaction effects between genetic risk and social isolation or between genetic risk and loneliness predicting incident dementia. Of the participants who were socially isolated and had high genetic risk,4.4% (3.4% to 5.5%) were estimated to developed dementia compared with 2.9% (2.6% to 3.2%) of those who were not socially isolated but had high genetic risk. Comparable differences were also in those with intermediate and low genetic risk levels.

Conclusions: Socially isolated individuals are at increased risk of dementia at all levels of genetic risk.

For peer review only

What is already known on this topic

- Social isolation and loneliness have been associated with increased risk of dementia
- It is not known whether this risk is modified or confounded by genetic risk of dementia

What this study adds

- This is the first study to show that social isolation is associated with increased risk of dementia across the spectrum of genetic risk.
- Loneliness, in contrast to social isolation, seems to be less consistently associated with dementia when combined with genetic risk.

Article summary

- We showed that socially isolated individuals have higher risk for dementia across the spectrum of genetic risk.
- This study suggests that social isolation is a risk factor of its own, over and above genetic risk.

For peer review only

Strengths and limitations of the study

- The strengths of the study were its large sample size and a genome-wide study using a well-established polygenic risk score for dementia.
- Despite the large sample size, the sample was not representative of the UK population.
- As dementia was derived from hospital records, people with non-diagnosed dementia may have been missed.
- Reverse causation may have affected the findings by making people with pre-clinical dementia more socially isolated.
- Future research should examine the mechanistic pathways whereby social isolation is associated with dementia.

For peer review only

The rapidly rising numbers of people with dementia [1] is a significant health policy and health service concern in many high-income countries. Although considerable share of the dementia risk is due to genetic factors [2, 3], major efforts have been directed towards the identification of potentially modifiable risk factors that could prevent or delay the onset of dementia [4]. Higher levels of social support have been suggested to protect from dementia [5], with both social isolation and feelings of loneliness being associated with increased risk of dementia [6-8], although mixed findings have been reported between loneliness and dementia risk [9, 10]. However, it remains unclear whether there is an interplay between genetic factors and social isolation and loneliness (*i.e.* whether the association of social isolation and loneliness with dementia is evident only at high or low levels of genetic risk) or whether the associations of genetic factors and social network characteristics with dementia are independent and additive.

The polygenic risk score (PRS) for Alzheimer's disease, describing the polygenic burden captured by the most recent genome-wide studies [11], allows to estimate the size of the genetic risk and the extent to which the associations of social isolation and loneliness with dementia are modified by genetic risk. Existing studies have included APOE genotype as the genetic risk, focused on wider psychosocial characteristics [12], relied on small samples [13], and provided limited evidence for the interplay of genetic risk and social relations predicting the increased risk of incident dementia. In the present study, we used data from UK Biobank study to examine whether genetic risk may intensify and attenuate the associations of social isolation and loneliness with the risk of dementia. In addition to estimating relative risk, we will provide estimates of absolute risk [14], as they are important information for risk communication and clinical risk prediction [15].

METHODS

Study design and participants

For peer review only

In this analysis of the UK Biobank study, we used baseline data and obtained information of incident dementia at follow-up via linked electronic health records [16]. UK National Health Service (NHS) registers maintain records of all individuals legally registered as residents in the United Kingdom. In the UK Biobank study, these records were used to invite around 9.2 million individuals aged 40–69 years living within a sensible travelling distance of the 22 assessment centres across Great Britain 2007–2010 [16]. At the study baseline, participants completed multiple touchscreen computer-based questionnaires followed by a face-to-face interview with trained research staff. Physical measures were also taken. Details of these assessments and variables are publicly available from the UK Biobank website: http://biobank.ctsu.ox.ac.uk/crystal/.

In total, 502,656 individuals were recruited (5.4% of the eligible population). Of those, individuals that were 60 year or older and had complete data on social isolation, loneliness, dementia and genetic data were included in the present analysis (N = 147 614 – 152 070). There were 7459 (4.8%) missing values in loneliness measures and 2351 (1.5%) missing values in isolation measures. We also repeated the analyses using imputed data in those with missing information on social isolation, loneliness or other explanatory variables but had information on genetic risk score (N = 155 063). This study was conducted under generic approval from the NHS National Research Ethics Service (17th June 2011, Ref 11/NW/0382). Participants provided electronic consent for the baseline assessments and register linkage.

Ascertainment of incident dementia

Dementia was ascertained using hospital inpatient records which contains data on admissions and diagnoses from the Hospital Episode Statistics for England, Scottish Morbidity Record data for Scotland, and the Patient Episode Database for Wales. Additional cases were detected through linkage to death register data provided by the National Health Service Digital for England and Wales and the Information and Statistics Division for Scotland. Diagnoses were recorded using the International Classification of Diseases (ICD) coding system. Participants with dementia were

For peer review only

identified as having a primary/secondary diagnosis (hospital records) or underlying/contributory cause of death (death register) using ICD-9 and ICD-10 codes for Alzheimer disease and other dementia classifications (see supplement for details).

Measurement of social isolation and loneliness

Social isolation and loneliness were measured using the same scale as in our two previous UK Biobank studies [17, 18]. *Social isolation* scale was defined using the following three questions: (a) “Including yourself, how many people are living together in your household? Include those who usually live in the house such as students living away from home during term, partners in the armed forces or professions such as pilots” (1 point for living alone) (b) “How often do you visit friends or family or have them visit you?” (1 point for friends and family visits less than once a month), and (c) “Which of the following [leisure/social activities] do you attend once a week or more often? You can select more than one", (1 point for no participation in social activities at least weekly). This resulted in scale with a range from 0 to 3, where an individual was defined as socially isolated if he/she had two or more of those points and those who scored 0 or 1 were classified as not isolated. Other studies in the UK have used similar measures [18].

Loneliness scale was constructed from two questions: "Do you often feel lonely? " (no = 0, yes=1) and “"How often are you able to confide in someone close to you?"(0 = almost daily-once every few months 1= once every few months to never or almost never). An individual was defined as lonely if he/she responded positively to both questions (score 2) and not lonely if he or she responded negatively to one or both of the questions (score 0 -1). Similar questions have been used in longer loneliness scales, such as the Revised UCLA Loneliness Scale [19].

Polygenic risk score of dementia

For peer review only

From the genotyped UK Biobank samples, we included 155,070 unrelated white British participants after removal of participants based on heterozygosity and missingness of outliers, sex chromosome aneuploidies and mismatches, withdrawals, and those that UK Biobank had excluded from the relatedness calculations. The genotypes were imputed against Haplotype Reference Consortium and UK10K haplotype resources containing ~96M variants [11]. We calculated polygenic risk scores (PRS) for Alzheimer's disease (AD) based on a genome-wide association study by Kunkle and others (2019) with 35,274 AD cases and 59,163 controls that do not overlap with UK Biobank samples (for details see the online supplement). We used Plink 1.9 [20] for the genotype QC and clumping. The following parameters were used for the clumping of the genotype data: p-value threshold 0.5, LD threshold (r^2) 0.5, and clumping window width of 250 kilobases. Prior to clumping we excluded all SNPs with MAF < 0.001, genotyping rate < 0.1, Hardy-Weinberg equilibrium p-value < 1e-6 and missingness per person >0.1. We used PRSice 2.2.8 [21] for calculating the PRS with the genotype QC settings that have been recommended by the software developers [22]. In the main analyses, we applied a p-value threshold of 0.5, which resulted in including 626,623 SNPs in the PRS. This threshold was chosen as previous work has reported that it provided an optimal set of variants for predicting dementia and AD [23, 24]. While this set is likely to include a number of variants which are not associated with AD, it also includes a number of variants that at present do not have sufficient statistical evidence to meet the criteria for being genome-wide significant (i.e. P-value < 5x10-8) but are expected to be associated in future larger studies. The univariate associations between genetic risks score with 10 different cut-off points and incident dementia is reported in the supplement (SFigure 1). Last, based on two single nucleotide polymorphisms (rs7412 and rs429358), we additionally genotyped APOE (none, one, or, two ε4 alleles).

The polygenic risk scores were then z-standardized to have mean 0 and variance 1, and divided into tertiles and categorized as low-, intermediate- and high-risk tertiles.

Assessment of potential explanatory factors

Following information was used in the current study: sex, age in years, socioeconomic factors (educational attainment and Townsend deprivation index, which is an area-level composite measure of deprivation based on unemployment, non-home ownership, non-car ownership, and household overcrowding), chronic diseases (diabetes, cardiovascular disease, cancer, and other long-standing illness, disability or infirmity), cigarette smoking (smoker [yes/no]; ex-smoker[yes/no]), physical activity (moderate and vigorous physical activity), alcohol intake frequency (Three or four times a week or more vs. once or twice a week or less), and the frequency of depressed mood in the past 2 weeks (Patient Health Questionnaire; [25]).

Statistical analyses

Study participants were followed from the study baseline (2006-2010) for incident dementia until the date of first dementia diagnosis, death, or to the end of the follow-up, whichever came first. The associations of social isolation, loneliness and polygenic risk score with incident dementia were examined using Cox proportional hazard regression models where age was used as a time scale. Results from these analyses were reported as hazard ratios (relative risk) and their 95% confidence intervals and the models were adjusted for age, sex, and 10 first principal components of genetic structure from UK Biobank to control for possible population stratification, and additionally for education, social deprivation index, having long term illness, physical activity, smoking status, alcohol consumption, and depressive symptoms. In these analyses, PRS was used both as a categorical and as a continuous variable. Additional adjustments were also made for APOE genotype. Cumulative incidence (absolute risk) of dementia associated with combined categories of

For peer review only

social isolation, loneliness and genetic risk was estimated using competing-risks regression [26, 27], with death being treated as competing event.

For the sensitivity analyses, missing data on social isolation, loneliness and all explanatory factors were imputed using multiple imputation by chained equations to generate five imputed datasets. Imputation model included age, sex, social isolation, loneliness, all covariates, the Nelson-Aalen estimate of cumulative hazard, and survival status [28]. Cox proportional hazards models were fitted within each imputed dataset and combined using Rubin's rules.

P-values were 2-sided with statistical significance set at less than .05. All analyses were performed using Stata (15.1) and R (4.2.1).

Role of the funding source

The sponsors of the study had no role in study design, data collection, data analysis, data interpretation, or writing of the report. Elovainio and Hakulinen had full access to the data. Elovainio and Hakulinen take final responsibility for the decision to submit for publication.

Patient involvement

These results are based on existing data. We were not involved in the recruitment of the participants. As far as we know, no patients were engaged in designing the present research question or the outcome measures. They were also not involved in developing plans for recruitment, design, or implementation of the study, and were not asked to advise on interpretation or writing up of results. Results from UK Biobank are disseminated to study participants via the study website and social media outlets.

RESULTS

Descriptive statistics of the study participants are shown in **Table 1**. Genetic risk score data were available for 155 070 participants (51.9% women; mean age 64.1 years). Overall, 8.6% of participants (N = 13103) were classified as socially isolated and 5.5% were lonely (N = 8102). Of those who reported themselves to be socially isolated 14.3.% were also lonely. During a total of

For peer review only

1.36 million person-years (mean follow-up time 8.8 years), 1444 participants (0.9% of the total sample) were diagnosed with all-cause dementia.

As expected, a higher PRS for AD was associated with an increased risk of dementia. Using continuous PRS, the hazard ratio per 1SD increase in the score was 1.27 (95% CI 1.21 to 1.34) in an analysis adjusted for age, sex and 10 principal components. The associations between genetic risk categories (low, intermediate, and high) with incidence of dementia shown in **Table 2**. In comparison to the participants in the low genetic risk category, the hazard ratio of incident dementia was 1.49 (95% CI 1.28 – 1.73) in participants with intermediate risk and 1.71 (95% CI 1.47 – 1.98) in those with high genetic risk in the fully adjusted model. There were no interaction effects between sex and intermediate genetic risk (p = 0.15) or between sex and high genetic risk (p = 0.20) predicting incident dementia (Stable 1a).

Social isolation was associated with increased risk of dementia (HR adjusted for age and sex = 1.62, 95% CI 1.39 – 1.90). The associations attenuated but remained statistically significant after adjusting for additional covariates including socio-demographics, health-related factors and genetic risk score and principal components (HR = 1.34, 95% CI 1.12 – 1.60). Loneliness was also associated with higher risk of dementia (HR = 1.47, 95% CI 1.20 – 1.80), but this association was lost when adjusted for socio-demographics, health-related factors, PRS and principal components (HR = 1.03, 95% CI 0.81 – 1.30). Both social isolation (HR = 1.58, 95% CI 1.34 – 1.86) and loneliness (HR = 1.28, 95% CI 1.03 – 1.59) were associated with incident dementia when added simultaneously into the model but only the association between social isolation and dementia was robust to adjusting for additional covariates (HR = 1.33, 95 % CI 1.11 – 1.60). Adjusting the models for APOE produced similar associations (**Table 3**). No interaction effects between sex and isolation (p = 0.53) or between sex and loneliness (p = 0.14) predicting incident dementia were found (Stable 1b).

For peer review only

Although no significant interaction effects in the associations between social isolation and genetic risk categories (p-values range 0.45-0.62) or loneliness and genetic risk categories (p-values range 0.59-0.95) with incident dementia were found (Stable 1c), we illustrated the interplay between genetic risk with social isolation and loneliness by presenting associations at all genetic risk levels adjusting for potential confounders (Figures 1 and 2). Social isolation was associated with increasing dementia risk in all genetic risk levels. At intermediate and high genetic risk levels, these associations were robust to adjusting for all potential confounders or mediators (hazard ratio = 1.37, 95% CI, 1.01-1.86; hazard ratio= 1.38, 95% CI, 1.04-1.82). The results for loneliness were less consistent, and the risk of dementia was similar in lonely participants at low and at high levels of genetic risk, when compared with those who reported no loneliness. In the high genetic risk group, for example, the hazard ratios were 1.53 (95% CI 1.11 – 2.09) in low and 1.56 (95% CI 1.04 – 2.35) in high loneliness group (**Figure 2**). All these association were attenuated when adjusted for long-term illness and depressive symptoms and in the fully adjusted model.

In terms of absolute risk (cumulative incidence), of those who were socially isolated and had high genetic risk, 4.4% (3.4% to 5.5%) were estimated to developed dementia compared with 2.9% (2.6% to 3.2%) of those who were not socially isolated but had high genetic risk (**Figure 3**). The corresponding absolute risk estimates in the socially isolated and not isolated were 4.1 (3.1% to 5.1%) and 2.5% (2.2% to 2.8%) in participants with intermediate genetic risk and 2.3% (1.5% to 3.0%) and 1.6% (1.4% to 1.9%) in those with low genetic risk.

As sensitivity analyses, we repeated all the main analyses with Alzheimer's disease as the outcome and using imputed data sets (Supplement SFigures 2 – 4). The results did not materially change. To detect whether the associations were due to reverse causation, we additionally repeated the fully adjusted models using data where those dementia cases occurring in the first three years of the follow-up were excluded. The association between isolation and incident

For peer review only

dementia (hazard ratio= 1.30, 95% CI, 1.08-1.58) and between loneliness and incident dementia (hazard ratio= 1.06, 95% CI, 0.82-1.36) were basically the same.

DISCUSSION

In this UK Biobank study of 155 063men and women, social isolation was associated with increased risk of all-cause dementia and Alzheimer's disease at intermediate and high levels of genetic risk of Alzheimer's disease. No interaction effects were found between genetic risk levels and isolation predicting incident dementia. The incidence of dementia was estimated to reach over 4% in isolated high-genetic risk individuals compared to approximately 3% in non-isolated individuals with similar genetic risk. The difference between these groups was comparable also among those with intermediate and low genetic risk. This means that among individuals with similar genetic risk for dementia, those who are socially isolated are more likely to have incidence of the disease, suggesting an effect by social isolation over and above that of genetic risk. The association between loneliness and dementia was attributable to other dementia risk factors, such as health behaviours and depressive symptoms.

The relative risk of dementia across the genetic risk categories was at the same magnitude as in a previous UK Biobank study [29] that used data from an older GWAS [30]. Our findings also support other studies - most of which with follow-ups from 5 to 11 years – showing an association of social isolation with increased risk of dementia [6, 8, 10]. A 28-year follow-up of 10,000 Whitehall II study participants found that less frequent social contacts at ages 50, 60 and 70 were associated with approximately 10% higher dementia risk, independent of socio-economic and other lifestyle factors [31]. While previous studies have produced mixed findings on whether loneliness is associated with increased risk of dementia or not [9, 10], our findings show that the association between loneliness and dementia is mostly likely explained by other factors and present only at high levels of genetic risk.

For peer review only

Our results should be interpreted in a context of disease aetiology. Dementia is characterised by a 10-20-year preclinical or prodromal stage during which changes in biomarkers and cognitive abilities increasingly occur [32]. With a follow-up less than 10 years, it is likely that we assessed social isolation for dementia cases during this preclinical period. This could result to reverse causality, i.e., increased prevalence of social isolation during the 8-year period could have resulted from preclinical changes in social activity leading to a spurious association between social isolation and dementia.

Several mechanisms through which social isolation may causally affect dementia risk have been proposed. Social isolation and loneliness have been suggested to increase stress reactivity which is associated with prolonged activation of the hypothalamic-pituitary-adrenal axis (HPA) and the sympatho-adrenal system [33]. This process may further lead to sleep deprivation, dysregulation of the immune system, and even increased levels of oxidative stress [34], all potentially harmful for cognitive health. It has also been shown that socially isolated and lonely individuals more often engage in health-damaging behaviors [18], which may affect cognition either directly via biophysiological mechanisms or increased incidence of cardiometabolic diseases which accelerate neurodegeneration [35]. Socially isolated or lonely individuals are also at an increased risk of depression [36], a potential risk factor for cognitive decline and dementia [37]. Participation in social activities and social interaction stimulates neural plasticity by building and maintaining cognitive reserve [38]. Poor cognitive reserve is a further pathway through which social isolation and loneliness could increase dementia risk [39]. Fewer social contacts with reduced exercising of memory and language adversely affect cognitive reserve, thereby accelerating dementia onset [39]. Cognitive ability was not assessed in the present study and a small share of the found association between social isolation and subsequent dementia risk may be attributable to lower initial cognitive reserve.

Strengths and limitations

For peer review only

The major strengths of the current study include the large sample size of UK Biobank participants, which enabled us to study the combination of genetic risk, social isolation, and loneliness in detail. In addition, we used the largest genome-wide association study of dementia to date to derive the genetic risk for AD [2].

There are also some important limitations. Although our analyses were adjusted for multiple potential sources of bias, the possibility of unmeasured confounding and reverse causation cannot be ruled out. However, the results were basically unchanged when excluding those with incident dementia during the first three -year follow-up time. Both frequency of social contacts and loneliness were self-reported and measured by relatively short and crude measures. As we were able to cover the genetic risk for AD – not all-cause dementias – based on the Kunkle et al [2], we may have missed some of the genetic variance related to non-AD dementias. Dementia cases were derived from medical records or death registers, and thus some cases might have been missed. However, good agreement of dementia case determination with primary care record data has been shown [40]. This sample was restricted to volunteers of European ancestry aged 60 to 73 years at baseline and, therefore, further research is needed to ensure generalizability of our findings. As the mean age of participants was only 72 years at the end of the follow-up period, the incidence of dementia remained low. As noted previously the response rate of the UK Biobank study survey was very low, 5.5%, and UK Biobank is not representative of the sampling population [41]. However,

For peer review only

many etiological findings from UK Biobank appear to be generalisable to England and Scotland [42].

Conclusions

The present findings suggest an association between social isolation and increased risk of dementia across the spectrum of genetic risk. Further research is needed to determine the extent social isolation is a modifiable risk factor rather than a part of the dementia prodrome

For peer review only

Acknowledgments: We thank the International Genomics of Alzheimer's Project (IGAP) for providing summary results data for these analyses. The investigators within IGAP contributed to the design and implementation of IGAP and/or provided data but did not participate in analysis or writing of this report. IGAP was made possible by the generous participation of the control subjects, the patients, and their families. The i–Select chips was funded by the French National Foundation on Alzheimer's disease and related disorders. EADI was supported by the LABEX (laboratory of excellence program investment for the future) DISTALZ grant, Inserm, Institut Pasteur de Lille, Université de Lille 2 and the Lille University Hospital. GERAD/PERADES was supported by the Medical Research Council (Grant n° 503480), Alzheimer's Research UK (Grant n° 503176), the Wellcome Trust (Grant n° 082604/2/07/Z) and German Federal Ministry of Education and Research (BMBF): Competence Network Dementia (CND) grant n° 01GI0102, 01GI0711, 01GI0420. CHARGE was partly supported by the NIH/NIA grant R01 AG033193 and the NIA AG081220 and AGES contract N01–AG–12100, the NHLBI grant R01 HL105756, the Icelandic Heart Association, and the Erasmus Medical Center and Erasmus University. ADGC was supported by the NIH/NIA grants: U01 AG032984, U24 AG021886, U01 AG016976, and the Alzheimer's Association grant ADGC–10–196728.

Contributors: ME and CH designed the study and conducted the statistical analyses. ME wrote the first draft of the manuscript. JL and AM calculated the polygenetic risk score with the help of MP. All authors (ME, JL, MP, LPR, AM, JL, MV, MK, CH) contributed to the interpretation of the results and critical revision of the manuscript for important intellectual content and approved the final version of the manuscript. The corresponding author attests that all listed authors meet authorship criteria and that no others meeting the criteria have been omitted.

For peer review only

Funding: ME and CH were supported by the Academy of Finland (339390 (ME) / 310591(CH)).

MK was supported by NordForsk (70521), the UK Medical Research Council (MRC S011676), the Academy of Finland (311492), and the US National Institutes on Ageing (NIA R01AG056477). . Laura Pulkki-Råback was supported by the Jenny and Antti Wihuri Foundation. GERAD/PERADES was supported by the Medical Research Council (Grant n° 503480), Alzheimer's Research UK (Grant n° 503176), the Wellcome Trust (Grant n° 082604/2/07/Z) and German Federal Ministry of Education and Research (BMBF): Competence Network Dementia (CND) grant n° 01GI0102, 01GI0711, 01GI0420. CHARGE was partly supported by the NIH/NIA grant R01 AG033193 and the NIA AG081220 and AGES contract N01–AG–12100, the NHLBI grant R01 HL105756, the Icelandic Heart Association, and the Erasmus Medical Center and Erasmus University. ADGC was supported by the NIH/NIA grants: U01 AG032984, U24 AG021886, U01 AG016976, and the Alzheimer's Association grant ADGC–10–196728. The funding sources did not participate in the design or conduct of the study; collection, management, analysis or interpretation of the data; or preparation, review, or approval of the manuscript.

Competing interests: All authors have completed the ICMJE uniform disclosure form at www.icmje.org/coi_disclosure.pdf (available on request from the corresponding author) and declare: no support from any organisation for the submitted work, no other relationships or activities that could appear to have influenced the submitted work.

Ethical approval: Ethical approval for data collection was given by the North-West Multi-centre Research Ethics Committee. Study was carried out in accordance with the Declaration of Helsinki

For peer review only

of the World Medical Association. The ethical board of the Finnish Institute for Health and Welfare gave ethical permission to use the genetic data.

Data sharing: The genetic and phenotypic UK Biobank data are available on application to the UK Biobank (www.ukbiobank.ac.uk/). Present study was conducted using the UK Biobank Resource under Application 14801. Summary statistics from the meta-analysis of genome wide association studies in dementia are available from https://www.niagads.org/datasets/ng00075

Transparency statement: The lead authors (ME and CH) affirms that the manuscript is an honest, accurate, and transparent account of the study being reported; that no important aspects of the study have been omitted; and that any discrepancies from the study as planned (and, if relevant, registered) have been explained. Preprint: https://www.medrxiv.org/content/10.1101/2020.02.25.20027177v1

For peer review only

References

1 Ahmadi-Abhari S, Guzman-Castillo M, Bandosz P*, et al.* Temporal trend in dementia incidence since 2002 and projections for prevalence in England and Wales to 2040: modelling study. *BMJ* 2017;**358**:j2856.

2 Kunkle BW, Grenier-Boley B, Sims R*, et al.* Genetic meta-analysis of diagnosed Alzheimer's disease identifies new risk loci and implicates Abeta, tau, immunity and lipid processing. *Nat Genet* 2019;**51**:414-30.

3 Andrews SJ, Fulton-Howard B, Goate A. Interpretation of risk loci from genome-wide association studies of Alzheimer's disease. *Lancet Neurol* 2020.

4 Soto-Gordoa M, Arrospide A, Moreno-Izco F*, et al.* Projecting Burden of Dementia in Spain, 2010-2050: Impact of Modifying Risk Factors. *J Alzheimers Dis* 2015;**48**:721-30.

5 Livingston G, Sommerlad A, Orgeta V*, et al.* Dementia prevention, intervention, and care. *Lancet* 2017;**390**:2673-734.

6 Kuiper JS, Zuidersma M, Oude Voshaar RC*, et al.* Social relationships and risk of dementia: A systematic review and meta-analysis of longitudinal cohort studies. *Ageing Res Rev* 2015;**22**:39-57.

7 Lara E, Caballero FF, Rico-Uribe LA*, et al.* Are loneliness and social isolation associated with cognitive decline? *Int J Geriatr Psychiatry* 2019.

8 Lara E, Martin-Maria N, De la Torre-Luque A*, et al.* Does loneliness contribute to mild cognitive impairment and dementia? A systematic review and meta-analysis of longitudinal studies. *Ageing Res Rev* 2019;**52**:7-16.

9 Penninkilampi R, Casey AN, Singh MF*, et al.* The Association between Social Engagement, Loneliness, and Risk of Dementia: A Systematic Review and Meta-Analysis. *J Alzheimers Dis* 2018;**66**:1619-33.

10 Sundstrom A, Nordin Adolfsson A, Nordin M*, et al.* Loneliness increases the risk of all-cause dementia and Alzheimer's disease. *The journals of gerontology Series B, Psychological sciences and social sciences* 2019.

11 Bycroft C, Freeman C, Petkova D*, et al.* The UK Biobank resource with deep phenotyping and genomic data. *Nature* 2018;**562**:203-9.

12 Licher S, Ahmad S, Karamujic-Comic H*, et al.* Genetic predisposition, modifiable-risk-factor profile and long-term dementia risk in the general population. *Nat Med* 2019;**25**:1364-9.

13 Wu J, Hasselgren C, Zettergren A*, et al.* The impact of social networks and APOE epsilon4 on dementia among older adults: tests of possible interactions. *Aging Ment Health* 2020;**24**:395-404.

14 Torkamani A, Wineinger NE, Topol EJ. The personal and clinical utility of polygenic risk scores. *Nat Rev Genet* 2018;**19**:581-90.

15 Sugrue LP, Desikan RS. What Are Polygenic Scores and Why Are They Important? *JAMA* 2019;**321**:1820-1.

16 Palmer LJ. UK Biobank: bank on it. *Lancet* 2007;**369**:1980-2.

17 Elovainio M, Hakulinen C, Pulkki-Raback L*, et al.* Contribution of risk factors to excess mortality in isolated and lonely individuals: an analysis of data from the UK Biobank cohort study. *Lancet Public Health* 2017;**2**:E260-E6.

18 Hakulinen C, Pulkki-Raback L, Virtanen M*, et al.* Social isolation and loneliness as risk factors for myocardial infarction, stroke and mortality: UK Biobank cohort study of 479 054 men and women. *Heart* 2018.

For peer review only

19 Hughes ME, Waite LJ, Hawkley LC*, et al.* A Short Scale for Measuring Loneliness in Large Surveys: Results From Two Population-Based Studies. *Res Aging* 2004;**26**:655-72.

20 Purcell S, Neale B, Todd-Brown K*, et al.* PLINK: a tool set for whole-genome association and population-based linkage analyses. *Am J Hum Genet* 2007;**81**:559-75.

21 Choi SW, O'Reilly PF. PRSice-2: Polygenic Risk Score Software for Biobank-Scale Data. *GigaScience* 2019;**8**:July1, 2019.

22 Euesden J, Lewis CM, O'Reilly PF. PRSice: Polygenic Risk Score software. *Bioinformatics* 2015;**31**:1466-8.

23 Escott-Price V, Sims R, Bannister C*, et al.* Common polygenic variation enhances risk prediction for Alzheimer's disease. *Brain* 2015;**138**:3673-84.

24 Escott-Price V, Myers A, Huentelman M*, et al.* Polygenic Risk Score Analysis of Alzheimer's Disease in Cases without APOE4 or APOE2 Alleles. *J Prev Alzheimers Dis* 2019;**6**:16-9.

25 Spitzer RL, Kroenke K, Williams JB. Validation and utility of a self-report version of PRIME-MD: the PHQ primary care study. Primary Care Evaluation of Mental Disorders. Patient Health Questionnaire. *JAMA* 1999;**282**:1737-44.

26 Andersen PK, Geskus RB, de Witte T*, et al.* Competing risks in epidemiology: possibilities and pitfalls. *Int J Epidemiol* 2012;**41**:861-70.

27 Andersen PK, Keiding N. Interpretability and importance of functionals in competing risks and multistate models. *Stat Med* 2012;**31**:1074-88.

28 White I, Royston P. Imputing missing covariate values for the Cox model. *Statistical Medicine* 2009;**28**:1982-98.

29 Lourida I, Hannon E, Littlejohns TJ*, et al.* Association of Lifestyle and Genetic Risk With Incidence of Dementia. *JAMA* 2019.

30 Lambert JC, Ibrahim-Verbaas CA, Harold D*, et al.* Meta-analysis of 74,046 individuals identifies 11 new susceptibility loci for Alzheimer's disease. *Nature Genetics* 2013;**45**:1452-U206.

31 Sommerlad A, Sabia S, Singh-Manoux A*, et al.* Association of social contact with dementia and cognition: 28-year follow-up of the Whitehall II cohort study. *PLoS Med* 2019;**16**:e1002862.

32 Jack CR, Jr., Knopman DS, Jagust WJ*, et al.* Tracking pathophysiological processes in Alzheimer's disease: an updated hypothetical model of dynamic biomarkers. *Lancet Neurol* 2013;**12**:207-16.

33 McEwen BS. Allostasis, allostatic load, and the aging nervous system: role of excitatory amino acids and excitotoxicity. *Neurochem Res* 2000;**25**:1219-31.

34 Cacioppo S, Grippo AJ, London S*, et al.* Loneliness: clinical import and interventions. *Perspect Psychol Sci* 2015;**10**:238-49.

35 Kivimaki M, Singh-Manoux A, Pentti J*, et al.* Physical inactivity, cardiometabolic disease, and risk of dementia: an individual-participant meta-analysis. *BMJ* 2019;**365**:l1495.

36 Cacioppo JT, Hawkley LC, Thisted RA. Perceived social isolation makes me sad: 5-year cross-lagged analyses of loneliness and depressive symptomatology in the Chicago Health, Aging, and Social Relations Study. *Psychol Aging* 2010;**25**:453-63.

37 Diniz BS, Butters MA, Albert SM*, et al.* Late-life depression and risk of vascular dementia and Alzheimer's disease: systematic review and meta-analysis of community-based cohort studies. *Br J Psychiatry* 2013;**202**:329-35.

38 Scarmeas N, Stern Y. Cognitive reserve and lifestyle. *J Clin Exp Neuropsychol* 2003;**25**:625-33.

39 Berkman LF. Social support, social networks, social cohesion and health. *Soc Work Health Care* 2000;**31**:3-14.

For peer review only

40 Wilkinson T, Schnier C, Bush K*, et al.* Identifying dementia outcomes in UK Biobank: a validation study of primary care, hospital admissions and mortality data. *Eur J Epidemiol* 2019;**34**:557-65.
41 Fry A, Littlejohns TJ, Sudlow C*, et al.* Comparison of Sociodemographic and Health-Related Characteristics of UK Biobank Participants With Those of the General Population. *Am J Epidemiol* 2017;**186**:1026-34.
42 Batty GD, Gale CR, Kivimaki M*, et al.* Comparison of risk factor associations in UK Biobank against representative, general population based studies with conventional response rates: prospective cohort study and individual participant meta-analysis. *BMJ* 2020;**368**:m131.

For peer review only

Table 1. Baseline Characteristics of Participants according to diagnosed Dementia at follow-up

		Dementia		
Variables		*No*	*Yes*	*p-value*
Age at baseline	Mean (SD)	64.1 (2.8)	65.8 (2.7)	<0.001
Sex	Female	79816 (52.0)	631 (43.7)	<0.001
	Male	73803 (48.0)	813 (56.3)	
Education	Lower	40575 (26.7)	536 (38.2)	<0.001
	Intermediate	71838 (47.4)	606 (43.2)	
	Higher	39304 (25.9)	261 (18.6)	
Long-term illness	No	57734 (38.7)	319 (23.3)	<0.001
	Yes	91264 (61.3)	1053 (76.7)	
Physical activity	Low	45961 (30.7)	479 (34.9)	0.001
	High	103933 (69.3)	893 (65.1)	
Current smoker	No	140640 (92.0)	1281 (89.4)	<0.001
	Yes	12264 (8.0)	152 (10.6)	
Alcohol consumption	Lower	81237 (52.9)	866 (60.1)	<0.001
	Higher	72281 (47.1)	575 (39.9)	
Depressive symptoms	Low	121502 (82.5)	1014 (75.8)	<0.001
	Low-medium	21350 (14.5)	245 (18.3)	
	High_medium	2788 (1.9)	42 (3.1)	
	High	1639 (1.1)	37 (2.8)	
Townsend deprivation index	Mean (SD)	-1.7 (2.8)	-1.1 (3.3)	<0.001
Socially isolated	No	138407 (91.5)	1208 (87.3)	<0.001
	Yes	12922 (8.5)	175 (12.7)	
Feeling lonely	No	138250 (94.5)	1253 (92.5)	0.001
	Yes	7999 (5.5)	102 (7.5)	
Genetic dementia risk	Low	51355 (33.4)	333 (23.1)	<0.001
	Intermediate	51171 (33.3)	517 (35.8)	
	High	51093 (33.3)	594 (41.1)	

For peer review only

2

Apolipoprotein E genotype	None	113994 (74.2)	707 (49.0)	<0.001
	One e4 allele	36103 (23.5)	568 (39.3)	
	Two e4 alleles	3522 (2.3)	169 (11.7)	

For peer review only

2

Table 2. Association between genetic risk and risk of incident dementia. The values are hazard ratios (HR) and 95% confidence intervals (95% CI)

	Model 1		Model 2	
Predictor	HR (95% CI)	P-Value	HR (95% CI)	P-Value
Intermediate genetic risk vs. low	1.56 (1.36 – 1.79)	**<0.001**	1.49 (1.28 – 1.73)	**<0.001**
High genetic risk vs. low	1.79 (1.56 – 2.04)	**<0.001**	1.71 (1.47 – 1.98)	**<0.001**
Observations	155063		139345	

Model 1. Adjusted for age and sex
Model 2. Adjusted for age, sex, education, social deprivation, health behaviours, long-term illness, depressive symptoms, and 10 principal components

For peer review only

Table 3. Associations of loneliness and isolation with incident dementia. The values are Hazard ratios (HR) and 95% confidence intervals (95% CI)

	**Model 1**		**Model 2**		**Model 3**	
Separate analyses						
Predictor	*HR (95% CI)*	*P-Value*	*HR (95% CI)*	*P-Value*	*HR (95% CI)*	*P-Value*
Lonely vs not lonely	1.47 (1.20 – 1.80)	**<0.001**	1.03 (0.81 – 1.30)	0.817	1.04 (0.82 – 1.32)	0.752
Isolated vs no isolated	1.62 (1.39 – 1.90)	**<0.001**	1.34 (1.12 – 1.60)	**0.002**	1.34 (1.12 – 1.60)	**0.002**
Combined analyses						
	HR (95% CI)	*P-Value*	*HR (95% CI)*	*P-Value*	*HR (95% CI)*	*P-Value*
Lonely vs not lonely	1.28 (1.03 – 1.59)	**0.024**	0.95 (0.74 – 1.22)	0.689	0.96 (0.75 – 1.23)	0.716
Isolated vs no isolated	1.58 (1.34 – 1.86)	**<0.001**	1.33 (1.11 – 1.60)	**0.002**	1.33 (1.11 – 1.60)	**0.003**
Observations	147604 /152712		133885 /137894		133885 /137894	

Model 1. Adjusted for age and sex
Model 2. Adjusted for age, sex, education, social deprivation, health behaviours, long-term illness, depressive symptoms, genetic risk and 10 principal components
Model 3. Adjusted for age, sex, education, social deprivation, health behaviours, long-term illness, depressive symptoms, and apolipoprotein E genotype.

Figure captions

Figure 1. Associations of social isolation with incident dementia risk in low, intermediate and high genetic risk groups.

Figure 2. Associations of loneliness with incident dementia risk risk in low, intermediate and high genetic risk groups.

Figure 3. Estimated cumulative incidence of dementia in combined genetic risk and social isolation groups.

For peer review only

Isolation Study population	N(total)	N(cases)	Hazard ratio (95% confidence interval) for dementia
**Adjusted for**			
Low genetic risk	50910	320	1.47 (1.04 to 2.07)
Intermediate genetic risk	50879	493	1.73 (1.33 to 2.24)
High genetic risk	50923	570	1.62 (1.27 to 2.08)
**+ education and social deprivation**			
Low genetic risk	50352	313	1.27 (0.90 to 1.80)
Intermediate genetic risk	50330	481	1.56 (1.20 to 2.05)
High genetic risk	50342	556	1.50 (1.17 to 1.93)
**+ health behavior**			
Low genetic risk	49540	309	1.35 (0.95 to 1.91)
Intermediate genetic risk	49483	464	1.56 (1.18 to 2.06)
High genetic risk	49472	538	1.52 (1.18 to 1.96)
**+ long-term illness and depr. symp**			
Low genetic risk	47518	288	1.43 (1.00 to 2.05)
Intermediate genetic risk	47571	441	1.55 (1.17 to 2.06)
High genetic risk	47516	505	1.42 (1.09 to 1.86)
**All above**			
Low genetic risk	46008	276	1.23 (0.85 to 1.77)
Intermediate genetic risk	45993	408	1.37 (1.01 to 1.86)
High genetic risk	45893	471	1.38 (1.04 to 1.82)

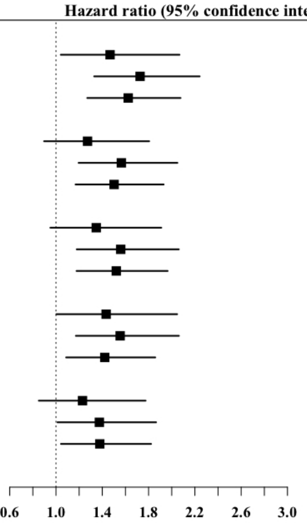

Figure 1. Associations of social isolation with incident dementia risk in low, intermediate and high genetic risk groups.

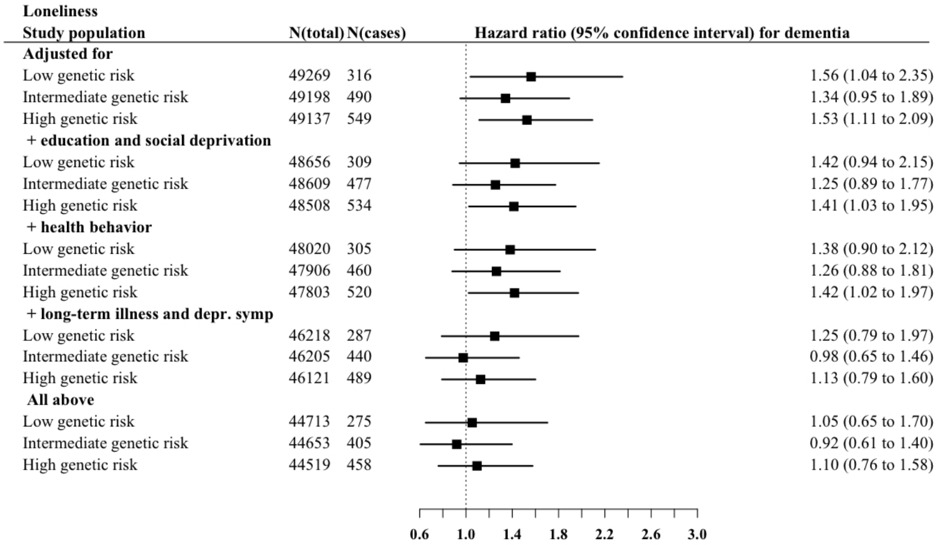

Figure 2. Associations of loneliness with incident dementia risk risk in low, intermediate and high genetic risk groups.

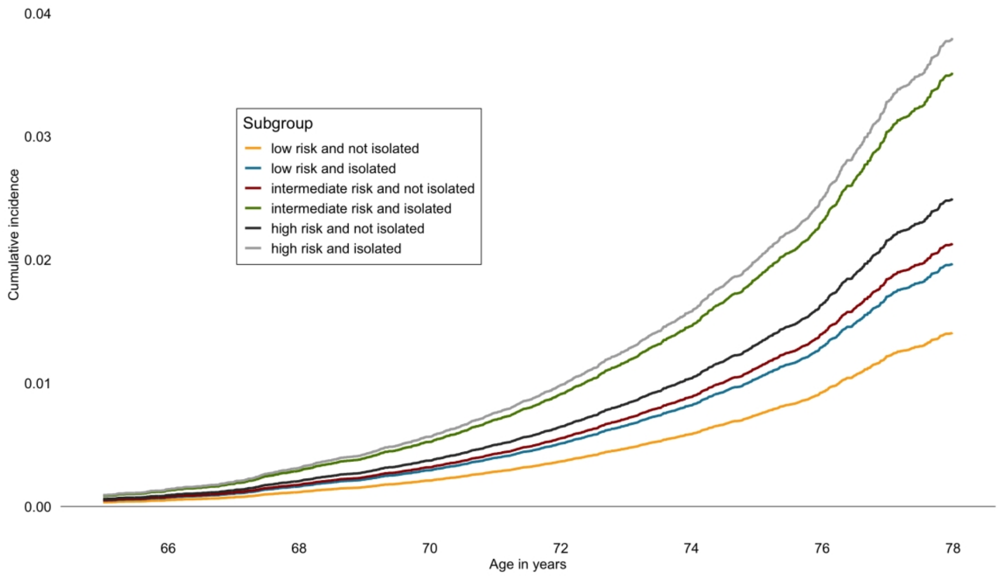

Figure 3. Estimated cumulative incidence of dementia in combined genetic risk and social isolation groups.

Supplement: Elovainio, M, Lahti, J., Pirinen, M., Pulkki-Råback, L., Lipsanen, J., Virtanen, M., Kivimäki, M., & Hakulinen, C. The association between social isolation, loneliness, and genetic risk with incidence of dementia: UK Biobank cohort study

Table of content

1) Additional information of dementia assessment
2) Additional information of genetic risk score
3) The associations between genetic risk score and incident dementia using 10 various genetic risk score cut-off points
4) Interaction effects
5) The associations of social isolation, loneliness and genetic risk score with specific Alzheimer's disease
6) The associations of social isolation, loneliness and genetic risk score with incident dementia using imputed data

For peer review only

1) Additional information of dementia assessment

Incident all-cause dementia was defined using the following ICD-9 and ICD-10 codes:

ICD-9: 290.2, 290.3, 290.4, 291.2, 294.1, 331.0, 331.1, 331.2. 331.5

ICD-10: A81.0, F00, F00.0, F00.1, F00.2, F00.9, F01, F01.0, F01.1, F01.2, F01.3, F01.8, F01.9, F02, F02.0, F02.1, F02.2, F02.3, F02.4, F02.8, F03, F05.1, F10.6, G30, G30.0, G30.1, G30.8, G30.9, G31.0, G31.1, G31.8, I67.3

Incident Alzheimer's disease was defined using the following ICD-9 and ICD-10 codes:

ICD-9: 331.0

ICD-10: F00, F00.0, F00.1, F00.2, F00.9, G30, G30.0, G30.1, G30.8, G30.9

For more information of the dementia assessment see:
http://biobank.ndph.ox.ac.uk/showcase/showcase/docs/alg_outcome_dementia.pdf

For peer review only

2) Additional information of genetic risk score

International Genomics of Alzheimer's Project (IGAP) is a large three-stage study based upon genome-wide association studies (GWAS) on individuals of European ancestry. In stage 1, IGAP used genotyped and imputed data on 11,480,632 single nucleotide polymorphisms (SNPs) to meta-analyse GWAS datasets consisting of 21,982 Alzheimer’s disease cases and 41,944 cognitively normal controls from four consortia: The Alzheimer Disease Genetics Consortium (ADGC); The European Alzheimer's disease Initiative (EADI); The Cohorts for Heart and Aging Research in Genomic Epidemiology Consortium (CHARGE); and The Genetic and Environmental Risk in AD Consortium Genetic and Environmental Risk in AD/Defining Genetic, Polygenic and Environmental Risk for Alzheimer’s Disease Consortium (GERAD/PERADES). In stage 2, 11,632 SNPs were genotyped and tested for association in an independent set of 8,362 Alzheimer's disease cases and 10,483 controls. Meta-analysis of variants selected for analysis in stage 3A (n = 11,666) or stage 3B (n = 30,511) samples brought the final sample to 35,274 clinical and autopsy-documented Alzheimer’s disease cases and 59,163 controls.

3) The associations between genetic risk score and incident dementia using 10 various geneic risk score cut-off points

The associations between continuous PRS and incident dementia with various cut-off points is reported in SFigure 1 below. The bars are negative log10 -transformed p-values of the PRS-dementia association.

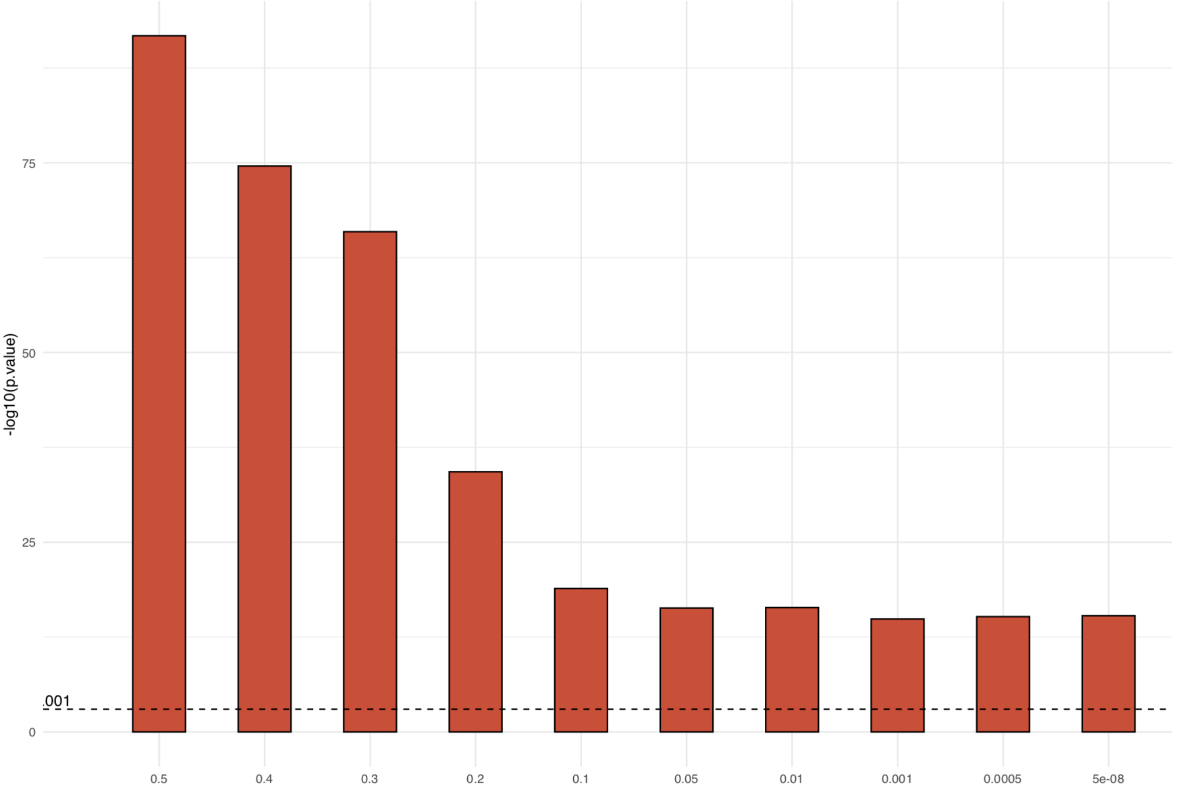

4) Interaction effects

Stable1a : Sex -genetic risk -interactions (adjusted for main effects). Figures and Hazar Ratios (HR) and 95 % confidence intervals (95% CI)

	All dementia		Alzheimer's disease	
Predictor	*HR (95% CI)*	*P-Value*	*HR (95% CI)*	*P-Value*
Sex (male) * intermediate genetic risk	0.82 (0.62 – 1.08)	0.151	0.76 (0.48 – 1.20)	0.238
Sex (male) * high genetic risk	0.84 (0.64 – 1.10)	0.197	0.74 (0.48 – 1.14)	0.168
Observations	155070		155070	

Stable 1b : Sex - loneliness and sex -isolation -interactions (adjusted for main effects). Figures and Hazar Ratios (HR) and 95 % confidence intervals (95% CI)

	All dementia		Alzheimer's disease	
Predictor	*HR (95% CI)*	*P-Value*	*HR (95% CI)*	*P-Value*
Sex (male) * lonelyd	1.36 (0.90 – 2.04)	0.143	2.63 (1.18 – 5.90)	0.019
Sex (male) * isolated	0.90 (0.66 – 1.24)	0.533	1.13 (0.68 – 1.90)	0.640
Observations	147610 /152719		147610 / 152719	

STable 1c: Genetic risk - loneliness and genetic risk -isolation -interactions (adjusted for age, sex and main effects). Figures and Hazar Ratios (HR) and 95 % confidence intervals (95% CI)

	All dementia		Alzheimer's disease	
Predictor	*HR (95%CI)*	*P-Value*	*HR (95%CI)*	*P-Value*
Intermediate genetic risk *lonelyd	0.86 (0.51 – 1.47)	0.586	1.71 (0.45 – 6.62)	0.431
High genetic risk * lonelyd	0.98 (0.58 – 1.65)	0.945	3.25 (0.94 – 11.28)	0.063
Observations	147610		147610	

	All dementia		Alzheimer's disease	
Predictor	*HR (95%CI)*	*P-Value*	*HR (95%CI)*	*P-Value*

For peer review only

Intermediate genetic risk * isolated	1.18 (0.77 – 1.81)	0.449	1.22 (0.59 – 2.52)	0.587
High genetic risk * isolated	1.11 (0.73 – 1.69)	0.624	1.15 (0.58 – 2.31)	0.682
Observations	152719		152719	

For peer review only

5) The associations of social isolation, loneliness and genetic risk score with specific Alzheimer's disease

We repeated all the analyses using specific Alzheimer's disease as the outcome instead of incident dementia and the results were materially the same, although there were, of course, much less Alzheimer's disease cases.

STable 2. Risk of Incident Alzheimers' Disease According to Genetic Risk

	**Model 1**		**Model 2**	
Genetic risk	*HR 95 % CI*	*P-Value*	*HR 95 % CI*	*P-Value*
Intermediate	1.51 (1.20 – 1.90)	**<0.001**	1.42 (1.11 – 1.82)	**<0.001**
High	1.98 (1.59 – 2.45)	**<0.001**	1.91 (1.51 – 2.41)	**<0.001**
Observations	155063		139345	

Model 1. Adjusted for age, sex, and 10 principal components
Model 2. Adjusted for age, sex, 10 principal components, education, social deprivation, depressive symptoms, health behaviors, loneliness and social isolation

For peer review only

STable 3. Associations of loneliness and isolation with Alzheimer' disease. The figures are Hazard ratios (HR) and 95% confidence intervals (95% CI)

	**Model 1**		**Model 2**		**Model 3**	
	Separate analyses					
Predictor	*HR (95% CI)*	*P-Value*	*HR (95% CI)*	*P-Value*	*HR (95% CI)*	*P-Value*
Lonely vs not lonely	1.05 (0.72 – 1.52)	0.809	0.85 (0.55 – 1.31)	0.450	0.86 (0.55 – 1.33)	0.503
Isolated vs no isolated	1.56 (1.21 – 2.02)	**<0.001**	1.40 (1.05 – 1.88)	**0.024**	1.41 (1.05 – 1.89)	**0.021**
	Combined analyses					
	HR (95% CI)	*P-Value*	*HR (95% CI)*	*P-Value*	*HR (95% CI)*	*P-Value*
Lonely vs not lonely	0.95 (0.64 – 1.40)	0.774	0.84 (0.51 – 1.26)	0.346	0.81 (0.52 – 1.27)	0.716
Isolated vs no isolated	1.54 (1.18 – 2.02)	**0.002**	1.41 (1.04 – 1.91)	**0.025**	1.42 (1.05 – 1.93)	**0.022**
Observations	147604 /152712		133885 /137894		133885 /137894	

Model 1. Adjusted for age and sex
Model 2. Adjusted for age, sex, education, social deprivation, health behaviours, long-term illness, depressive symptoms, genetic risk and 10 principal components
Model 3. Adjusted for age, sex, education, social deprivation, health behaviours, long-term illness, depressive symptoms, and apolipoprotein E genotype.

For peer review only

6) The associations of social isolation, loneliness and genetic risk score with incident dementia using imputed data

The number of missing values was relatively small (only less the 5% had missing values), but we repeated the final models using five imputed data sets and, not surprisingly, the results were materially not changed (sTable 4).

STable 4. Associations of loneliness and isolation with incident dementia with imputed data. The figures are Hazard ratios (HR) and 95% confidence intervals (95% CI)

	Model 1		Model 2		Model 3	
Separate analyses						
Predictor	*HR (95% CI)*	*P-Value*	*HR (95% CI)*	*P-Value*	*HR (95% CI)*	*P-Value*
Lonely vs not lonely	1.44 (1.17 – 1.77)	**<0.001**	1.01 (0.81 – 1.27)	0.901	1.02 (0.82 – 1.28)	0.832
Isolated vs no isolated	1.63 (1.39 – 1.92)	**<0.001**	1.34 (1.14 – 1.58)	**0.001**	1.34 (1.14 – 1.58)	**0.001**
Combined analyses						
	HR (95% CI)	*P-Value*	*HR (95% CI)*	*P-Value*	*HR (95% CI)*	*P-Value*
Lonely vs not lonely	1.32 (1.08 – 1.63)	**0.013**	0.97 (0.78 – 1.21)	0.804	0.98 (0.78 – 1.22)	0.844
Isolated vs no isolated	1.58 (1.35 – 1.86)	**<0.001**	1.35 (1.14– 1.59)	**0.002**	1.35 (1.15 – 1.60)	**0.003**
Observations	155063		155063		155063	

Model 1. Adjusted for age and sex
Model 2. Adjusted for age, sex, education, social deprivation, health behaviours, long-term illness, depressive symptoms, genetic risk and 10 principal components
Model 3. Adjusted for age, sex, education, social deprivation, health behaviours, long-term illness, depressive symptoms, and apolipoprotein E genotype.

For peer review only

STROBE (Strengthening The Reporting of OBservational Studies in Epidemiology) Checklist

A checklist of items that should be included in reports of observational studies. You must report the page number in your manuscript where you consider each of the items listed in this checklist. If you have not included this information, either revise your manuscript accordingly before submitting or note N/A.

Note: An Explanation and Elaboration article discusses each checklist item and gives methodological background and published examples of transparent reporting. The STROBE checklist is best used in conjunction with this article (freely available on the Web sites of PLoS Medicine at http://www.plosmedicine.org/, Annals of Internal Medicine at http://www.annals.org/, and Epidemiology at http://www.epidem.com/). Information on the STROBE Initiative is available at www.strobe-statement.org.

Section and Item	Item No.	Recommendation	Reported on Page No.
**Title and Abstract**	1	(*a*) Indicate the study's design with a commonly used term in the title or the abstract	2-3
		(*b*) Provide in the abstract an informative and balanced summary of what was done and what was found	2-3
**Introduction**			
Background/Rationale	2	Explain the scientific background and rationale for the investigation being reported	5
Objectives	3	State specific objectives, including any prespecified hypotheses	5
**Methods**			
Study Design	4	Present key elements of study design early in the paper	5-6
Setting	5	Describe the setting, locations, and relevant dates, including periods of recruitment, exposure, follow-up, and data collection	5-6
Participants	6	(*a*) *Cohort study*—Give the eligibility criteria, and the sources and methods of selection of participants. Describe methods of follow-up	6 -7
		Case-control study—Give the eligibility criteria, and the sources and methods of case ascertainment and control selection. Give the rationale for the choice of cases and controls	
		Cross-sectional study—Give the eligibility criteria, and the sources and methods of selection of participants	
		(*b*) *Cohort study*—For matched studies, give matching criteria and number of exposed and unexposed	
		Case-control study—For matched studies, give matching criteria and the number of controls per case	
Variables	7	Clearly define all outcomes, exposures, predictors, potential confounders, and effect modifiers. Give diagnostic criteria, if applicable	6-9

For peer review only

Section and Item	Item No.	Recommendation	Reported on Page No.
Data Sources/ Measurement	8*	For each variable of interest, give sources of data and details of methods of assessment (measurement). Describe comparability of assessment methods if there is more than one group	6-9
Bias	9	Describe any efforts to address potential sources of bias	9-10
Study Size	10	Explain how the study size was arrived at	6
Quantitative Variables	11	Explain how quantitative variables were handled in the analyses. If applicable, describe which groupings were chosen and why	6-9
Statistical Methods	12	(*a*) Describe all statistical methods, including those used to control for confounding	9
		(*b*) Describe any methods used to examine subgroups and interactions	9
		(*c*) Explain how missing data were addressed	9-10
		(*d*) *Cohort study*—If applicable, explain how loss to follow-up was addressed	9-10
		Case-control study—If applicable, explain how matching of cases and controls was addressed	
		Cross-sectional study—If applicable, describe analytical methods taking account of sampling strategy	
		(*e*) Describe any sensitivity analyses	9-10
**Results**			
Participants	13*	(a) Report numbers of individuals at each stage of study—eg numbers potentially eligible, examined for eligibility, confirmed eligible, included in the study, completing follow-up, and analysed	6
		(b) Give reasons for non-participation at each stage	6
		(c) Consider use of a flow diagram	
Descriptive Data	14*	(a) Give characteristics of study participants (eg demographic, clinical, social) and information on exposures and potential confounders	10
		(b) Indicate number of participants with missing data for each variable of interest	10
		(c) *Cohort study*—Summarise follow-up time (eg, average and total amount)	10
Outcome Data	15*	*Cohort study*—Report numbers of outcome events or summary measures over time	10 -11
		Case-control study—Report numbers in each exposure category, or summary measures of exposure	
		Cross-sectional study—Report numbers of outcome events or summary measures	

For peer review only

Section and Item	Item No.	Recommendation	Reported on Page No.
Main Results	16	(*a*) Give unadjusted estimates and, if applicable, confounder-adjusted estimates and their precision (eg, 95% confidence interval). Make clear which confounders were adjusted for and why they were included	10-11
		(*b*) Report category boundaries when continuous variables were categorized	6-9
		(*c*) If relevant, consider translating estimates of relative risk into absolute risk for a meaningful time period	11
Other Analyses	17	Report other analyses done—eg analyses of subgroups and interactions, and sensitivity analyses	12
**Discussion**			
Key Results	18	Summarise key results with reference to study objectives	12
Limitations	19	Discuss limitations of the study, taking into account sources of potential bias or imprecision. Discuss both direction and magnitude of any potential bias	14
Interpretation	20	Give a cautious overall interpretation of results considering objectives, limitations, multiplicity of analyses, results from similar studies, and other relevant evidence	12 -15
Generalisability	21	Discuss the generalisability (external validity) of the study results	14
**Other Information**			
Funding	22	Give the source of funding and the role of the funders for the present study and, if applicable, for the original study on which the present article is based	16-17

*Give information separately for cases and controls in case-control studies and, if applicable, for exposed and unexposed groups in cohort and cross-sectional studies.

Once you have completed this checklist, please save a copy and upload it as part of your submission. DO NOT include this checklist as part of the main manuscript document. It must be uploaded as a separate file.

For peer review only

BMJ Open

Association of social isolation, loneliness, and genetic risk with incidence of dementia: UK Biobank cohort study

Journal:	*BMJ Open*
Manuscript ID	bmjopen-2021-053936.R2
Article Type:	Original research
Date Submitted by the Author:	24-Jan-2022

Complete List of Authors:	Elovainio, Marko; University of Helsinki; Finnish Institute for Health and Welfare<br>Lahti, Jari; University of Helsinki<br>Pirinen, Matti; University of Helsinki; University of Helsinki, Department of Public Health<br>Pulkki-Raback, Laura; University of Helsinki, Psychology and logopedics<br>Malmberg, Anni; University of Helsinki<br>Lipsanen, Jari; University of Helsinki, Psychology and logopedics<br>Virtanen, Marianna; Itä-Suomen yliopisto, School of Educational Sciences and Psychology<br>Kivimaki, Mika; University College London, Department of Epidemiology & Public Health<br>Hakulinen, Christian; University of Helsinki, Psychology and logopedics

**Primary Subject Heading**:	Epidemiology
Secondary Subject Heading:	Neurology, Public health
Keywords:	Dementia < NEUROLOGY, Public health < INFECTIOUS DISEASES, GENETICS, GERIATRIC MEDICINE

SCHOLARONE™ Manuscripts

I, the Submitting Author has the right to grant and does grant on behalf of all authors of the Work (as defined in the below author licence), an exclusive licence and/or a non-exclusive licence for contributions from authors who are: i) UK Crown employees; ii) where BMJ has agreed a CC-BY licence shall apply, and/or iii) in accordance with the terms applicable for US Federal Government officers or employees acting as part of their official duties; on a worldwide, perpetual, irrevocable, royalty-free basis to BMJ Publishing Group Ltd ("BMJ") its licensees and where the relevant Journal is co-owned by BMJ to the co-owners of the Journal, to publish the Work in this journal and any other BMJ products and to exploit all rights, as set out in our licence.

The Submitting Author accepts and understands that any supply made under these terms is made by BMJ to the Submitting Author unless you are acting as an employee on behalf of your employer or a postgraduate student of an affiliated institution which is paying any applicable article publishing charge ("APC") for Open Access articles. Where the Submitting Author wishes to make the Work available on an Open Access basis (and intends to pay the relevant APC), the terms of reuse of such Open Access shall be governed by a Creative Commons licence – details of these licences and which Creative Commons licence will apply to this Work are set out in our licence referred to above.

Other than as permitted in any relevant BMJ Author's Self Archiving Policies, I confirm this Work has not been accepted for publication elsewhere, is not being considered for publication elsewhere and does not duplicate material already published. I confirm all authors consent to publication of this Work and authorise the granting of this licence.

For peer review only

24 January 2022

Association of social isolation, loneliness, and genetic risk with incidence of dementia: UK Biobank cohort study

Marko Elovainio, PhD[1,2]
Jari Lahti, PhD[1]
Matti Pirinen, PhD[3,4,5]
Laura Pulkki-Råback, PhD [1,6]
Anni Malmberg, BA[1]
Jari Lipsanen, MSci [1]
Marianna Virtanen, PhD [7]
Mika Kivimäki, FMedSci [4,8]
Christian Hakulinen, PhD [1]

[1]Department of Psychology and Logopedics, Research Program Unit, Faculty of Medicine, University of Helsinki, Finland;
[2]Finnish Institute for Health and Welfare, Finland;
[3]Institute for Molecular Medicine Finland, Helsinki Institute of Life Sciences, University of Helsinki, Finland;
[4]Department of Public Health, University of Helsinki, Finland;
[5]Helsinki Institute for Information Technology and Department of Mathematics and Statistics, University of Helsinki, Finland;
[6]Research Centre of Child Psychiatry, Faculty of Medicine, University of Turku, Finland;
[7]School of Educational Sciences and Psychology, University of Eastern Finland, Joensuu, Finland;
[8]Department of Epidemiology and Public Health, University College London, London, UK;

Correspondence: Marko Elovainio, University of Helsinki, P.O. Box 9, 00014, Helsinki, Finland, Phone: +358 50 3020621, email: marko.elovainio@helsinki.fi
Word count for abstract / main text: 200 / 3259; Number of tables and figures: 4 +2

Keywords: Social network, social support, dementia, cognitive decline, public health

For peer review only

Background: Social isolation and loneliness have been associated with increased risk of dementia, but it is not known whether this risk is modified or confounded by genetic risk of dementia.

Methods: We used the prospective UK Biobank study with 155 070 participants (mean age 64.1 years), including self-reported social isolation and loneliness. Genetic risk was indicated using the polygenic risk score for Alzheimer's disease and the incident dementia ascertained using electronic health records.

Results: Overall, 8.6% of participants reported that they were socially isolated and 5.5% were lonely. During a mean follow-up of 8.8 years (1.36 million person-years), 1444 (0.9% of the total sample) were diagnosed with dementia. Social isolation, but not loneliness, was associated with increased risk of dementia (hazard ratio 1.62, 95% confidence interval 1.38 to 1.90). There were no interaction effects between genetic risk and social isolation or between genetic risk and loneliness predicting incident dementia. Of the participants who were socially isolated and had high genetic risk,4.4% (3.4% to 5.5%) were estimated to developed dementia compared with 2.9% (2.6% to 3.2%) of those who were not socially isolated but had high genetic risk. Comparable differences were also in those with intermediate and low genetic risk levels.

Conclusions: Socially isolated individuals are at increased risk of dementia at all levels of genetic risk.

For peer review only

What is already known on this topic

- Social isolation and loneliness have been associated with increased risk of dementia
- It is not known whether this risk is modified or confounded by genetic risk of dementia

What this study adds

- This is the first study to show that social isolation is associated with increased risk of dementia across the spectrum of genetic risk.
- Loneliness, in contrast to social isolation, seems to be less consistently associated with dementia when combined with genetic risk.

Article summary

- We showed that socially isolated individuals have higher risk for dementia across the spectrum of genetic risk.
- This study suggests that social isolation is a risk factor of its own, over and above genetic risk.

For peer review only

Strengths and limitations of the study

- The strengths of the study were its large sample size and a genome-wide study using a well-established polygenic risk score for dementia.
- Despite the large sample size, the sample was not representative of the UK population.
- As dementia was derived from hospital records, people with non-diagnosed dementia may have been missed.
- Reverse causation may have affected the findings by making people with pre-clinical dementia more socially isolated.
- Future research should examine the mechanistic pathways whereby social isolation is associated with dementia.

For peer review only

The rapidly rising numbers of people with dementia [1] is a significant health policy and health service concern in many high-income countries. Although considerable share of the dementia risk is due to genetic factors [2, 3], major efforts have been directed towards the identification of potentially modifiable risk factors that could prevent or delay the onset of dementia [4]. Higher levels of social support have been suggested to protect from dementia [5], with both social isolation and feelings of loneliness being associated with increased risk of dementia [6-8], although mixed findings have been reported between loneliness and dementia risk [9, 10]. However, it remains unclear whether there is an interplay between genetic factors and social isolation and loneliness (*i.e.* whether the association of social isolation and loneliness with dementia is evident only at high or low levels of genetic risk) or whether the associations of genetic factors and social network characteristics with dementia are independent and additive.

The polygenic risk score (PRS) for Alzheimer's disease, describing the polygenic burden captured by the most recent genome-wide studies [11], allows to estimate the size of the genetic risk and the extent to which the associations of social isolation and loneliness with dementia are modified by genetic risk. Existing studies have included APOE genotype as the genetic risk, focused on wider psychosocial characteristics [12], relied on small samples [13], and provided limited evidence for the interplay of genetic risk and social relations predicting the increased risk of incident dementia. In the present study, we used data from UK Biobank study to examine whether genetic risk may intensify and attenuate the associations of social isolation and loneliness with the risk of dementia. In addition to estimating relative risk, we will provide estimates of absolute risk [14], as they are important information for risk communication and clinical risk prediction [15].

METHODS

Study design and participants

For peer review only

In this analysis of the UK Biobank study, we used baseline data and obtained information of incident dementia at follow-up via linked electronic health records [16]. UK National Health Service (NHS) registers maintain records of all individuals legally registered as residents in the United Kingdom. In the UK Biobank study, these records were used to invite around 9.2 million individuals aged 40–69 years living within a sensible travelling distance of the 22 assessment centres across Great Britain 2007–2010 [16]. At the study baseline, participants completed multiple touchscreen computer-based questionnaires followed by a face-to-face interview with trained research staff. Physical measures were also taken. Details of these assessments and variables are publicly available from the UK Biobank website: http://biobank.ctsu.ox.ac.uk/crystal/.

In total, 502,656 individuals were recruited (5.4% of the eligible population). Of those, individuals that were 60 year or older and had complete data on social isolation, loneliness, dementia and genetic data were included in the present analysis (N = 147 614 – 152 070). There were 7459 (4.8%) missing values in loneliness measures and 2351 (1.5%) missing values in isolation measures. We also repeated the analyses using imputed data in those with missing information on social isolation, loneliness or other explanatory variables but had information on genetic risk score (N = 155 063). This study was conducted under generic approval from the NHS National Research Ethics Service (17th June 2011, Ref 11/NW/0382). Participants provided electronic consent for the baseline assessments and register linkage.

Ascertainment of incident dementia

Dementia was ascertained using hospital inpatient records which contains data on admissions and diagnoses from the Hospital Episode Statistics for England, Scottish Morbidity Record data for Scotland, and the Patient Episode Database for Wales. Additional cases were detected through linkage to death register data provided by the National Health Service Digital for England and Wales and the Information and Statistics Division for Scotland. Diagnoses were recorded using the International Classification of Diseases (ICD) coding system. Participants with dementia were

For peer review only

identified as having a primary/secondary diagnosis (hospital records) or underlying/contributory cause of death (death register) using ICD-9 and ICD-10 codes for Alzheimer disease and other dementia classifications (see supplement for details).

Measurement of social isolation and loneliness

Social isolation and loneliness were measured using the same scale as in our two previous UK Biobank studies [17, 18]. *Social isolation* scale was defined using the following three questions: (a) “Including yourself, how many people are living together in your household? Include those who usually live in the house such as students living away from home during term, partners in the armed forces or professions such as pilots” (1 point for living alone) (b) “How often do you visit friends or family or have them visit you?” (1 point for friends and family visits less than once a month), and (c) “Which of the following [leisure/social activities] do you attend once a week or more often? You can select more than one", (1 point for no participation in social activities at least weekly). This resulted in scale with a range from 0 to 3, where an individual was defined as socially isolated if he/she had two or more of those points and those who scored 0 or 1 were classified as not isolated. Other studies in the UK have used similar measures [18].

Loneliness scale was constructed from two questions: "Do you often feel lonely? " (no = 0, yes=1) and “"How often are you able to confide in someone close to you?"(0 = almost daily-once every few months 1= once every few months to never or almost never). An individual was defined as lonely if he/she responded positively to both questions (score 2) and not lonely if he or she responded negatively to one or both of the questions (score 0 -1). Similar questions have been used in longer loneliness scales, such as the Revised UCLA Loneliness Scale [19].

Polygenic risk score of dementia

For peer review only

From the genotyped UK Biobank samples, we included 155,070 unrelated white British participants after removal of participants based on heterozygosity and missingness of outliers, sex chromosome aneuploidies and mismatches, withdrawals, and those that UK Biobank had excluded from the relatedness calculations. The genotypes were imputed against Haplotype Reference Consortium and UK10K haplotype resources containing ~96M variants [11]. We calculated polygenic risk scores (PRS) for Alzheimer's disease (AD) based on a genome-wide association study by Kunkle and others (2019) with 35,274 AD cases and 59,163 controls that do not overlap with UK Biobank samples (for details see the online supplement). We used Plink 1.9 [20] for the genotype QC and clumping. The following parameters were used for the clumping of the genotype data: p-value threshold 0.5, LD threshold (r^2) 0.5, and clumping window width of 250 kilobases. Prior to clumping we excluded all SNPs with MAF < 0.001, genotyping rate < 0.1, Hardy-Weinberg equilibrium p-value < 1e-6 and missingness per person >0.1. We used PRSice 2.2.8 [21] for calculating the PRS with the genotype QC settings that have been recommended by the software developers [22]. In the main analyses, we applied a p-value threshold of 0.5, which resulted in including 626,623 SNPs in the PRS. This threshold was chosen as previous work has reported that it provided an optimal set of variants for predicting dementia and AD [23, 24]. While this set is likely to include a number of variants which are not associated with AD, it also includes a number of variants that at present do not have sufficient statistical evidence to meet the criteria for being genome-wide significant (i.e. P-value < 5x10-8) but are expected to be associated in future larger studies. The univariate associations between genetic risks score with 10 different cut-off points and incident dementia is reported in the supplement (SFigure 1). Last, based on two single nucleotide polymorphisms (rs7412 and rs429358), we additionally genotyped APOE (none, one, or, two ε4 alleles).

The polygenic risk scores were then z-standardized to have mean 0 and variance 1, and divided into tertiles and categorized as low-, intermediate- and high-risk tertiles.

For peer review only

Assessment of potential explanatory factors

Following information was used in the current study: sex, age in years, socioeconomic factors (educational attainment and Townsend deprivation index, which is an area-level composite measure of deprivation based on unemployment, non-home ownership, non-car ownership, and household overcrowding), chronic diseases (diabetes, cardiovascular disease, cancer, and other long-standing illness, disability or infirmity), cigarette smoking (smoker [yes/no]; ex-smoker[yes/no]), physical activity (moderate and vigorous physical activity), alcohol intake frequency (Three or four times a week or more vs. once or twice a week or less), and the frequency of depressed mood in the past 2 weeks (Patient Health Questionnaire; [25]).

Statistical analyses

Study participants were followed from the study baseline (2006-2010) for incident dementia until the date of first dementia diagnosis, death, or to the end of the follow-up, whichever came first. The associations of social isolation, loneliness and polygenic risk score with incident dementia were examined using Cox proportional hazard regression models where age was used as a time scale. Results from these analyses were reported as hazard ratios (relative risk) and their 95% confidence intervals and the models were adjusted for age, sex, and 10 first principal components of genetic structure from UK Biobank to control for possible population stratification, and additionally for education, social deprivation index, having long term illness, physical activity, smoking status, alcohol consumption, and depressive symptoms. In these analyses, PRS was used both as a categorical and as a continuous variable. Additional adjustments were also made for APOE genotype. Cumulative incidence (absolute risk) of dementia associated with combined categories of

For peer review only

social isolation, loneliness and genetic risk was estimated using competing-risks regression [26, 27], with death being treated as competing event.

For the sensitivity analyses, missing data on social isolation, loneliness and all explanatory factors were imputed using multiple imputation by chained equations to generate five imputed datasets. Imputation model included age, sex, social isolation, loneliness, all covariates, the Nelson-Aalen estimate of cumulative hazard, and survival status [28]. Cox proportional hazards models were fitted within each imputed dataset and combined using Rubin's rules.

P-values were 2-sided with statistical significance set at less than .05. All analyses were performed using Stata (15.1) and R (4.2.1).

Role of the funding source

The sponsors of the study had no role in study design, data collection, data analysis, data interpretation, or writing of the report. Elovainio and Hakulinen had full access to the data. Elovainio and Hakulinen take final responsibility for the decision to submit for publication.

Patient involvement

These results are based on existing data. We were not involved in the recruitment of the participants. As far as we know, no patients were engaged in designing the present research question or the outcome measures. They were also not involved in developing plans for recruitment, design, or implementation of the study, and were not asked to advise on interpretation or writing up of results. Results from UK Biobank are disseminated to study participants via the study website and social media outlets.

RESULTS

Descriptive statistics of the study participants are shown in **Table 1**. Genetic risk score data were available for 155 070 participants (51.9% women; mean age 64.1 years). Overall, 8.6% of participants (N = 13103) were classified as socially isolated and 5.5% were lonely (N = 8102). Of those who reported themselves to be socially isolated 14.3.% were also lonely. During a total of

For peer review only

1.36 million person-years (mean follow-up time 8.8 years), 1444 participants (0.9% of the total sample) were diagnosed with all-cause dementia.

As expected, a higher PRS for AD was associated with an increased risk of dementia. Using continuous PRS, the hazard ratio per 1SD increase in the score was 1.27 (95% CI 1.21 to 1.34) in an analysis adjusted for age, sex and 10 principal components. The associations between genetic risk categories (low, intermediate, and high) with incidence of dementia shown in **Table 2**. In comparison to the participants in the low genetic risk category, the hazard ratio of incident dementia was 1.49 (95% CI 1.28 – 1.73) in participants with intermediate risk and 1.71 (95% CI 1.47 – 1.98) in those with high genetic risk in the fully adjusted model. There were no interaction effects between sex and intermediate genetic risk (p = 0.15) or between sex and high genetic risk (p = 0.20) predicting incident dementia (Stable 1a).

Social isolation was associated with increased risk of dementia (HR adjusted for age and sex = 1.62, 95% CI 1.39 – 1.90). The associations attenuated but remained statistically significant after adjusting for additional covariates including socio-demographics, health-related factors and genetic risk score and principal components (HR = 1.34, 95% CI 1.12 – 1.60). Loneliness was also associated with higher risk of dementia (HR = 1.47, 95% CI 1.20 – 1.80), but this association was lost when adjusted for socio-demographics, health-related factors, PRS and principal components (HR = 1.03, 95% CI 0.81 – 1.30). Both social isolation (HR = 1.58, 95% CI 1.34 – 1.86) and loneliness (HR = 1.28, 95% CI 1.03 – 1.59) were associated with incident dementia when added simultaneously into the model but only the association between social isolation and dementia was robust to adjusting for additional covariates (HR = 1.33, 95 % CI 1.11 – 1.60). Adjusting the models for APOE produced similar associations (**Table 3**). No interaction effects between sex and isolation (p = 0.53) or between sex and loneliness (p = 0.14) predicting incident dementia were found (Stable 1b).

For peer review only

Although no significant interaction effects in the associations between social isolation and genetic risk categories (p-values range 0.45-0.62) or loneliness and genetic risk categories (p-values range 0.59-0.95) with incident dementia were found (Stable 1c), we illustrated the interplay between genetic risk with social isolation and loneliness by presenting associations at all genetic risk levels adjusting for potential confounders (Figures 1 and 2). Social isolation was associated with increasing dementia risk in all genetic risk levels. At intermediate and high genetic risk levels, these associations were robust to adjusting for all potential confounders or mediators (hazard ratio = 1.37, 95% CI, 1.01-1.86; hazard ratio= 1.38, 95% CI, 1.04-1.82). The results for loneliness were less consistent, and the risk of dementia was similar in lonely participants at low and at high levels of genetic risk, when compared with those who reported no loneliness. In the high genetic risk group, for example, the hazard ratios were 1.53 (95% CI 1.11 – 2.09) in low and 1.56 (95% CI 1.04 – 2.35) in high loneliness group (**Figure 2**). All these association were attenuated when adjusted for long-term illness and depressive symptoms and in the fully adjusted model.

In terms of absolute risk (cumulative incidence), of those who were socially isolated and had high genetic risk, 4.4% (3.4% to 5.5%) were estimated to developed dementia compared with 2.9% (2.6% to 3.2%) of those who were not socially isolated but had high genetic risk (**Figure 3**). The corresponding absolute risk estimates in the socially isolated and not isolated were 4.1 (3.1% to 5.1%) and 2.5% (2.2% to 2.8%) in participants with intermediate genetic risk and 2.3% (1.5% to 3.0%) and 1.6% (1.4% to 1.9%) in those with low genetic risk.

As sensitivity analyses, we repeated all the main analyses with Alzheimer's disease as the outcome (STables 2-3), and with missing explanatory variables imputed (STable 4). The results did not materially change. To detect whether the associations with incident dementia were due to reverse causation, we additionally repeated the fully adjusted models using data where those

For peer review only

dementia cases occurring in the first three years of the follow-up were excluded. The association between isolation and incident dementia (hazard ratio= 1.30, 95% CI, 1.08-1.58) and between loneliness and incident dementia (hazard ratio= 1.06, 95% CI, 0.82-1.36) were similar.

DISCUSSION

In this UK Biobank study of 155 063men and women, social isolation was associated with increased risk of all-cause dementia and Alzheimer's disease at intermediate and high levels of genetic risk of Alzheimer's disease. No interaction effects were found between genetic risk levels and isolation predicting incident dementia. The incidence of dementia was estimated to reach over 4% in isolated high-genetic risk individuals compared to approximately 3% in non-isolated individuals with similar genetic risk. The difference between these groups was comparable also among those with intermediate and low genetic risk. This means that among individuals with similar genetic risk for dementia, those who are socially isolated are more likely to have incidence of the disease, suggesting an effect by social isolation over and above that of genetic risk. The association between loneliness and dementia was attributable to other dementia risk factors, such as health behaviours and depressive symptoms.

The relative risk of dementia across the genetic risk categories was at the same magnitude as in a previous UK Biobank study [29] that used data from an older GWAS [30]. Our findings also support other studies - most of which with follow-ups from 5 to 11 years – showing an association of social isolation with increased risk of dementia [6, 8, 10]. A 28-year follow-up of 10,000 Whitehall II study participants found that less frequent social contacts at ages 50, 60 and 70 were associated with approximately 10% higher dementia risk, independent of socio-economic and other lifestyle factors [31]. While previous studies have produced mixed findings on whether loneliness is associated with increased risk of dementia or not [9, 10], our findings show that the association between loneliness and dementia is mostly likely explained by other factors and present

For peer review only

only at high levels of genetic risk.

Our results should be interpreted in a context of disease aetiology. Dementia is characterised by a 10-20-year preclinical or prodromal stage during which changes in biomarkers and cognitive abilities increasingly occur [32]. With a follow-up less than 10 years, it is likely that we assessed social isolation for dementia cases during this preclinical period. This could result to reverse causality, i.e., increased prevalence of social isolation during the 8-year period could have resulted from preclinical changes in social activity leading to a spurious association between social isolation and dementia.

Several mechanisms through which social isolation may causally affect dementia risk have been proposed. Social isolation and loneliness have been suggested to increase stress reactivity which is associated with prolonged activation of the hypothalamic-pituitary-adrenal axis (HPA) and the sympatho-adrenal system [33]. This process may further lead to sleep deprivation, dysregulation of the immune system, and even increased levels of oxidative stress [34], all potentially harmful for cognitive health. It has also been shown that socially isolated and lonely individuals more often engage in health-damaging behaviors [18], which may affect cognition either directly via biophysiological mechanisms or increased incidence of cardiometabolic diseases which accelerate neurodegeneration [35]. Socially isolated or lonely individuals are also at an increased risk of depression [36], a potential risk factor for cognitive decline and dementia [37]. Participation in social activities and social interaction stimulates neural plasticity by building and maintaining cognitive reserve [38]. Poor cognitive reserve is a further pathway through which social isolation and loneliness could increase dementia risk [39]. Fewer social contacts with reduced exercising of memory and language adversely affect cognitive reserve, thereby accelerating dementia onset [39]. Cognitive ability was not assessed in the present study and a small share of the found association between social isolation and subsequent dementia risk may be attributable to lower initial cognitive reserve.

For peer review only

Strengths and limitations

The major strengths of the current study include the large sample size of UK Biobank participants, which enabled us to study the combination of genetic risk, social isolation, and loneliness in detail. In addition, we used the largest genome-wide association study of dementia to date to derive the genetic risk for AD [2].

There are also some important limitations. Although our analyses were adjusted for multiple potential sources of bias, the possibility of unmeasured confounding and reverse causation cannot be ruled out. However, the results were basically unchanged when excluding those with incident dementia during the first three -year follow-up time. Both frequency of social contacts and loneliness were self-reported and measured by relatively short and crude measures. As we were able to cover the genetic risk for AD – not all-cause dementias – based on the Kunkle et al [2], we may have missed some of the genetic variance related to non-AD dementias. Dementia cases were derived from medical records or death registers, and thus some cases might have been missed. However, good agreement of dementia case determination with primary care record data has been shown [40]. This sample was restricted to volunteers of European ancestry aged 60 to 73 years at baseline and, therefore, further research is needed to ensure generalizability of our findings. As the mean age of participants was only 72 years at the end of the follow-up period, the incidence of dementia remained low. As noted previously the response rate of the UK Biobank study survey was very low, 5.5%, and UK Biobank is not representative of the sampling population [41]. However,

For peer review only

many etiological findings from UK Biobank appear to be generalisable to England and Scotland [42].

Conclusions

The present findings suggest an association between social isolation and increased risk of dementia across the spectrum of genetic risk. Further research is needed to determine the extent social isolation is a modifiable risk factor rather than a part of the dementia prodrome

For peer review only

Acknowledgments: We thank the International Genomics of Alzheimer's Project (IGAP) for providing summary results data for these analyses. The investigators within IGAP contributed to the design and implementation of IGAP and/or provided data but did not participate in analysis or writing of this report. IGAP was made possible by the generous participation of the control subjects, the patients, and their families. The i–Select chips was funded by the French National Foundation on Alzheimer's disease and related disorders. EADI was supported by the LABEX (laboratory of excellence program investment for the future) DISTALZ grant, Inserm, Institut Pasteur de Lille, Université de Lille 2 and the Lille University Hospital. GERAD/PERADES was supported by the Medical Research Council (Grant n° 503480), Alzheimer's Research UK (Grant n° 503176), the Wellcome Trust (Grant n° 082604/2/07/Z) and German Federal Ministry of Education and Research (BMBF): Competence Network Dementia (CND) grant n° 01GI0102, 01GI0711, 01GI0420. CHARGE was partly supported by the NIH/NIA grant R01 AG033193 and the NIA AG081220 and AGES contract N01–AG–12100, the NHLBI grant R01 HL105756, the Icelandic Heart Association, and the Erasmus Medical Center and Erasmus University. ADGC was supported by the NIH/NIA grants: U01 AG032984, U24 AG021886, U01 AG016976, and the Alzheimer's Association grant ADGC–10–196728.

Contributors: ME and CH designed the study and conducted the statistical analyses. ME wrote the first draft of the manuscript. JL and AM calculated the polygenetic risk score with the help of MP. All authors (ME, JL, MP, LPR, AM, JL, MV, MK, CH) contributed to the interpretation of the results and critical revision of the manuscript for important intellectual content and approved the final version of the manuscript. The corresponding author attests that all listed authors meet authorship criteria and that no others meeting the criteria have been omitted.

For peer review only

Funding: ME and CH were supported by the Academy of Finland (339390 (ME) / 310591(CH)).

MK was supported by NordForsk (70521), the UK Medical Research Council (MRC S011676), the Academy of Finland (311492), and the US National Institutes on Ageing (NIA R01AG056477). . Laura Pulkki-Råback was supported by the Jenny and Antti Wihuri Foundation. GERAD/PERADES was supported by the Medical Research Council (Grant n° 503480), Alzheimer's Research UK (Grant n° 503176), the Wellcome Trust (Grant n° 082604/2/07/Z) and German Federal Ministry of Education and Research (BMBF): Competence Network Dementia (CND) grant n° 01GI0102, 01GI0711, 01GI0420. CHARGE was partly supported by the NIH/NIA grant R01 AG033193 and the NIA AG081220 and AGES contract N01–AG–12100, the NHLBI grant R01 HL105756, the Icelandic Heart Association, and the Erasmus Medical Center and Erasmus University. ADGC was supported by the NIH/NIA grants: U01 AG032984, U24 AG021886, U01 AG016976, and the Alzheimer's Association grant ADGC–10–196728. The funding sources did not participate in the design or conduct of the study; collection, management, analysis or interpretation of the data; or preparation, review, or approval of the manuscript.

Competing interests: All authors have completed the ICMJE uniform disclosure form at www.icmje.org/coi_disclosure.pdf (available on request from the corresponding author) and declare: no support from any organisation for the submitted work, no other relationships or activities that could appear to have influenced the submitted work.

Ethical approval: Ethical approval for data collection was given by the North-West Multi-centre Research Ethics Committee. Study was carried out in accordance with the Declaration of Helsinki

For peer review only

of the World Medical Association. The ethical board of the Finnish Institute for Health and Welfare gave ethical permission to use the genetic data.

Data sharing: The genetic and phenotypic UK Biobank data are available on application to the UK Biobank (www.ukbiobank.ac.uk/). Present study was conducted using the UK Biobank Resource under Application 14801. Summary statistics from the meta-analysis of genome wide association studies in dementia are available from https://www.niagads.org/datasets/ng00075

Transparency statement: The lead authors (ME and CH) affirms that the manuscript is an honest, accurate, and transparent account of the study being reported; that no important aspects of the study have been omitted; and that any discrepancies from the study as planned (and, if relevant, registered) have been explained. Preprint: https://www.medrxiv.org/content/10.1101/2020.02.25.20027177v1

For peer review only

References

1 Ahmadi-Abhari S, Guzman-Castillo M, Bandosz P, *et al.* Temporal trend in dementia incidence since 2002 and projections for prevalence in England and Wales to 2040: modelling study. *BMJ* 2017;**358**:j2856.

2 Kunkle BW, Grenier-Boley B, Sims R, *et al.* Genetic meta-analysis of diagnosed Alzheimer's disease identifies new risk loci and implicates Abeta, tau, immunity and lipid processing. *Nat Genet* 2019;**51**:414-30.

3 Andrews SJ, Fulton-Howard B, Goate A. Interpretation of risk loci from genome-wide association studies of Alzheimer's disease. *Lancet Neurol* 2020.

4 Soto-Gordoa M, Arrospide A, Moreno-Izco F, *et al.* Projecting Burden of Dementia in Spain, 2010-2050: Impact of Modifying Risk Factors. *J Alzheimers Dis* 2015;**48**:721-30.

5 Livingston G, Sommerlad A, Orgeta V, *et al.* Dementia prevention, intervention, and care. *Lancet* 2017;**390**:2673-734.

6 Kuiper JS, Zuidersma M, Oude Voshaar RC, *et al.* Social relationships and risk of dementia: A systematic review and meta-analysis of longitudinal cohort studies. *Ageing Res Rev* 2015;**22**:39-57.

7 Lara E, Caballero FF, Rico-Uribe LA, *et al.* Are loneliness and social isolation associated with cognitive decline? *Int J Geriatr Psychiatry* 2019.

8 Lara E, Martin-Maria N, De la Torre-Luque A, *et al.* Does loneliness contribute to mild cognitive impairment and dementia? A systematic review and meta-analysis of longitudinal studies. *Ageing Res Rev* 2019;**52**:7-16.

9 Penninkilampi R, Casey AN, Singh MF, *et al.* The Association between Social Engagement, Loneliness, and Risk of Dementia: A Systematic Review and Meta-Analysis. *J Alzheimers Dis* 2018;**66**:1619-33.

10 Sundstrom A, Nordin Adolfsson A, Nordin M, *et al.* Loneliness increases the risk of all-cause dementia and Alzheimer's disease. *The journals of gerontology Series B, Psychological sciences and social sciences* 2019.

11 Bycroft C, Freeman C, Petkova D, *et al.* The UK Biobank resource with deep phenotyping and genomic data. *Nature* 2018;**562**:203-9.

12 Licher S, Ahmad S, Karamujic-Comic H, *et al.* Genetic predisposition, modifiable-risk-factor profile and long-term dementia risk in the general population. *Nat Med* 2019;**25**:1364-9.

13 Wu J, Hasselgren C, Zettergren A, *et al.* The impact of social networks and APOE epsilon4 on dementia among older adults: tests of possible interactions. *Aging Ment Health* 2020;**24**:395-404.

14 Torkamani A, Wineinger NE, Topol EJ. The personal and clinical utility of polygenic risk scores. *Nat Rev Genet* 2018;**19**:581-90.

15 Sugrue LP, Desikan RS. What Are Polygenic Scores and Why Are They Important? *JAMA* 2019;**321**:1820-1.

16 Palmer LJ. UK Biobank: bank on it. *Lancet* 2007;**369**:1980-2.

17 Elovainio M, Hakulinen C, Pulkki-Raback L, *et al.* Contribution of risk factors to excess mortality in isolated and lonely individuals: an analysis of data from the UK Biobank cohort study. *Lancet Public Health* 2017;**2**:E260-E6.

18 Hakulinen C, Pulkki-Raback L, Virtanen M, *et al.* Social isolation and loneliness as risk factors for myocardial infarction, stroke and mortality: UK Biobank cohort study of 479 054 men and women. *Heart* 2018.

For peer review only

19 Hughes ME, Waite LJ, Hawkley LC*, et al.* A Short Scale for Measuring Loneliness in Large Surveys: Results From Two Population-Based Studies. *Res Aging* 2004;**26**:655-72.
20 Purcell S, Neale B, Todd-Brown K*, et al.* PLINK: a tool set for whole-genome association and population-based linkage analyses. *Am J Hum Genet* 2007;**81**:559-75.
21 Choi SW, O'Reilly PF. PRSice-2: Polygenic Risk Score Software for Biobank-Scale Data. *GigaScience* 2019;**8**:July1, 2019.
22 Euesden J, Lewis CM, O'Reilly PF. PRSice: Polygenic Risk Score software. *Bioinformatics* 2015;**31**:1466-8.
23 Escott-Price V, Sims R, Bannister C*, et al.* Common polygenic variation enhances risk prediction for Alzheimer's disease. *Brain* 2015;**138**:3673-84.
24 Escott-Price V, Myers A, Huentelman M*, et al.* Polygenic Risk Score Analysis of Alzheimer's Disease in Cases without APOE4 or APOE2 Alleles. *J Prev Alzheimers Dis* 2019;**6**:16-9.
25 Spitzer RL, Kroenke K, Williams JB. Validation and utility of a self-report version of PRIME-MD: the PHQ primary care study. Primary Care Evaluation of Mental Disorders. Patient Health Questionnaire. *JAMA* 1999;**282**:1737-44.
26 Andersen PK, Geskus RB, de Witte T*, et al.* Competing risks in epidemiology: possibilities and pitfalls. *Int J Epidemiol* 2012;**41**:861-70.
27 Andersen PK, Keiding N. Interpretability and importance of functionals in competing risks and multistate models. *Stat Med* 2012;**31**:1074-88.
28 White I, Royston P. Imputing missing covariate values for the Cox model. *Statistical Medicine* 2009;**28**:1982-98.
29 Lourida I, Hannon E, Littlejohns TJ*, et al.* Association of Lifestyle and Genetic Risk With Incidence of Dementia. *JAMA* 2019.
30 Lambert JC, Ibrahim-Verbaas CA, Harold D*, et al.* Meta-analysis of 74,046 individuals identifies 11 new susceptibility loci for Alzheimer's disease. *Nature Genetics* 2013;**45**:1452-U206.
31 Sommerlad A, Sabia S, Singh-Manoux A*, et al.* Association of social contact with dementia and cognition: 28-year follow-up of the Whitehall II cohort study. *PLoS Med* 2019;**16**:e1002862.
32 Jack CR, Jr., Knopman DS, Jagust WJ*, et al.* Tracking pathophysiological processes in Alzheimer's disease: an updated hypothetical model of dynamic biomarkers. *Lancet Neurol* 2013;**12**:207-16.
33 McEwen BS. Allostasis, allostatic load, and the aging nervous system: role of excitatory amino acids and excitotoxicity. *Neurochem Res* 2000;**25**:1219-31.
34 Cacioppo S, Grippo AJ, London S*, et al.* Loneliness: clinical import and interventions. *Perspect Psychol Sci* 2015;**10**:238-49.
35 Kivimaki M, Singh-Manoux A, Pentti J*, et al.* Physical inactivity, cardiometabolic disease, and risk of dementia: an individual-participant meta-analysis. *BMJ* 2019;**365**:l1495.
36 Cacioppo JT, Hawkley LC, Thisted RA. Perceived social isolation makes me sad: 5-year cross-lagged analyses of loneliness and depressive symptomatology in the Chicago Health, Aging, and Social Relations Study. *Psychol Aging* 2010;**25**:453-63.
37 Diniz BS, Butters MA, Albert SM*, et al.* Late-life depression and risk of vascular dementia and Alzheimer's disease: systematic review and meta-analysis of community-based cohort studies. *Br J Psychiatry* 2013;**202**:329-35.
38 Scarmeas N, Stern Y. Cognitive reserve and lifestyle. *J Clin Exp Neuropsychol* 2003;**25**:625-33.
39 Berkman LF. Social support, social networks, social cohesion and health. *Soc Work Health Care* 2000;**31**:3-14.

For peer review only

40 Wilkinson T, Schnier C, Bush K*, et al.* Identifying dementia outcomes in UK Biobank: a validation study of primary care, hospital admissions and mortality data. *Eur J Epidemiol* 2019;**34**:557-65.
41 Fry A, Littlejohns TJ, Sudlow C*, et al.* Comparison of Sociodemographic and Health-Related Characteristics of UK Biobank Participants With Those of the General Population. *Am J Epidemiol* 2017;**186**:1026-34.
42 Batty GD, Gale CR, Kivimaki M*, et al.* Comparison of risk factor associations in UK Biobank against representative, general population based studies with conventional response rates: prospective cohort study and individual participant meta-analysis. *BMJ* 2020;**368**:m131.

For peer review only

Table 1. Baseline Characteristics of Participants according to diagnosed Dementia at follow-up

		Dementia		
Variables		*No*	*Yes*	*p-value*
Age at baseline	Mean (SD)	64.1 (2.8)	65.8 (2.7)	<0.001
Sex	Female	79816 (52.0)	631 (43.7)	<0.001
	Male	73803 (48.0)	813 (56.3)	
Education	Lower	40575 (26.7)	536 (38.2)	<0.001
	Intermediate	71838 (47.4)	606 (43.2)	
	Higher	39304 (25.9)	261 (18.6)	
Long-term illness	No	57734 (38.7)	319 (23.3)	<0.001
	Yes	91264 (61.3)	1053 (76.7)	
Physical activity	Low	45961 (30.7)	479 (34.9)	0.001
	High	103933 (69.3)	893 (65.1)	
Current smoker	No	140640 (92.0)	1281 (89.4)	<0.001
	Yes	12264 (8.0)	152 (10.6)	
Alcohol consumption	Lower	81237 (52.9)	866 (60.1)	<0.001
	Higher	72281 (47.1)	575 (39.9)	
Depressive symptoms	Low	121502 (82.5)	1014 (75.8)	<0.001
	Low-medium	21350 (14.5)	245 (18.3)	
	High_medium	2788 (1.9)	42 (3.1)	
	High	1639 (1.1)	37 (2.8)	
Townsend deprivation index	Mean (SD)	-1.7 (2.8)	-1.1 (3.3)	<0.001
Socially isolated	No	138407 (91.5)	1208 (87.3)	<0.001
	Yes	12922 (8.5)	175 (12.7)	
Feeling lonely	No	138250 (94.5)	1253 (92.5)	0.001
	Yes	7999 (5.5)	102 (7.5)	
Genetic dementia risk	Low	51355 (33.4)	333 (23.1)	<0.001
	Intermediate	51171 (33.3)	517 (35.8)	
	High	51093 (33.3)	594 (41.1)	

For peer review only

2

Apolipoprotein E genotype	None	113994 (74.2)	707 (49.0)	<0.001
	One e4 allele	36103 (23.5)	568 (39.3)	
	Two e4 alleles	3522 (2.3)	169 (11.7)	

For peer review only

2

Table 2. Association between genetic risk and risk of incident dementia. The values are hazard ratios (HR) and 95% confidence intervals (95% CI)

	Model 1		Model 2	
Predictor	HR (95% CI)	P-Value	HR (95% CI)	P-Value
Intermediate genetic risk vs. low	1.56 (1.36 – 1.79)	**<0.001**	1.49 (1.28 – 1.73)	**<0.001**
High genetic risk vs. low	1.79 (1.56 – 2.04)	**<0.001**	1.71 (1.47 – 1.98)	**<0.001**
Observations	155063		139345	

Model 1. Adjusted for age and sex
Model 2. Adjusted for age, sex, education, social deprivation, health behaviours, long-term illness, depressive symptoms, and 10 principal components

For peer review only

Table 3. Associations of loneliness and isolation with incident dementia. The values are Hazard ratios (HR) and 95% confidence intervals (95% CI)

	**Model 1**		**Model 2**		**Model 3**	
	Separate analyses					
Predictor	*HR (95% CI)*	*P-Value*	*HR (95% CI)*	*P-Value*	*HR (95% CI)*	*P-Value*
Lonely vs not lonely	1.47 (1.20 – 1.80)	**<0.001**	1.03 (0.81 – 1.30)	0.817	1.04 (0.82 – 1.32)	0.752
Isolated vs no isolated	1.62 (1.39 – 1.90)	**<0.001**	1.34 (1.12 – 1.60)	**0.002**	1.34 (1.12 – 1.60)	**0.002**
	Combined analyses					
	HR (95% CI)	*P-Value*	*HR (95% CI)*	*P-Value*	*HR (95% CI)*	*P-Value*
Lonely vs not lonely	1.28 (1.03 – 1.59)	**0.024**	0.95 (0.74 – 1.22)	0.689	0.96 (0.75 – 1.23)	0.716
Isolated vs no isolated	1.58 (1.34 – 1.86)	**<0.001**	1.33 (1.11 – 1.60)	**0.002**	1.33 (1.11 – 1.60)	**0.003**
Observations	147604 /152712		133885 /137894		133885 /137894	

Model 1. Adjusted for age and sex
Model 2. Adjusted for age, sex, education, social deprivation, health behaviours, long-term illness, depressive symptoms, genetic risk and 10 principal components
Model 3. Adjusted for age, sex, education, social deprivation, health behaviours, long-term illness, depressive symptoms, and apolipoprotein E genotype.

For peer review only

Figure captions

Figure 1. Associations of social isolation with incident dementia risk in low, intermediate and high genetic risk groups.

Figure 2. Associations of loneliness with incident dementia risk risk in low, intermediate and high genetic risk groups.

Figure 3. Estimated cumulative incidence of dementia in combined genetic risk and social isolation groups.

For peer review only

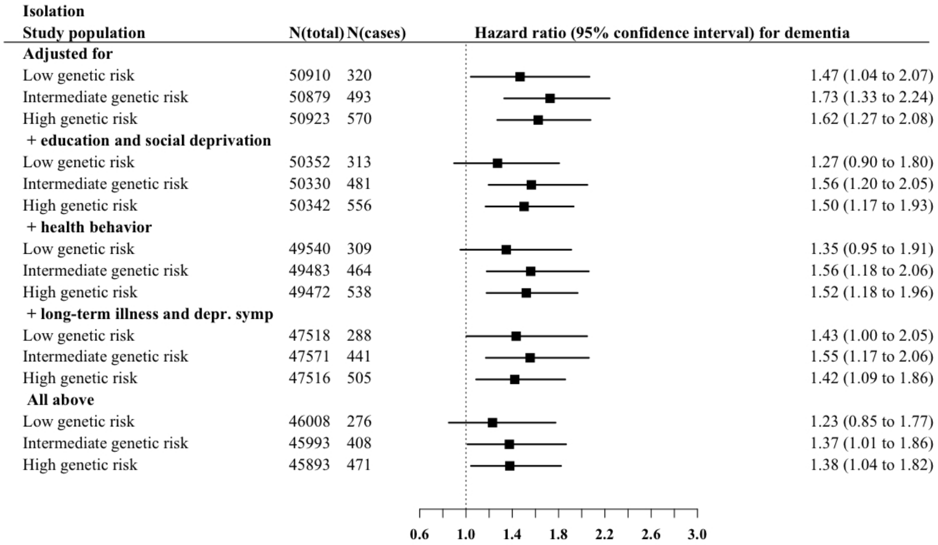

Figure 1. Associations of social isolation with incident dementia risk in low, intermediate and high genetic risk groups.

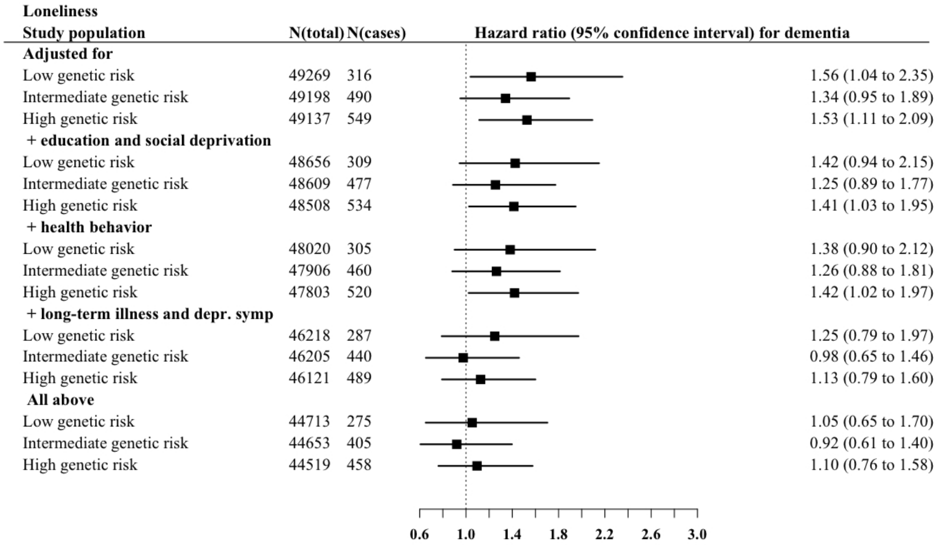

Figure 2. Associations of loneliness with incident dementia risk risk in low, intermediate and high genetic risk groups.

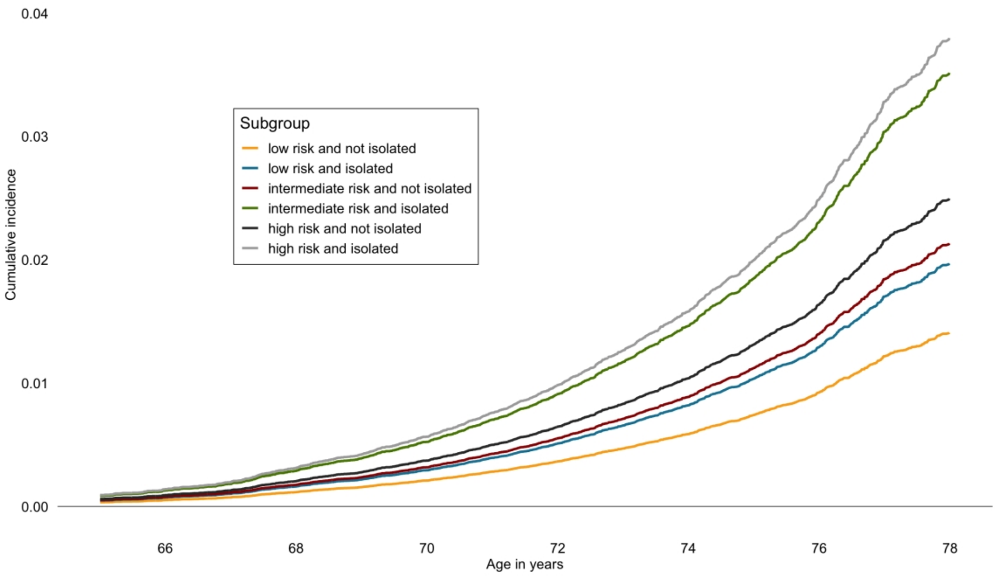

Figure 3. Estimated cumulative incidence of dementia in combined genetic risk and social isolation groups.

Supplement: Elovainio, M, Lahti, J., Pirinen, M., Pulkki-Råback, L., Lipsanen, J., Virtanen, M., Kivimäki, M., & Hakulinen, C. The association between social isolation, loneliness, and genetic risk with incidence of dementia: UK Biobank cohort study

Table of content

1) Additional information of dementia assessment
2) Additional information of genetic risk score
3) The associations between genetic risk score and incident dementia using 10 various genetic risk score cut-off points
4) Interaction effects
5) The associations of social isolation, loneliness and genetic risk score with specific Alzheimer's disease
6) The associations of social isolation, loneliness and genetic risk score with incident dementia using imputed data

For peer review only

1) Additional information of dementia assessment

Incident all-cause dementia was defined using the following ICD-9 and ICD-10 codes:

ICD-9: 290.2, 290.3, 290.4, 291.2, 294.1, 331.0, 331.1, 331.2. 331.5

ICD-10: A81.0, F00, F00.0, F00.1, F00.2, F00.9, F01, F01.0, F01.1, F01.2, F01.3, F01.8, F01.9, F02, F02.0, F02.1, F02.2, F02.3, F02.4, F02.8, F03, F05.1, F10.6, G30, G30.0, G30.1, G30.8, G30.9, G31.0, G31.1, G31.8, I67.3

Incident Alzheimer's disease was defined using the following ICD-9 and ICD-10 codes:

ICD-9: 331.0

ICD-10: F00, F00.0, F00.1, F00.2, F00.9, G30, G30.0, G30.1, G30.8, G30.9

For more information of the dementia assessment see:
http://biobank.ndph.ox.ac.uk/showcase/showcase/docs/alg_outcome_dementia.pdf

For peer review only

2) Additional information of genetic risk score

International Genomics of Alzheimer's Project (IGAP) is a large three-stage study based upon genome-wide association studies (GWAS) on individuals of European ancestry. In stage 1, IGAP used genotyped and imputed data on 11,480,632 single nucleotide polymorphisms (SNPs) to meta-analyse GWAS datasets consisting of 21,982 Alzheimer's disease cases and 41,944 cognitively normal controls from four consortia: The Alzheimer Disease Genetics Consortium (ADGC); The European Alzheimer's disease Initiative (EADI); The Cohorts for Heart and Aging Research in Genomic Epidemiology Consortium (CHARGE); and The Genetic and Environmental Risk in AD Consortium Genetic and Environmental Risk in AD/Defining Genetic, Polygenic and Environmental Risk for Alzheimer's Disease Consortium (GERAD/PERADES). In stage 2, 11,632 SNPs were genotyped and tested for association in an independent set of 8,362 Alzheimer's disease cases and 10,483 controls. Meta-analysis of variants selected for analysis in stage 3A (n = 11,666) or stage 3B (n = 30,511) samples brought the final sample to 35,274 clinical and autopsy-documented Alzheimer's disease cases and 59,163 controls.

3) The associations between genetic risk score and incident dementia using 10 various geneic risk score cut-off points

The associations between continuous PRS and incident dementia with various cut-off points is reported in SFigure 1 below. The bars are negative log10 -transformed p-values of the PRS-dementia association.

For peer review

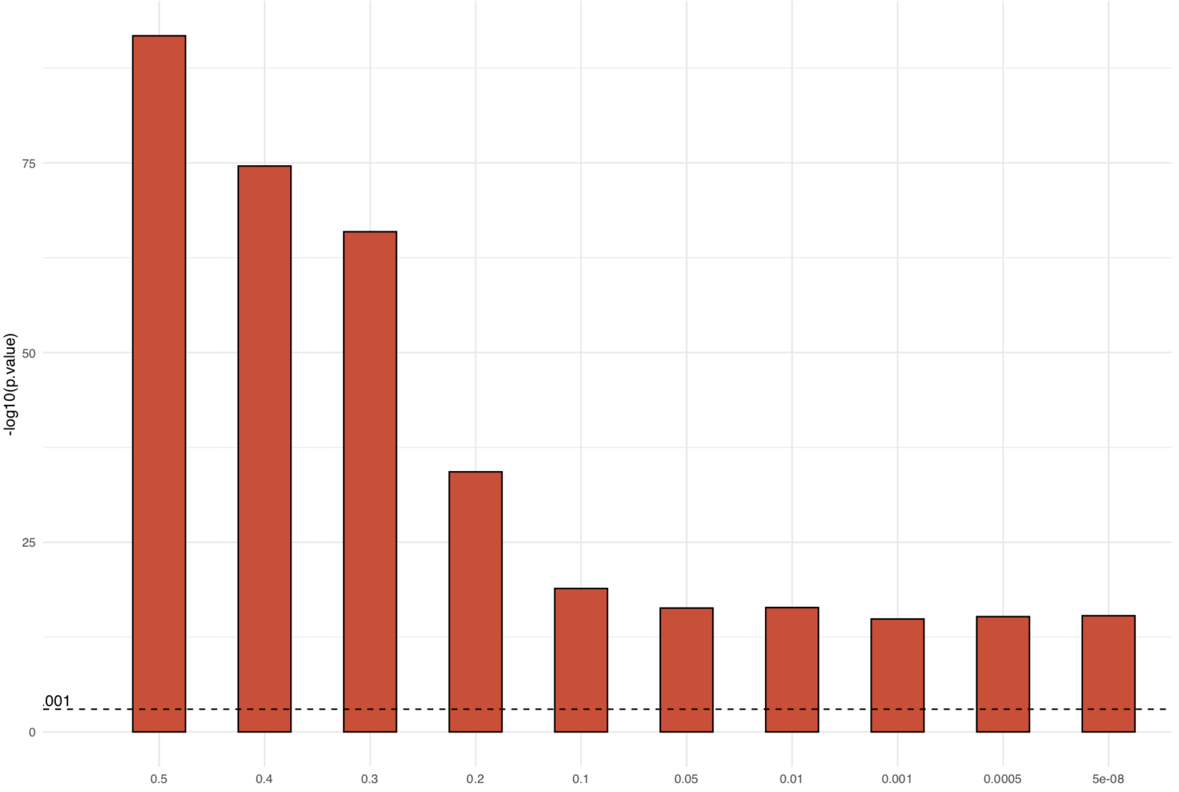

4) Interaction effects

Stable1a : Sex -genetic risk -interactions (adjusted for main effects). Figures and Hazar Ratios (HR) and 95 % confidence intervals (95% CI)

	**All dementia**		**Alzheimer's disease**	
Predictor	*HR (95% CI)*	*P-Value*	*HR (95% CI)*	*P-Value*
Sex (male) * intermediate genetic risk	0.82 (0.62 – 1.08)	0.151	0.76 (0.48 – 1.20)	0.238
Sex (male) * high genetic risk	0.84 (0.64 – 1.10)	0.197	0.74 (0.48 – 1.14)	0.168
Observations	155070		155070	

Stable 1b : Sex - loneliness and sex -isolation -interactions (adjusted for main effects). Figures and Hazar Ratios (HR) and 95 % confidence intervals (95% CI)

	**All dementia**		**Alzheimer's disease**	
Predictor	*HR (95% CI)*	*P-Value*	*HR (95% CI)*	*P-Value*
Sex (male) * lonelyd	1.36 (0.90 – 2.04)	0.143	2.63 (1.18 – 5.90)	0.019
Sex (male) * isolated	0.90 (0.66 – 1.24)	0.533	1.13 (0.68 – 1.90)	0.640
Observations	147610 /152719		147610 / 152719	

STable 1c: Genetic risk - loneliness and genetic risk -isolation -interactions (adjusted for age, sex and main effects). Figures and Hazar Ratios (HR) and 95 % confidence intervals (95% CI)

	**All dementia**		**Alzheimer's disease**	
Predictor	*HR (95%CI)*	*P-Value*	*HR (95%CI)*	*P-Value*
Intermediate genetic risk *lonelyd	0.86 (0.51 – 1.47)	0.586	1.71 (0.45 – 6.62)	0.431
High genetic risk * lonelyd	0.98 (0.58 – 1.65)	0.945	3.25 (0.94 – 11.28)	0.063
Observations	147610		147610	

	**All dementia**		**Alzheimer's disease**	
Predictor	*HR (95%CI)*	*P-Value*	*HR (95%CI)*	*P-Value*

For peer review only

Intermediate genetic risk * isolated	1.18 (0.77 – 1.81)	0.449	1.22 (0.59 – 2.52)	0.587
High genetic risk * isolated	1.11 (0.73 – 1.69)	0.624	1.15 (0.58 – 2.31)	0.682
Observations	152719		152719	

For peer review only

5) The associations of social isolation, loneliness and genetic risk score with specific Alzheimer's disease

We repeated all the analyses using specific Alzheimer's disease as the outcome instead of incident dementia and the results were materially the same, although there were, of course, much less Alzheimer's disease cases.

STable 2. Risk of Incident Alzheimers' Disease According to Genetic Risk

	**Model 1**		**Model 2**	
Genetic risk	*HR 95 % CI*	*P-Value*	*HR 95 % CI*	*P-Value*
Intermediate	1.51 (1.20 – 1.90)	**<0.001**	1.42 (1.11 – 1.82)	**<0.001**
High	1.98 (1.59 – 2.45)	**<0.001**	1.91 (1.51 – 2.41)	**<0.001**
Observations	155063		139345	

Model 1. Adjusted for age, sex, and 10 principal components
Model 2. Adjusted for age, sex, 10 principal components, education, social deprivation, depressive symptoms, health behaviors, loneliness and social isolation

For peer review only

STable 3. Associations of loneliness and isolation with Alzheimer' disease. The figures are Hazard ratios (HR) and 95% confidence intervals (95% CI)

	**Model 1**		**Model 2**		**Model 3**	
			Separate analyses			
Predictor	*HR (95% CI)*	*P-Value*	*HR (95% CI)*	*P-Value*	*HR (95% CI)*	*P-Value*
Lonely vs not lonely	1.05 (0.72 – 1.52)	0.809	0.85 (0.55 – 1.31)	0.450	0.86 (0.55 – 1.33)	0.503
Isolated vs no isolated	1.56 (1.21 – 2.02)	**<0.001**	1.40 (1.05 – 1.88)	**0.024**	1.41 (1.05 – 1.89)	**0.021**
			Combined analyses			
	HR (95% CI)	*P-Value*	*HR (95% CI)*	*P-Value*	*HR (95% CI)*	*P-Value*
Lonely vs not lonely	0.95 (0.64 – 1.40)	0.774	0.84 (0.51 – 1.26)	0.346	0.81 (0.52 – 1.27)	0.716
Isolated vs no isolated	1.54 (1.18 – 2.02)	**0.002**	1.41 (1.04 – 1.91)	**0.025**	1.42 (1.05 – 1.93)	**0.022**
Observations	147604 /152712		133885 /137894		133885 /137894	

Model 1. Adjusted for age and sex
Model 2. Adjusted for age, sex, education, social deprivation, health behaviours, long-term illness, depressive symptoms, genetic risk and 10 principal components
Model 3. Adjusted for age, sex, education, social deprivation, health behaviours, long-term illness, depressive symptoms, and apolipoprotein E genotype.

For peer review only

6) The associations of social isolation, loneliness and genetic risk score with incident dementia using imputed data

The number of missing values was relatively small (only less the 5% had missing values), but we repeated the final models using five imputed data sets and, not surprisingly, the results were materially not changed (sTable 4).

STable 4. Associations of loneliness and isolation with incident dementia with imputed data. The figures are Hazard ratios (HR) and 95% confidence intervals (95% CI)

	**Model 1**		**Model 2**		**Model 3**	
	Separate analyses					
Predictor	*HR (95% CI)*	*P-Value*	*HR (95% CI)*	*P-Value*	*HR (95% CI)*	*P-Value*
Lonely vs not lonely	1.44 (1.17 – 1.77)	**<0.001**	1.01 (0.81 – 1.27)	0.901	1.02 (0.82 – 1.28)	0.832
Isolated vs no isolated	1.63 (1.39 – 1.92)	**<0.001**	1.34 (1.14 – 1.58)	**0.001**	1.34 (1.14 – 1.58)	**0.001**
	Combined analyses					
	HR (95% CI)	*P-Value*	*HR (95% CI)*	*P-Value*	*HR (95% CI)*	*P-Value*
Lonely vs not lonely	1.32 (1.08 – 1.63)	**0.013**	0.97 (0.78 – 1.21)	0.804	0.98 (0.78 – 1.22)	0.844
Isolated vs no isolated	1.58 (1.35 – 1.86)	**<0.001**	1.35 (1.14– 1.59)	**0.002**	1.35 (1.15 – 1.60)	**0.003**
Observations	155063		155063		155063	

Model 1. Adjusted for age and sex
Model 2. Adjusted for age, sex, education, social deprivation, health behaviours, long-term illness, depressive symptoms, genetic risk and 10 principal components
Model 3. Adjusted for age, sex, education, social deprivation, health behaviours, long-term illness, depressive symptoms, and apolipoprotein E genotype.

For peer review only

STROBE (Strengthening The Reporting of OBservational Studies in Epidemiology) Checklist

A checklist of items that should be included in reports of observational studies. You must report the page number in your manuscript where you consider each of the items listed in this checklist. If you have not included this information, either revise your manuscript accordingly before submitting or note N/A.

Note: An Explanation and Elaboration article discusses each checklist item and gives methodological background and published examples of transparent reporting. The STROBE checklist is best used in conjunction with this article (freely available on the Web sites of PLoS Medicine at http://www.plosmedicine.org/, Annals of Internal Medicine at http://www.annals.org/, and Epidemiology at http://www.epidem.com/). Information on the STROBE Initiative is available at www.strobe-statement.org.

Section and Item	Item No.	Recommendation	Reported on Page No.
**Title and Abstract**	1	(*a*) Indicate the study's design with a commonly used term in the title or the abstract	2-3
		(*b*) Provide in the abstract an informative and balanced summary of what was done and what was found	2-3
**Introduction**			
Background/Rationale	2	Explain the scientific background and rationale for the investigation being reported	5
Objectives	3	State specific objectives, including any prespecified hypotheses	5
**Methods**			
Study Design	4	Present key elements of study design early in the paper	5-6
Setting	5	Describe the setting, locations, and relevant dates, including periods of recruitment, exposure, follow-up, and data collection	5-6
Participants	6	(*a*) *Cohort study*—Give the eligibility criteria, and the sources and methods of selection of participants. Describe methods of follow-up	6 -7
		Case-control study—Give the eligibility criteria, and the sources and methods of case ascertainment and control selection. Give the rationale for the choice of cases and controls	
		Cross-sectional study—Give the eligibility criteria, and the sources and methods of selection of participants	
		(*b*) *Cohort study*—For matched studies, give matching criteria and number of exposed and unexposed	
		Case-control study—For matched studies, give matching criteria and the number of controls per case	
Variables	7	Clearly define all outcomes, exposures, predictors, potential confounders, and effect modifiers. Give diagnostic criteria, if applicable	6-9

For peer review only

Section and Item	Item No.	Recommendation	Reported on Page No.
Data Sources/ Measurement	8*	For each variable of interest, give sources of data and details of methods of assessment (measurement). Describe comparability of assessment methods if there is more than one group	6-9
Bias	9	Describe any efforts to address potential sources of bias	9-10
Study Size	10	Explain how the study size was arrived at	6
Quantitative Variables	11	Explain how quantitative variables were handled in the analyses. If applicable, describe which groupings were chosen and why	6-9
Statistical Methods	12	(*a*) Describe all statistical methods, including those used to control for confounding	9
		(*b*) Describe any methods used to examine subgroups and interactions	9
		(*c*) Explain how missing data were addressed	9-10
		(*d*) *Cohort study*—If applicable, explain how loss to follow-up was addressed	9-10
		Case-control study—If applicable, explain how matching of cases and controls was addressed	
		Cross-sectional study—If applicable, describe analytical methods taking account of sampling strategy	
		(*e*) Describe any sensitivity analyses	9-10
**Results**			
Participants	13*	(a) Report numbers of individuals at each stage of study—eg numbers potentially eligible, examined for eligibility, confirmed eligible, included in the study, completing follow-up, and analysed	6
		(b) Give reasons for non-participation at each stage	6
		(c) Consider use of a flow diagram	
Descriptive Data	14*	(a) Give characteristics of study participants (eg demographic, clinical, social) and information on exposures and potential confounders	10
		(b) Indicate number of participants with missing data for each variable of interest	10
		(c) *Cohort study*—Summarise follow-up time (eg, average and total amount)	10
Outcome Data	15*	*Cohort study*—Report numbers of outcome events or summary measures over time	10 -11
		Case-control study—Report numbers in each exposure category, or summary measures of exposure	
		Cross-sectional study—Report numbers of outcome events or summary measures	

For peer review only

Section and Item	Item No.	Recommendation	Reported on Page No.
Main Results	16	(*a*) Give unadjusted estimates and, if applicable, confounder-adjusted estimates and their precision (eg, 95% confidence interval). Make clear which confounders were adjusted for and why they were included	10-11
		(*b*) Report category boundaries when continuous variables were categorized	6-9
		(*c*) If relevant, consider translating estimates of relative risk into absolute risk for a meaningful time period	11
Other Analyses	17	Report other analyses done—eg analyses of subgroups and interactions, and sensitivity analyses	12
**Discussion**			
Key Results	18	Summarise key results with reference to study objectives	12
Limitations	19	Discuss limitations of the study, taking into account sources of potential bias or imprecision. Discuss both direction and magnitude of any potential bias	14
Interpretation	20	Give a cautious overall interpretation of results considering objectives, limitations, multiplicity of analyses, results from similar studies, and other relevant evidence	12 -15
Generalisability	21	Discuss the generalisability (external validity) of the study results	14
**Other Information**			
Funding	22	Give the source of funding and the role of the funders for the present study and, if applicable, for the original study on which the present article is based	16-17

*Give information separately for cases and controls in case-control studies and, if applicable, for exposed and unexposed groups in cohort and cross-sectional studies.

Once you have completed this checklist, please save a copy and upload it as part of your submission. DO NOT include this checklist as part of the main manuscript document. It must be uploaded as a separate file.

For peer review only
